# WWP2 ubiquitylates RNA polymerase II for DNA-PK-dependent transcription arrest and repair at DNA breaks

**DOI:** 10.1101/gad.321943.118

**Published:** 2019-06-01

**Authors:** Pierre Caron, Tibor Pankotai, Wouter W. Wiegant, Maxim A.X. Tollenaere, Audrey Furst, Celine Bonhomme, Angela Helfricht, Anton de Groot, Albert Pastink, Alfred C.O. Vertegaal, Martijn S. Luijsterburg, Evi Soutoglou, Haico van Attikum

**Affiliations:** 1 Department of Human Genetics, Leiden University Medical Center, 2333 ZC Leiden, The Netherlands;; 2 Institut de Génétique et de Biologie Moléculaire et Cellulaire (IGBMC), 67404 Illkirch, France;; 3 U1258, Institut National de la Santé et de la Recherche Médicale (INSERM), 67404 Illkirch, France;; 4 UMR7104, Centre National de Recherche Scientifique (CNRS), 67404 Illkirch, France;; 5 Université de Strasbourg, 67081 Strasbourg, France;; 6 Department of Cell and Chemical Biology, Leiden University Medical Center, 2333 ZC Leiden, The Netherlands

**Keywords:** DNA double-strand break repair, transcription silencing, DNA-PK, WWP2 HECT E3 ubiquitin ligase, RNAPII ubiquitylation

## Abstract

DNA double-strand breaks (DSBs) at RNA polymerase II (RNAPII) transcribed genes lead to inhibition of transcription. The DNA-dependent protein kinase (DNA-PK) complex plays a pivotal role in transcription inhibition at DSBs by stimulating proteasome-dependent eviction of RNAPII at these lesions. How DNA-PK triggers RNAPII eviction to inhibit transcription at DSBs remains unclear. Here we show that the HECT E3 ubiquitin ligase WWP2 associates with components of the DNA-PK and RNAPII complexes and is recruited to DSBs at RNAPII transcribed genes. In response to DSBs, WWP2 targets the RNAPII subunit RPB1 for K48-linked ubiquitylation, thereby driving DNA-PK- and proteasome-dependent eviction of RNAPII. The lack of WWP2 or expression of nonubiquitylatable RPB1 abrogates the binding of nonhomologous end joining (NHEJ) factors, including DNA-PK and XRCC4/DNA ligase IV, and impairs DSB repair. These findings suggest that WWP2 operates in a DNA-PK-dependent shutoff circuitry for RNAPII clearance that promotes DSB repair by protecting the NHEJ machinery from collision with the transcription machinery.

## Abstract (precis)

Here, Caron et al. show that the HECT E3 ubiquitin ligase WWP2 associates with components of the DNA-PK and RNAPII complexes and is recruited to DSBs at RNAPII transcribed genes. Their findings suggest that WWP2 operates in a DNA-PK-dependent shutoff circuitry for RNAPII clearance that promotes DSB repair by protecting the NHEJ machinery from collision with the transcription machinery.

DNA double-strands breaks (DSBs) are a threat to the integrity of our genome. If left unrepaired or repaired inaccurately, they can lead to chromosomal rearrangements or loss of genetic information. While DSBs can be repaired by either homologous recombination (HR) or alternative end joining (alt-EJ), canonical nonhomologous end joining (cNHEJ) is the predominant repair pathway that seals the two broken ends together with or without minimal homology (Deriano and Roth 2013; Chang et al. 2017; Pannunzio et al. 2018). Since DSBs can occur in inactive and actively transcribed regions, an intimate interplay between these repair mechanisms and transcription is required to preserve genome stability and control transcriptional programs.

While DNA damage to the transcribed strand directly blocks RNA polymerase II (RNAPII) progression, DSBs lead to arrest of RNAPII transcription in a manner dependent on the PI3K-like kinases ataxia telangiectasia mutated (ATM) and DNA-dependent protein kinase catalytic subunit (DNA-PKcs) as well as the poly(ADP-ribose) polymerase 1 (PARP1) enzyme (Marnef et al. 2017; Ray Chaudhuri and Nussenzweig 2017). In response to clustered DSBs induced by the FokI or I-SceI endonucleases, ATM will rapidly trigger transcription silencing of DSB-flanking genes by regulating the establishment and spreading of a histone-repressive mark, H2AK119ub, and of Lys11-linked ubiquitin conjugates on H2A/H2AX. H2AK119ub is catalyzed by the E3 ubiquitin ligases RNF8/RNF168 and Ring1B, which is a component of polycomb-repressive complex 1 (PRC1) and PRC2. In addition, RNF8 is also involved in catalyzing K11-linked ubiquitin moieties on H2A/H2AX (Paul and Wang 2017). While RNF8/RNF168 recruitment relies on ATM-dependent phosphorylation of H2AX and MDC1 (Dantuma and van Attikum 2016), Ring1B is recruited through ATM-dependent phosphorylation of the superelongating complex (SEC) and the PBAF chromatin remodeling complex (Shanbhag et al. 2010; Kakarougkas et al. 2014; Ui et al. 2015). Importantly, these ATM-driven mechanisms for transcription silencing are critical for proper DSB repair through NHEJ.

Besides ATM, PARP1 also promotes transcription silencing near clustered DSBs. This involves the PARP1-dependent recruitment and activities of histone demethylase KDM5a and the ZMYND8–NuRD complex at DSBs (Chou et al. 2010; Gong et al. 2015, 2017; Spruijt et al. 2016). Moreover, PARP1 mediates recruitment of the NELF complex (Awwad et al. 2017), a negative regulator of transcription, which has been described to regulate RNAPII pausing at promoters shortly after transcription initiation (Li et al. 2013). Finally, PARP1 triggers the recruitment of chromodomain protein Y-like (CDYL1), which negatively regulates transcription through histone H3K27 methylation (Abu-Zhayia et al. 2018). While NELF promotes DSB repair via both NHEJ and HR, KDM5a, ZMYND8–NuRD, and CDYL1 promote DSB repair through HR only (Gong et al. 2015, 2017; Abu-Zhayia et al. 2018). Together, these studies revealed that ATM and PARP1 silence transcription of genes that flank DSBs by triggering extensive chromatin remodeling around the damage, thereby promoting efficient repair by NHEJ and HR. It is unclear whether these processes trigger transcription silencing by directly regulating RNAPII itself.

In the case of unique nonclustered DSBs introduced by, for instance, the I-PpoI endonuclease into transcribed genes, repression of transcription is regulated at the level of RNAPII itself and is mediated by the DNA-PK complex (Pankotai et al. 2012). Activated DNA-PK is responsible for the arrest and release of elongating RNAPII, the latter of which involves proteasome activity (Pankotai et al. 2012). However, it is unclear how DNA-PKcs triggers proteasome-dependent transcriptional silencing of broken genes. In this study, we identify the HECT E3 ubiquitin ligase WWP2 as a critical mediator of transcription silencing at DSBs. WWP2 acts in a DNA-PKcs-dependent manner to target RNAPII for ubiquitylation and subsequent degradation by the proteasome, thereby promoting transcription repression and DSB repair by cNHEJ.

## Results

### WWP2 protects cells against DSBs by promoting NHEJ

**Figure 1.**
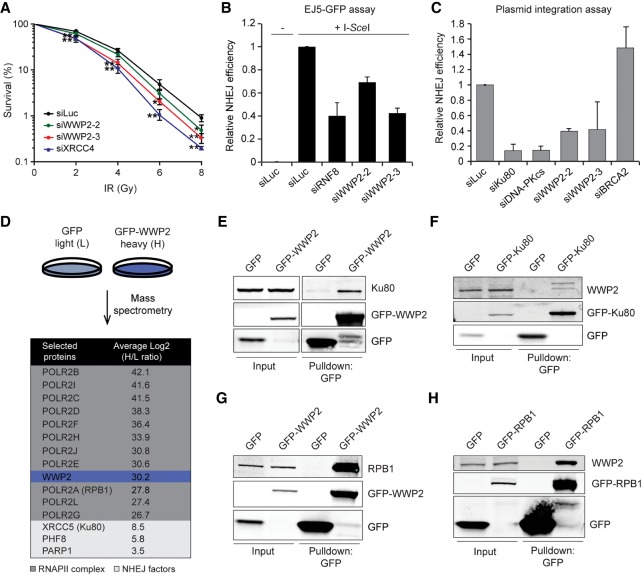
WWP2 protects cells against DSBs by promoting NHEJ. (*A*) Clonogenic survival of VH10-SV40 cells transfected with the indicated siRNAs and exposed to the indicated doses of IR. The mean ± SD from three independent experiments is shown. Statistical significance was calculated using the Student's *t*-test. (*) *P* < 0.05; (**) *P* < 0.01. (*B*) Quantification of GFP-positive EJ5-GFP HEK293 cells transfected with the indicated siRNAs. DSBs were induced by transfection of an I-SceI expression vector. The transfection efficiency was corrected by cotransfection with an mCherry expression vector. The mean ± SD from two independent experiments is shown. (*C*) Quantification of plasmid integration efficiencies in U2OS cells transfected with the indicated siRNAs. The mean ± SD from two independent experiments is shown. (*D*) SILAC (stable isotope labeling by amino acids in culture)-based mass spectrometry analysis of stable U2OS cells expressing GFP (L) or GFP-WWP2 (H). RNAPII complex members are marked in dark gray, whereas NHEJ factors are indicated in light gray. (*E*) Pull-downs of the indicated GFP fusion proteins in U2OS cells. Blots were probed for Ku80 and GFP. (*F*) Pull-downs of the indicated GFP fusion proteins in HeLa cells. Blots were probed for WWP2 and GFP. (*G*) As in *E*, except that blots were probed for RPB1 and GFP. (*H*) As in *E*, except that blots were probed for WWP2 and GFP.

An RNAi-based genome-wide screen in *Caenorhabditis elegans* identified *Ce*-*wwp-1* as a novel gene that protects cells against ionizing radiation (IR) (van Haaften et al. 2006). We assessed whether the human homolog of *Ce*-*wwp-1*, the *WWP2* gene, plays a similar role. To this end, two independent siRNAs were used to deplete WWP2 in VH10-SV40 immortalized human fibroblasts (Supplemental Fig. S1A), and clonogenic survival of these cells was determined following exposure to IR. WWP2-depleted cells were markedly more sensitive to IR when compared with control cells (siLuc), although not to the same extent as cells depleted of the core NHEJ factor XRCC4 (Fig. 1A). Thus, WWP2 protects human cells against the clastogenic effects of IR (van Haaften et al. 2006), suggesting a role for WWP2 in the repair of IR-induced DNA damage.

IR induces a variety of DNA lesions, including highly deleterious DNA DSBs, which are predominantly repaired by NHEJ. To determine whether WWP2 affects this repair process, we used the well-established EJ5-GFP assay for NHEJ repair of I-SceI nuclease-induced DSBs (Supplemental Fig. 1B; Bennardo et al. 2008). Depletion of RNF8, an E3 ubiquitin ligase known to be involved in NHEJ (Butler et al. 2012), greatly reduced NHEJ (Fig. 1B). Importantly, we also found that depletion of WWP2 markedly reduced NHEJ (Fig. 1B; Supplemental Fig. S1C). The EJ5-GFP reporter provides a readout for total NHEJ activity (cNHEJ and alternative NHEJ) (Bennardo et al. 2008). To examine whether WWP2 plays a role specifically in cNHEJ, we monitored random plasmid integration into the human genome, which we and others have shown to be largely dependent on cNHEJ (Supplemental Fig. S1D; Galanty et al. 2009; Agarwal and Jackson 2016; Luijsterburg et al. 2016). Indeed, depletion of the core cNHEJ factors Ku80 and DNA-PKcs dramatically reduced cNHEJ (Fig. 1C), whereas depletion of BRCA2, required for HR-mediated DSB repair, did not impair this process (and may even lead to a slight increase) (Luijsterburg et al. 2016). Importantly, WWP2 depletion decreased the NHEJ efficiency by ∼60%. Thus, our results suggest that WWP2 is a novel factor that promotes DSB repair by NHEJ.

### WWP2 interacts with NHEJ proteins and members of the RNAPII complex

To study how WWP2 affects DSB repair, we set out to identify proteins that interact with WWP2. To this end, we generated U2OS cells stably expressing GFP-tagged WWP2. Pull-downs of GFP-WWP2 from these cells followed by mass spectrometry (MS) after stable isotope labeling by amino acids in culture (SILAC) revealed 621 proteins that were at least twofold enriched compared with control cells (Supplemental Table S1). Our analysis revealed Ku80, PARP1, and the histone demethylase PHF8, all of which regulate DSB repair by NHEJ (Fig. 1D; Fell and Schild-Poulter 2015; Luijsterburg et al. 2016; Wang et al. 2016). In addition, we also identified 11 of the 12 subunits of the RNAPII complex (Fig. 1D; Wild and Cramer 2012). Among these was RPB1 (POLR2A), whose phosphorylation and ubiquitylation are critical for transcription regulation under physiological as well as DNA damage conditions (Ratner et al. 1998; Somesh et al. 2005, 2007; Sordet et al. 2008; Yasukawa et al. 2008; Verma et al. 2011; Hsin and Manley 2012; Jeronimo et al. 2016).

Reciprocal GFP pull-downs coupled to Western blot analysis confirmed that GFP-tagged WWP2 interacts with endogenous Ku80 in U2OS cells and that GFP-tagged Ku80 interacts with endogenous WWP2 in HeLa cells (Fig. 1E,F). Moreover, using the same approach, we also confirmed the interaction between GFP-tagged WWP2 and endogenous RPB1 (Fig. 1G). To confirm the interaction between GFP-WWP2 and RPB1 in a reciprocal manner, we established U2OS cells stably expressing GFP-tagged RPB1 that is resistant to the RNAPII inhibitor α-amanitin (Supplemental Fig. S1E,F; Darzacq et al. 2007; Dias et al. 2015). Expression of endogenous RPB1 was lost in these cells upon treatment with α-amanitin (Supplemental Fig. S1E,F). Moreover, we detected the elongating form of GFP-RPB1 (p-GFP-RPB1 S2), indicating that GFP-tagged RPB1 functionally replaced endogenous RPB1 in these cells. Importantly, using these cells, we also observed that GFP-RPB1 interacts with endogenous WWP2 (Fig. 1H). Together, our results show that WWP2 not only interacts with the core NHEJ factor Ku80 but also associates with the RNAPII complex, the latter of which agrees with a previous report (Li et al. 2007). Moreover, these findings suggest a potential role for WWP2 in regulating RNAPII during NHEJ.

### WWP2 is recruited to DSBs in transcribed genes to promote DNA repair

**Figure 2.**
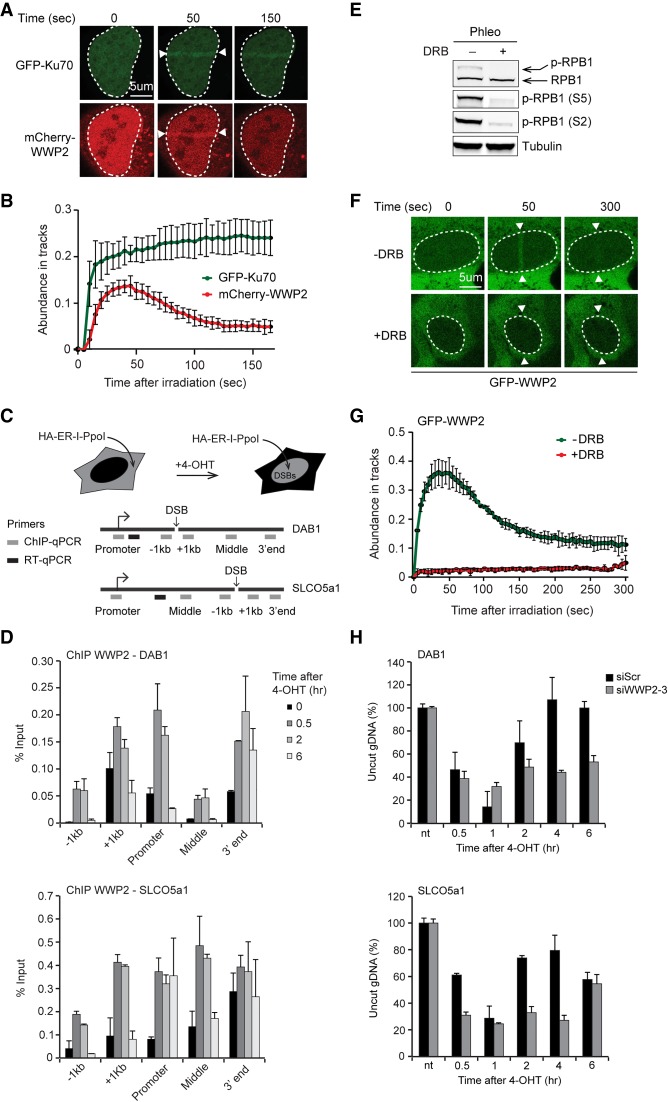
WWP2 is recruited to DSBs in transcribed genes to promote DNA repair. (*A*) Recruitment of mCherry-WWP2 to multiphoton tracks in U2OS cells. GFP-Ku70 was used as a DNA damage marker. (*B*) Quantification of *A*. (*C*) Schematic of the HA-ER-I-PpoI system in U2OS cells used to generate site-specific DSBs at the indicated genes following 4-hydroxytamoxifen (4-OHT) treatment. Gray boxes indicate positions where protein binding is monitored by ChIP-qPCR (chromatin immunoprecipitation [ChIP] combined with quantitative PCR [qPCR]). Black boxes indicate positions of the primers used to quantify mRNA levels of the indicated genes by RT-qPCR. (*D*) ChIP-qPCR against WWP2 in U2OS HA-ER-I-PpoI cells at the indicated time points after 4-OHT treatment and at the indicated positions at *DAB1* and *SLCO5a1*. The mean ± SD from qPCR replicates of a representative experiment is shown. A repeat of the experiment is shown in Supplemental Figure S2C. (*E*) Western blot analysis of RPB1 and Ser2- and Ser5-phosphorylated RPB1 (S2 and S5) levels in phleomycin (Phleo)-treated U2OS cells that were left untreated or were treated with 5,6-dichloro-1-β-D-ribofuranosylbenzimidazole (DRB). Tubulin was used as a loading control. (*F*) Recruitment of GFP-WWP2 to multiphoton tracks in untreated and DRB-treated U2OS cells. (*G*) Quantification of *F*. (*H*) Cutting efficiencies at *DAB1* and *SLCO5a1* at the indicated time points after 4-OHT treatment in U2OS HA-ER-I-PpoI cells transfected with the indicated siRNAs. The mean ± SD from qPCR replicates of a representative experiment is shown. A repeat of the experiment is shown in Supplemental Figure S8A.

WWP2 has been shown to play a role in transcription regulation (Li et al. 2007; Marcucci et al. 2011; Scheffner and Kumar 2014). This raised the possibility that WWP2 affects DSB repair indirectly by regulating the RNAPII-dependent expression of NHEJ factors. However, we found that the expression of several factors involved in NHEJ was comparable with that in control cells (Supplemental Fig. S2A). Alternatively, WWP2 may play a direct role in NHEJ by acting at sites of DNA damage. To examine this, we monitored whether WWP2 is recruited to multiphoton laser-inflicted DNA damage. U2OS cells were cotransfected with expression vectors for mCherry-tagged WWP2 and GFP-tagged Ku70, a core NHEJ factor that served as a positive control for recruitment. Live-cell imaging after laser microirradiation indeed revealed that, similar to GFP-Ku70, mCherry-WWP2 rapidly accumulates at sites of DNA damage (Fig. 2A,B; Kochan et al. 2017). However, whereas GFP-Ku70 reached maximum levels of accumulation at 100 sec and remained associated with the DNA damage during the course of the experiment, mCherry-WWP2 transiently associated, reaching maximum levels at 50 sec and returning to near-basal levels at 150 sec (Fig. 2B). Similar recruitment dynamics were observed in stable cells expressing GFP-WWP2 (Fig. 2F,G).

Since WWP2 interacts with the RNAPII complex, we next addressed whether it is recruited to bona fide DSBs that occur within transcribed genes. To explore this possibility, we expressed the site-specific I-PpoI meganuclease tagged with HA and estrogen receptor (ER) from a doxycycline (Dox)-inducible promoter in U2OS cells (U2OS-pEP15) to introduce a unique DSB in several transcribed genes (Fig. 2C; Supplemental Fig. S2B; Pankotai et al. 2012). We then performed chromatin immunoprecipitation (ChIP) experiments against endogenous WWP2 and monitored its levels before and at different time points after DSB induction in two of the actively transcribed genes: *DAB1* and *SLCO5a1*. Two other actively transcribed genes, *INTS4* and *p21*, without DSB served as controls. We found that WWP2 is recruited to DSBs induced at *DAB1* and *SLCO5a1*, reaching maximum levels between 30 min and 2 h and returning to near-basal levels at 6 h at all positions except for the 3′ end of these genes (Fig. 2D; Supplemental Fig. S2C). In contrast, WWP2 did not accumulate at the nondamaged *INTS4* and *p21* genes (Supplemental Fig. S2D). Next, we asked whether the recruitment of WWP2 to DSBs in active genes is dependent on RNAPII-driven transcription. To this end, stable GFP-WWP2 cells were treated with the DSB-inducing agent phleomycin and 5,6-dichloro-1-β-D-ribofuranosylbenzimidazole (DRB), which inhibits RNAPII transcription as revealed by a reduction in the levels of Ser5-phosphorylated (initiating form) and Ser2-phosphorylated (elongating form) RPB1 (Fig. 2E; Jeronimo et al. 2016). Laser microirradiation of these cells showed that DRB treatment completely abrogated the transient recruitment of GFP-WWP2 (Fig. 2F,G), indicating that active RNAPII-mediated transcription is required for the accumulation of WWP2 at sites of DNA damage.

We then determined whether loss of WWP2 may impact the efficiency of DSB repair in *DAB1* and *SLCO5a1* as well as in another actively transcribed gene, *RYR2*, which can be cleaved by I-PpoI. To this end, we used our previously established quantitative PCR (qPCR)-based assay, which determines DSB repair by comparing the amplification of DNA products across the I-PpoI cleavage sites before and after DSB induction (Pankotai et al. 2012). DSB induction reached a plateau between 30 min and 1 h, while repair of the breaks was detected after 4–6 h in control cells (siScr) (Fig. 2H; Supplemental Figs. S2E,F, S8A). Importantly, depletion of WWP2 did not affect the efficiency of DSB formation, as monitored by our qPCR-based assay as well as by ChIP for γH2AX (Supplemental Figs. S2G,H, S9). However, we found that most DSBs remained unrepaired at 4–6 h after DSB induction, suggesting that the loss of WWP2 strongly impacted the repair of these lesions (Fig. 2H; Supplemental Figs. S2E,F, S8A). Together, these results demonstrate that WWP2 is recruited to DSBs in actively transcribed genes to promote efficient repair of these DNA lesions.

### WWP2 represses transcription following DSB induction in active genes

**Figure 3.**
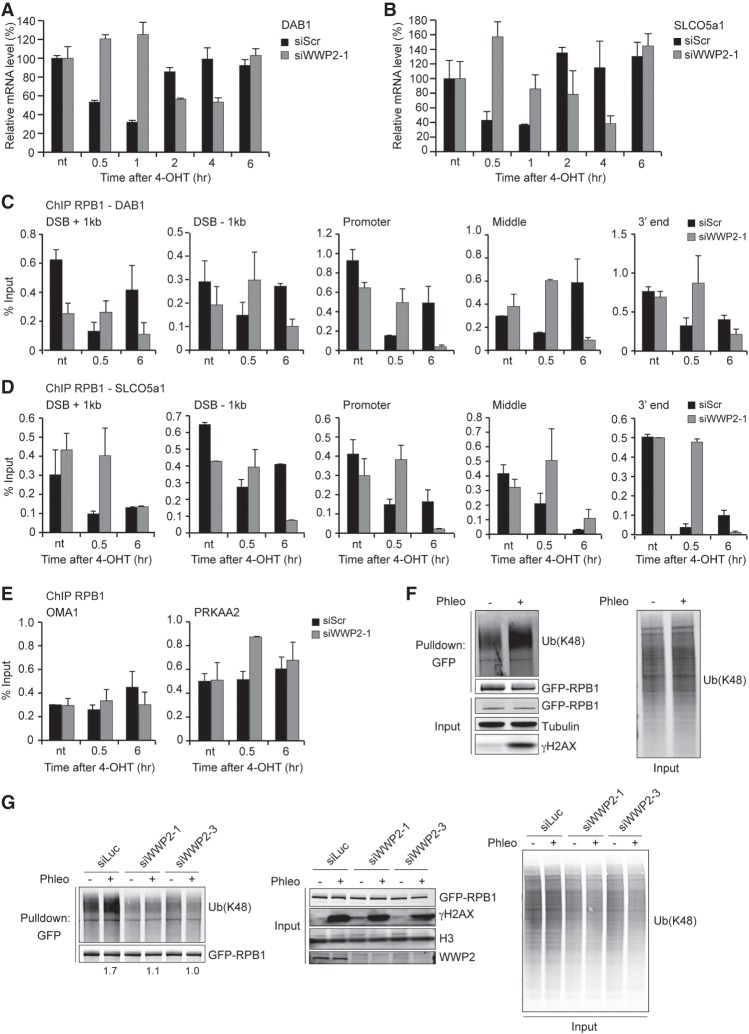
WWP2 promotes DSB-induced transcription silencing and RPBI ubiquitylation after DNA damage. (*A*) RT-qPCR analysis of *DAB1* expression levels in U2OS HA-ER-I-PpoI cells at the indicated time points after 4-OHT treatment and transfected with the indicated siRNAs. *DAB1* mRNA levels were normalized to those of cyclophilin B. The mean ± SD from qPCR replicates of a representative experiment is shown. A repeat of the experiment is shown in Supplemental Figure S8B. (*B*) As in *A*, except for *SLCO5a1*. A repeat of the experiment is shown in Supplemental Figure S8B. (*C*) ChIP-qPCR against RPB1 in U2OS HA-ER-I-PpoI cells transfected with the indicated siRNAs. RPB1 levels were monitored at the indicated time points after 4-OHT treatment and at the indicated positions at *DAB1*. The mean ± SD from qPCR replicates of a representative experiment is shown. A repeat of the experiment is shown in Supplemental Figure S8C. (*D*) As in *C*, except for *SLCO5a1*. A repeat of the experiment is shown in Supplemental Figure S8D. (*E*) ChIP-qPCR against RPB1 in U2OS HA-ER-I-PpoI cells transfected with the indicated siRNAs. RPB1 levels were monitored at the indicated time points after 4-OHT treatment at the *OMA1* and *PRKAA2* genes. The mean ± SD from qPCR replicates of a representative experiment is shown. A repeat of the experiment is shown in Supplemental Figure S8E. (*F*) Pull-downs of GFP-RPB1 under denaturing conditions in untreated and phleomycin (Phleo)-treated U2OS cells. Cells were also treated with proteasome inhibitor (MG-132) 25 min before the phleomycin treatment. Blots were probed for Ub(K48), GFP, and γH2AX. Tubulin was used as a loading control. (*G*) As in *F*, except that cells were treated with the indicated siRNAs, and blots were also probed for H3. Relative Ub(K48) levels after GFP-RPB1 pull-down from phleomycin-treated versus untreated cells are indicated *below* the blots.

We reported previously that DSBs within transcribed genes induce transcription arrest through RNAPII eviction in *cis* (Pankotai et al. 2012). In order to assess a potential role of WWP2 in this process, we first measured the mRNA levels of *DAB1*, *SLCO5a1*, and *RYR2* before and after DSB induction by I-PpoI using RT-qPCR. We observed a rapid and strong decrease of the mRNA levels between 30 min and 1 h after DSB induction, while a return to basal levels was detected between 4 and 6 h when repair of the damage was achieved (Fig. 3A,B, Supplemental Figs. S3A, S8B). However, following WWP2 depletion, mRNA levels remained stable for at least 1–2 h after DSB induction and decreased only after 4 h, returning to basal levels at 6 h. These results suggest that WWP2 mediates an efficient arrest of transcription at broken genes.

Inhibition of nascent transcription at sites of DNA damage inflicted by UV-A laser microirradiation was observed by monitoring the levels of nascent transcripts using 5-ethynyl uridine (5-EU) incorporation (Supplemental Fig. S3B; Gong et al. 2015). Using this approach, we also found that in control cells, the transcription arrest at DNA damage sites is manifested by a decrease in EU incorporation (Supplemental Fig. S3C,D). However, the levels of nascent transcripts did not decrease dramatically when either CHD4 (a positive control) or WWP2 was depleted (Supplemental Fig. S3C,D), confirming that WWP2 promotes transcription silencing at sites of DNA damage.

Next, we examined whether WWP2 regulates transcription arrest at broken genes by affecting RNAPII occupancy. To this end, we performed ChIP against RPB1 and measured its levels at different positions around the I-PpoI-induced DSBs in *DAB1* and *SLCO5a1*. We found that the level of RPB1 dramatically decreases along the broken genes at 30 min after DSB induction (Fig. 3C,D; Supplemental Fig. S8C,D). Importantly, following WWP2 depletion, we did not detect a rapid and strong RPB1 decrease at 30 min but rather at 6 h after DSB induction. In contrast, RPB1 occupancy at two actively transcribed *DAB1*-flanking genes—*OMA1* and *PRKAA2*, which lack I-PpoI cleavage sites (Pankotai et al. 2012)—was unchanged following DSB induction at *DAB1* irrespective of WWP2 depletion (Fig. 3E; Supplemental Fig. S8E). Altogether, these results reveal that efficient transcription arrest at broken genes is mediated by WWP2-dependent RNAPII eviction in *cis*.

### DSBs induce RPB1 ubiquitylation through WWP2

Given that WWP2 is a HECT E3 ubiquitin ligase, we next asked whether WWP2 could regulate RNAPII at DSBs by targeting one or more components of the RNAPII complex for ubiquitylation. In mice, it was shown that WWP2 can ubiquitylate the RPB1 subunit of RNAPII, thereby targeting it for proteasomal degradation (Li et al. 2007). This raised the possibility that human RPB1 also becomes targeted by WWP2, possibly in response to DSBs, as a mean to evict RNAPII from these lesions. To investigate this, we first examined whether RPB1 becomes ubiquitylated in response to DSB induction. U2OS cells stably expressing GFP-RPB1 were exposed to phleomycin, etoposide, doxorubicin, and neocarzinostatin, which are agents that induce DSBs (Goodarzi et al. 2008; Mehta and Haber 2014; Yang et al. 2015). Cells were also exposed to UV irradiation, which generates photolesions that have been shown previously to trigger ubiquitylation of RPB1 (Bregman et al. 1996; Ratner et al. 1998). Subsequently, GFP pull-downs were performed under denaturing conditions, after which the ubiquitylation status of RPB1 was monitored. RPB1's ability to interact with other proteins, such as the RNAPII subunit RPB2, was impaired under these conditions (Supplemental Fig. S3E). Moreover, we detected a clear increase in the ubiquitylation of RPB1 following UV irradiation (Supplemental Fig. S3F), agreeing with earlier work and validating our experimental setup (Bregman et al. 1996; Ratner et al. 1998). Interestingly, we found that the exposure of cells to phleomycin, etoposide, doxorubicin, or neocarzinostatin triggers robust K48-linked ubiquitylation of RPB1, suggesting that this posttranslational modification of RPB1 can be induced by DSBs (Fig. 3F; Supplemental Fig. S3G,H). However, following WWP2 depletion, we found the phleomycin-induced RPB1 K48-linked ubiquitylation to be dramatically impaired (Fig. 3G). Reciprocal pull-downs using tandem ubiquitin-binding entities (TUBEs) confirmed that RPB1 is ubiquitylated following DSB induction by phleomycin and that this process is impaired when WWP2 is depleted (Supplemental Fig. S3I). These findings demonstrate that DSBs can trigger RPB1 ubiquitylation in a manner dependent on the WWP2 HECT E3 ubiquitin ligase.

### DNA-PK shuts off transcription through WWP2-dependent RPB1 ubiquitylation

**Figure 4.**
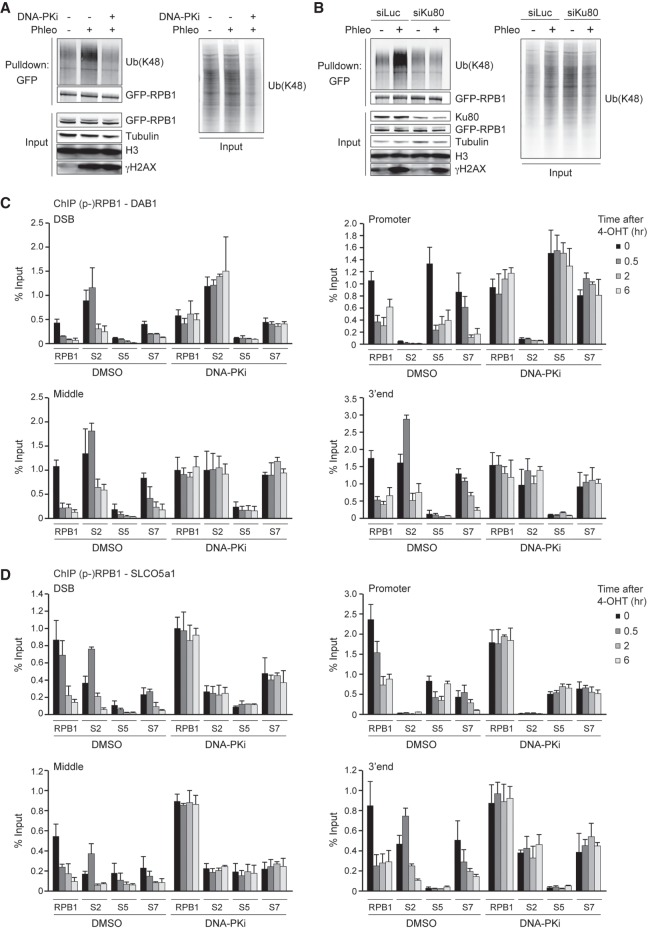
DNA-PK affects the ubiquitylation and occupancy of RPB1. (*A*) Pull-downs of GFP-RPB1 under denaturing conditions in phleomycin (Phleo)- and DNA-PK inhibitor (DNA-PKi)-treated U2OS cells. Cells were also treated with proteasome inhibitor (MG-132) 25 min before the phleomycin treatment. Blots were probed for Ub(K48), GFP, H3, and γH2AX. Tubulin was used as a loading control. (*B*) As in *A*, except that cells were transfected with the indicated siRNA. (*C*) ChIP-qPCR against RPB1 and S2-, S5-, or S7-phosphorylated RPB1 (p-RPB1) in DMSO-treated (control) and DNA-PKi-treated U2OS HA-ER-I-PpoI cells at the indicated time points after 4-OHT treatment and at the indicated positions at *DAB1*. A representative experiment is shown. A repeat of the experiment is shown in Supplemental Figures S10A and S11A. (*D*) ChIP-qPCR against RPB1 and S2-, S5-, or S7-phosphorylated RPB1 (p-RPB1) in DMSO-treated (control) and DNA-PKi-treated U2OS HA-ER-I-PpoI cells at the indicated time points after 4-OHT treatment and at the indicated positions at *SLCO5a1*. A representative experiment is shown. A repeat of the experiment is shown in Supplemental Figures S10B and S11B.

We reported previously that DSB-induced transcription arrest is regulated by the DNA-PK complex (Pankotai et al. 2012), whose kinase activity can trigger the eviction of RNAPII from broken genes. However, it remained unclear whether DNA-PK affects this process by regulating RPB1 ubiquitylation. To examine this, we performed GFP pull-downs using U2OS cells stably expressing GFP-RPB1. The cells were treated with phleomycin in the absence and presence of an inhibitor against DNA-PK. Western blot analysis detected a strong K48-linked ubiquitylation of RPB1 after phleomycin, which was dramatically reduced following DNA-PK inhibition (Fig. 4A). This result was confirmed in reciprocal pull-downs using the TUBE approach after DNA-PK depletion (Supplemental Fig. S4A,B). In line with this finding, we also observed that the depletion of DNA-PKcs or Ku80, an essential component of the DNA-PK complex, abolished RPB1 ubiquitylation induced by phleomycin (Fig. 4B; Supplemental Fig. S4C). In contrast, depletion of the cNHEJ ligase LigIV did not affect phleomycin-induced RPB1 ubiquitylation, suggesting that DNA-PK is the key NHEJ factor that regulates this process (Supplemental Fig. S4C).

Given that the phleomycin-induced ubiquitylation of RPB1 also relies on WWP2, we examined how DNA-PK and WWP2 cooperate to regulate this process. To this end, we inhibited DNA-PK in cells depleted of WWP2 and examined RBP1's ubiquitylation status following phleomycin treatment. As expected, DNA-PK inhibition or depletion of WWP2 alone reduced DNA damage-induced RPB1 ubiquitylation. Strikingly, the combined loss of DNA-PK activity and WWP2 protein did not aggravate this effect (Supplemental Fig. S4D). These results suggest that DNA-PK inhibits transcription of broken genes by regulating the WWP2-dependent ubiquitylation of the RNAPII subunit RPB1. To assess whether DNA-PK and WWP2 specifically affect RPB1 ubiquitylation or impact K48 ubiquitylation more globally, we monitored their effect on K48 ubiquitylation at laser-induced DNA damage tracks. Remarkably, we found that DNA-PK inhibition or WWP2 depletion did not impact the levels of K48 ubiquitylation in these tracks (Supplemental Fig. S4E–G). We infer that WWP2 and DNA-PK most prominently affect RPB1 ubiquitylation at DSBs, although we cannot exclude the possibility that WWP2 (possibly in a DNA-PK-dependent manner) targets DSB-associated proteins other than RPB1.

DSBs lead to the eviction of RPB1 not only proximal to DSB sites but also along broken genes. We therefore wondered whether the different steps of transcription, initiation, and elongation would be differentially affected by DSBs (Epshtein and Nudler 2003; Pankotai et al. 2012). To answer this question, we performed ChIP experiments against initiating (phospho-S5-RPB1), elongating (phospho-S2-RPB1), or initiating and elongating (phospho-S7-RPB1) RPB1 (Jeronimo et al. 2016). Similar to RPB1, all phospho-RPB1 forms (S2, S5, and S7) were dramatically reduced after DSB induction along the entire gene, reaching maximum loss at 2 h (Fig. 4C,D; Supplemental Figs. S5A, S10A,B, S11A,B). However, DNA-PK inhibition did not lead to any decrease in RPB1 and phospho-RPB1 (S2, S5, and S7) levels (Fig. 4C,D; Supplemental Figs. S5A, S10A,B, S11A,B). In contrast, the occupancy of RPB1 and phospho-RPB1 (S2, S5, and S7) on the *OMA1* and *PRKAA2* genes, which are in close proximity to the I-PpoI-induced DSB at *DAB1* and within the γH2AX-enriched domains induced by this break, was unchanged irrespective of DNA-PK inhibition (Supplemental Figs. S5B,C, S12A,B). Together, these results show that DNA-PK is required to repress RNAPII transcription at DSBs by triggering WWP2-dependent K48-linked ubiquitylation and eviction of RPB1.

### Proteasomes are recruited to broken genes to target RNAPII complexes

We next asked how the K48-linked ubiquitylation of RPB1 could lead to the eviction of RNAPII from broken genes. Polyubiquitylation and degradation of RNAPII by the proteasome system has been shown to resolve stalled RNAPII complexes on chromatin (Wilson et al. 2013). Moreover, we reported previously that the proteasome is required to negatively regulate mRNA levels of genes containing a DSB (Pankotai et al. 2012). However, it was unclear whether the proteasome is required to remove RNAPII from chromatin following the induction of DSBs. To examine this, we monitored the levels of phospho-RPB1 (S2, S5, and S7) in chromatin-enriched extracts from cells that were treated with neocarzinostatin in either the presence or absence of proteasome inhibitor MG-132. DSBs triggered a rapid and strong decrease of phospho-RPB1 (S2, S5, and S7) levels on chromatin (Supplemental Fig. S6A, left panel), in agreement with our previous work (Pankotai et al. 2012). In addition, we found that MG-132-mediated proteasome inhibition abolished this effect (Supplemental Fig. S6A, right panel). Similarly, DNA-PK inhibition also impaired phospho-RPB1 release from damaged chromatin (Supplemental Fig. S5D), which is consistent with our finding that DNA-PK activity is required to evict phospho-RPB1 from genes following DSB induction by the I-PpoI nuclease (Fig. 4C,D). Together, these findings suggest a role for the proteasome in the release of RPB1 from genes containing DSBs.

**Figure 5.**
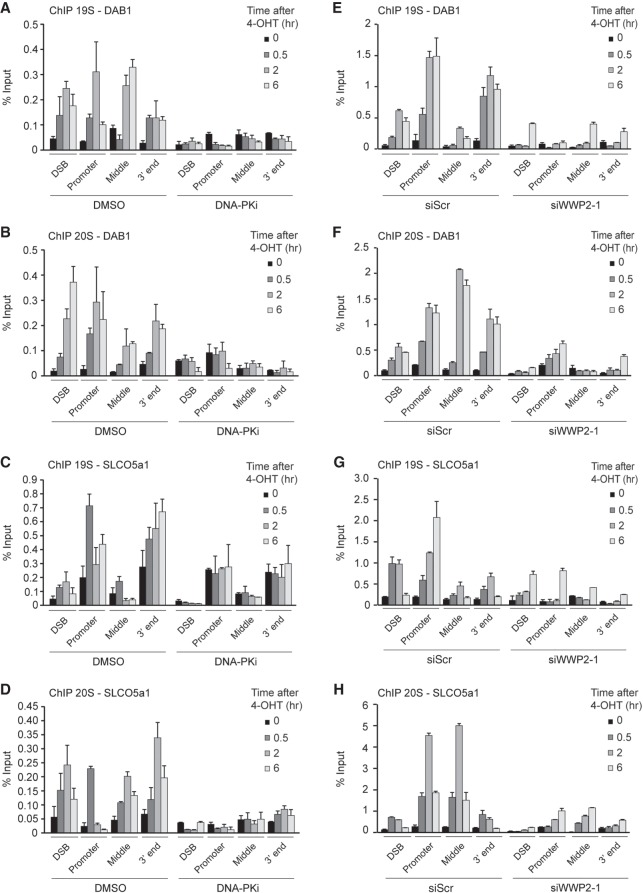
Proteasomes are recruited to broken genes in a DNA-PKcs- and WWP2-dependent manner. (*A*) ChIP-qPCR against the 19S proteasome in DMSO-treated (control) and DNA-PKi-treated U2OS HA-ER-I-PpoI cells at the indicated time points after 4-OHT treatment and at the indicated positions at *DAB1*. The mean ± SD from qPCR replicates of a representative experiment is shown. A repeat of the experiment is shown in Supplemental Figure S13A. (*B*) As in *A*, except that the 20S proteasome was examined. A repeat of the experiment is shown in Supplemental Figure S13A. (*C*) As in *A*, except for *SLCO5a1*. A repeat of the experiment is shown in Supplemental Figure S13B. (*D*) As in *B*, except for *SLCO5a1*. A repeat of the experiment is shown in Supplemental Figure S13B. (*E*) ChIP-qPCR against the 19S proteasome in U2OS HA-ER-I-PpoI cells transfected with the indicated siRNA at the indicated time points after 4-OHT treatment and at the indicated positions at *DAB1*. The mean ± SD from qPCR replicates of a representative experiment is shown. A repeat of the experiment is shown in Supplemental Figure S14A. (*F*) As in *E*, except that the 20S proteasome was examined. A repeat of the experiment is shown in Supplemental Figure S14A. (*G*) As in *E*, except for *SLCO5a1*. A repeat of the experiment is shown in Supplemental Figure S14B. (*H*) As in *F*, except for *SLCO5a1*. A repeat of the experiment is shown in Supplemental Figure S14B.

Such a scenario would imply a role for the proteasome directly at DSBs. Indeed, proteasome components have been shown to be recruited to DSBs in yeast (Krogan et al. 2004) and to sites of laser-induced DNA damage in human cells (Galanty et al. 2012). However, whether the proteasome acts at bona fide DSBs in human cells remained unclear. We therefore monitored the levels of the proteasome subcomplexes 19S and 20S at I-PpoI-induced DSBs in the *DAB1* and *SLCO5a1* genes by ChIP. Both proteasome subcomplexes accumulated near the DSBs and along the entire broken gene, reaching maximum levels mostly at ∼2 h after damage induction (Fig. 5A–H; Supplemental Figs. S13A,B, S14A,B). Proteasome levels did not increase on transcribed genes flanking *DAB1* (*OMA1* and *PRKAA2*) (Supplemental Figs. S6B,C, S13C, S14C), indicating that proteasome accumulation at *DAB1* and *SLCO5a1* is dependent on DSB induction. Finally, we found that DNA-PK inhibition or WWP2 depletion abolished the recruitment of these proteasome components to DSBs in these actively transcribed genes (Fig. 5A–H; Supplemental Figs. S13A,B, S14A,B). These findings demonstrate that DNA-PK and WWP2 trigger recruitment of the proteasome to DSBs in actively transcribed genes to promote eviction of RNAPII by acting on ubiquitylated RPB1.

### WWP2 promotes the accumulation of core NHEJ factors at DNA damage

**Figure 6.**
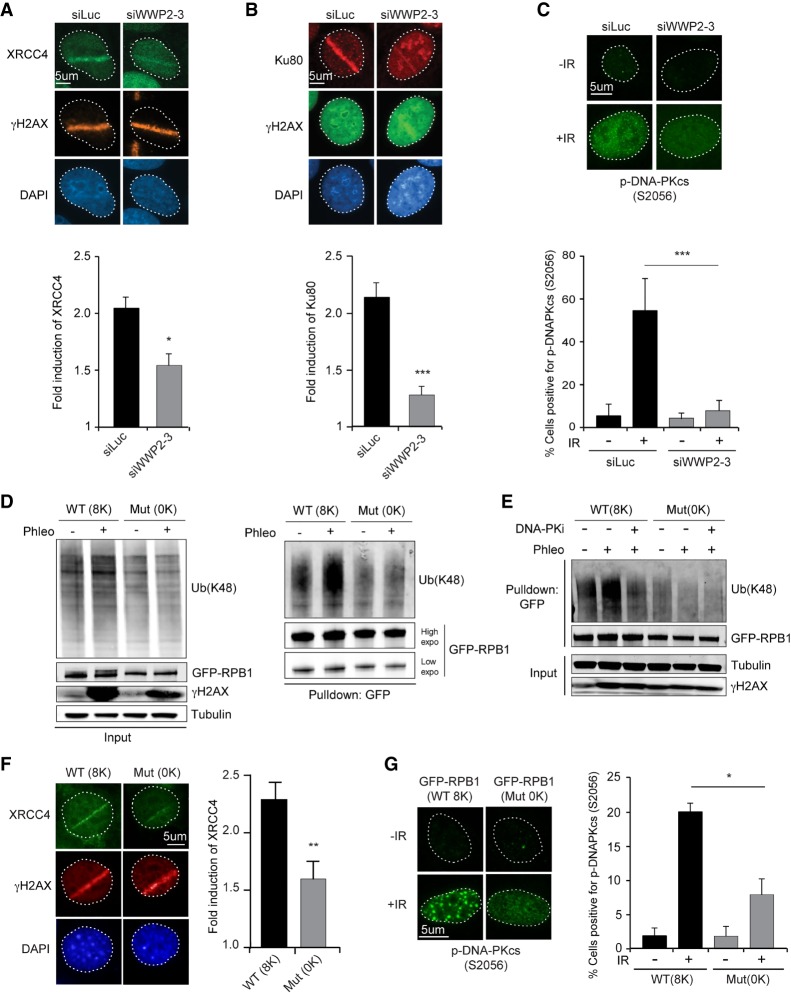
WWP2-dependent RPB1 ubiquitylation promotes accumulation of NHEJ factors at DSBs. (*A*) Immunofluorescence (IF) images (*top* panel) and quantification (*bottom* panel) of XRCC4 recruitment to DNA damage tracks generated by UV-A laser microirradiation in U2OS cells transfected with the indicated siRNAs. γH2AX was used as a DNA damage marker. The mean ± SD from three independent experiments is shown. Statistical significance was calculated using the Student's *t*-test. (*) *P* < 0.05. (*B*) As in *A*, except for Ku80. The mean ± SD from six independent experiments is shown. Statistical significance was calculated using the Student's *t*-test. (***) *P* < 0.001. (*C*) IF images (*top* panel) and quantification (*bottom* panel) of p-DNA-PKcs (S2056) focus formation 1 h after 10 Gy of IR in U2OS cells transfected with the indicated siRNAs. The mean ± S.E.M from four independent experiments is shown. Statistical significance was calculated using the Student's *t*-test. (***) *P* < 0.001. (*D*) Pull-downs of GFP-RPB1 wild type (8K) or mutant (0K) under denaturing conditions in untreated and phleomycin (Phleo)-treated NIH3T3 cells. Cells were also treated with proteasome inhibitor (MG-132) 25 min before the phleomycin treatment. Blots were probed for Ub(K48), GFP, and γH2AX. Tubulin was used as a loading control. (*E*) As in *D*, except that cells were also treated with DNA-PKi. (*F*) IF images (*left* panel) and quantification (*right* panel) of XRCC4 recruitment to DNA damage tracks generated by UV-A laser microirradiation in NIH3T3 cells expressing wild-type (8K) or mutant (0K) GFP-RPB1. The mean ± SEM from three independent experiments is shown. Statistical significance was calculated using the Student's *t*-test. (**) *P* < 0.01. (*G*) IF images (*left* panel) and quantification (*right* panel) of p-DNA-PKcs (S2056) focus formation 1 h after 10 Gy of IR in NIH3T3 cells expressing wild-type (8K) or mutant (0K) GFP-RPB1. The mean ± SEM from three independent experiments is shown. Statistical significance was calculated using the Student's *t*-test. (*) *P* < 0.05.

We showed that WWP2 promotes both NHEJ and RPB1 ubiquitylation at DSBs. However, it is not clear how WWP2 affects NHEJ and how this is linked to its role in RPB1 ubiquitylation. NHEJ relies on the binding and retention of the heterodimer Ku70/Ku80 at DSB ends, which allows for the recruitment and activation of DNA-PKcs. This in turn recruits the XRCC4/LigIV complex, which ultimately seals the break (Blackford and Jackson 2017). To assess how WWP2 affects NHEJ, we first determined the contribution of WWP2 to the accumulation of XRCC4 and Ku80 at DSBs inflicted by UV-A laser microirradiation. Indeed, depletion of WWP2 significantly reduced the recruitment of both core NHEJ proteins (Fig. 6A,B), while DNA damage induction was comparable, as monitored by the accumulation of the DSB sensor protein NBS1 (Supplemental Fig. S7A–C). To confirm this finding, we performed chromatin-binding assays to measure the association of NHEJ factors with damaged chromatin following exposure of cells to phleomycin. We observed a strong accumulation of NHEJ factors 1 h after phleomycin treatment in the histone H3-enriched chromatin fraction (Supplemental Fig. S7D,E). Again, we found that depletion of WWP2 strongly impaired the recruitment of both Ku70 and XRCC4 to damaged chromatin (Supplemental Fig. S7D,E). Finally, we also found that IR-induced phospho-DNA-PKcs (S2056), but not γH2AX, focus formation is strongly impaired after WWP2 depletion (Fig. 6C; Supplemental Fig. S7F–H). Collectively, these findings demonstrate that WWP2 promotes the efficient assembly of NHEJ factors at DSBs, thereby stimulating efficient DNA repair.

### The C-terminal domain (CTD) of RPB1 is ubiquitylated in response to DSBs to promote NHEJ

We next investigated how the role of WWP2 in recruiting NHEJ factors may be linked to its impact on RBP1 ubiquitylation and the subsequent eviction of RNAPII during transcription repression at DSBs. To this end, we first examined which residues in RPB1 could contribute to its ubiquitylation by WWP2 following DSB induction. Studies in mice suggested that WWP2 targets RPB1 on eight lysines that reside in the nonconsensus sequence of its CTD (Li et al. 2007). However, those observations did not exclude the possibility that WWP2 may ubiquitylate RPB1 by targeting one or several of the other 97 lysine residues distributed along the protein. To resolve this issue, we used mouse NIH3T3 cell lines stably expressing α-amanitin-resistant wild-type GFP-RPB1 (8K) or mutant GFP-RPB1 (0K) in which the eight lysine residues in the nonconsensus sequence of the CTD were substituted with serine residues (Dias et al. 2015). Similar to wild-type human GFP-RPB1 (Fig. 6D), wild-type mouse GFP-RPB1 (8K) becomes ubiquitylated in response to DSBs induced by phleomycin treatment, while inhibition of DNA-PK impaired K48-linked ubiquitylation of mRPB1 (Fig. 6E). Importantly, however, we did not observe an increase in DSB-induced ubiquitylation of mutant mRPB1 (0K) (Fig. 6D,E). Reciprocal pull-downs using TUBEs confirmed that wild-type mRPB1 (8K), but not mutant mRPB1 (0K), was ubiquitylated following DSB induction (Supplemental Fig. S7I). This indicates that the ubiquitylation of RPB1 induced by DSBs occurs mainly, if not solely, on the lysines in the CTD nonconsensus sequence. Most notably, we found that wild-type and mutant mRPB1 interact equally efficiently with WWP2 (Supplemental Fig. S7J), suggesting that the eight lysine substitutions in the CTD of RPB1 do not affect its ubiquitylation by impairing the interaction with WWP2. Rather, RPB1 ubiquitylation is abrogated because WWP2's target sites for ubiquitylation are absent.

**Figure 7.**
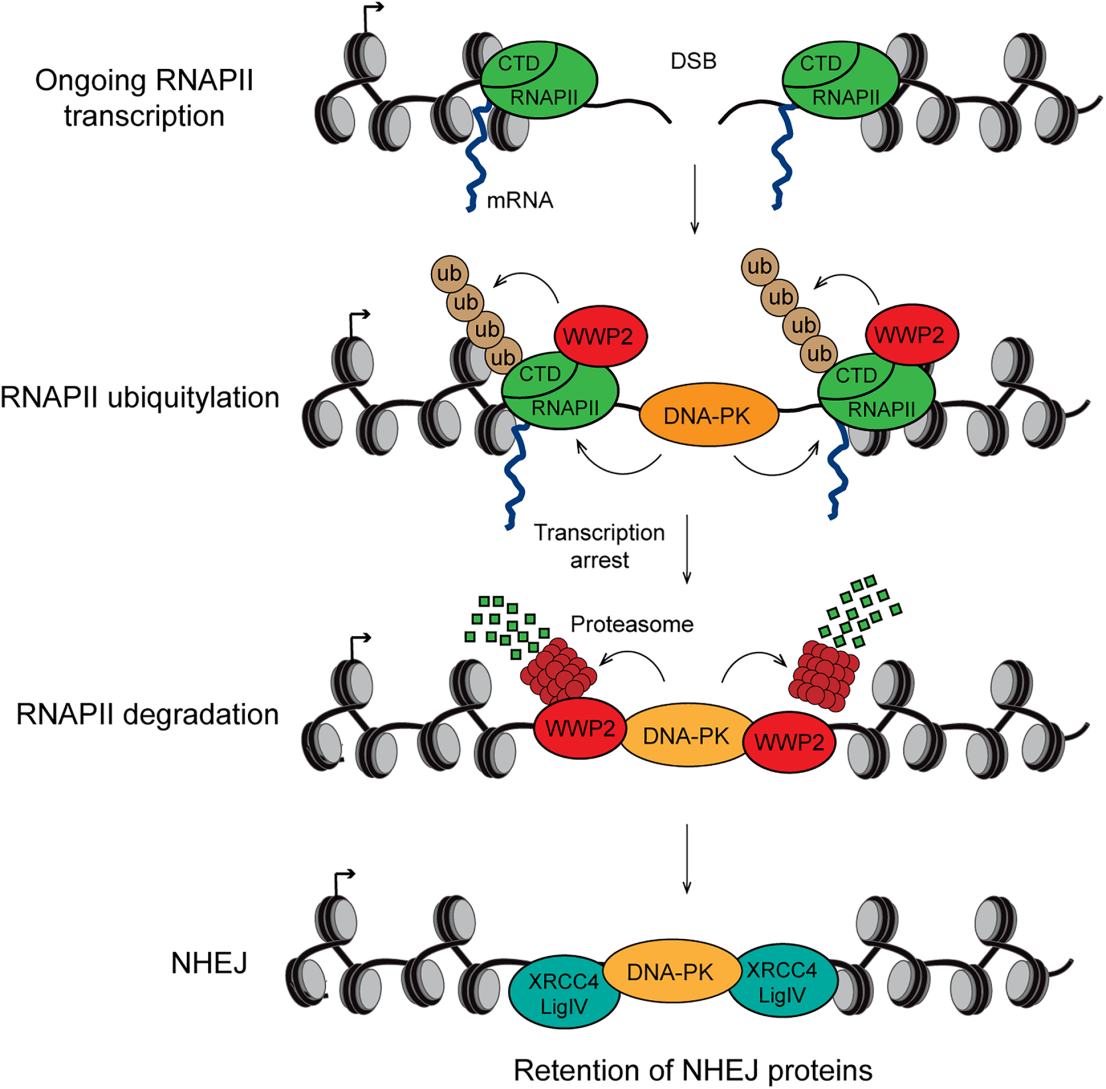
Model of how DNA-PK/WWP2-dependent transcription silencing at DSBs promotes NHEJ. DNA-PK and the HECT E3 ubiquitin ligase WWP2 are recruited to a DSB in a gene that is actively transcribed by RNAPII. DNA-PK effectuates WWP2-dependent K48-linked ubiquitylation of the CTD of RNAPII subunit RPB1 and the subsequent recruitment of the proteasome. The proteasome triggers RNAPII degradation directly on damaged chromatin, thereby silencing transcription of the broken gene. Finally, transcriptional silencing prevents the loss of DNA-PK and downstream NHEJ factors from DSBs, likely by protecting the NHEJ machinery from collision with the transcription machinery, thereby promoting efficient DSB repair via NHEJ.

To assess whether the role of WWP2 in promoting NHEJ involves its function in ubiquitylating RPB1, we monitored the accumulation of XRCC4 at DSBs exposed to UV-A laser microirradiation. We found that the accumulation of XRCC4 at sites of laser-induced DNA damage was impaired in cells expressing mutant (0K) versus wild-type (8K) GFP-RPB1 (Fig. 6F). We also examined p-DNA-PKcs (S2056) focus formation in these cells. A clear induction of focus formation of p-DNA-PKcs in IR-exposed cells expressing wild-type (8K) GFP-RPB1 (Fig. 6G) was observed. However, focus formation of p-DNA-PKcs was dramatically reduced in IR-exposed cells expressing mutant (0K) GFP-RPB1. Thus, our results suggest that DSB-induced ubiquitylation of RPB1 occurs mainly within its CTD. This further promotes DNA-PK activation and, subsequently, the retention of downstream NHEJ factors, the latter of which involves the eviction of RNAPII to prevent transcription-dependent clearance of NHEJ proteins at DSB sites (Fig. 7).

## Discussion

In this study, we provide insight into the molecular events that lead to transcription silencing induced by DSBs at RNAPII transcribed genes. The repression of transcription occurs via K48-linked ubiquitylation of the CTD of the RNAPII subunit RPB1. This process is regulated by the DNA-PK complex and its effector, the HECT E3 ubiquitin ligase WWP2. Moreover, it leads to RNAPII degradation directly on damaged chromatin through recruitment of the proteasome. Both WWP2 and the ubiquitylation of RPB1's CTD are important for the proper retention of core NHEJ factors at DSBs. We propose that removal of RNAPII from DSBs at transcribed genes protects the NHEJ machinery from collision with the transcription machinery. This in turn prevents the loss of activated DNA-PK and downstream NHEJ factors from DSBs, thereby promoting efficient DSB repair via NHEJ (Fig. 7).

### WWP2 promotes cNHEJ

E3 ubiquitin ligases can be classified into three groups: the RING ligases, the cullin-RING ligases, and the HECT ligases. Several RING ligases have been shown to play a crucial role in regulating DSB repair. For instance, RNF8 and RNF138 regulate Ku70/Ku80 ubiquitylation in G1 and S/G2, respectively (Feng and Chen 2012; Ismail et al. 2015). In addition, cullin-RING ligase activity was also shown to drive this process, although the identity of the ligase involved is unknown (Brown et al. 2015). Ubiquitylated Ku70/Ku80 is then removed from chromatin in a VCP-dependent manner and targeted for degradation by the proteasome (van den Boom et al. 2016). This allows for completion of NHEJ (Ishida et al. 2017) or activation of end resection, thereby triggering the alternative DSB repair pathway of HR (Ismail et al. 2015; van den Boom et al. 2016). The FBXW7-associated cullin-RING ligase, on the other hand, regulates the recruitment of XRCC4 to DSB sites through its K63-linked ubiquitylation (Zhang et al. 2016). This stimulates the interaction between XRCC4 and Ku70/Ku80 to promote efficient NHEJ. Finally, histones in DSB-flanking chromatin are subject to ubiquitylation. DSBs activate ATM, which leads to the recruitment of two RING ligases—RNF8 and RING1b—that monoubiquitylate H2AK119 (Shanbhag et al. 2010; Kakarougkas et al. 2014; Ui et al. 2015). This histone mark is required to silence transcription of DSB-flanking genes and is thought to promote DSB repair via cNHEJ by promoting the efficient recruitment or retention of Ku70/Ku80 at DNA breaks (Kakarougkas et al. 2014; Ui et al. 2015).

While it is evident that RING and cullin-RING ligase play crucial roles in regulating NHEJ, the role of HECT ligases in this DNA repair process remained unclear. Here, we provide several lines of evidence supporting a direct role for the HECT E3 ubiquitin ligase WWP2 in cNHEJ factors. First, we demonstrated that WWP2 is recruited to sites of DNA damage inflicted by laser microirradiation as well as to bona fide nuclease-induced DSBs. Second, the loss of WWP2 impaired the association of core NHEJ such as Ku70, Ku80, and XRCC4 as well as the activation of DNA-PK at DNA breaks. Third, the depletion of WWP2 dramatically impaired NHEJ in EJ5-GFP assays and random plasmid integration assays as well as at I-PpoI-induced DSBs in RNAPII transcribed genes. Fourth, WWP2 protected cells against IR-induced DSBs, which are predominantly repaired by NHEJ. Together, these findings suggest that the HECT E3 ubiquitin ligase WWP2 is an important player in the cNHEJ repair pathway of DSB repair.

### WWP2 targets RNAPII for cNHEJ

How does WWP2 regulate cNHEJ? Several observations suggested that WWP2 regulates this repair process by targeting RNAPII. First, we identified 11 of the 12 RNAPII subunits as WWP2-interacting proteins by pull-down coupled to MS. Importantly, the largest RNAPII subunit, RPB1, which plays a pivotal role in transcription regulation, appeared to be a strong interactor of WWP2. Second, we found that DSBs lead to a clear increase in the K48-linked ubiquitylation of RPB1 in a manner dependent on DNA-PK and its effector, WWP2. Intriguingly, this modification occurs on the lysine residues that reside in the nonconsensus sequences of the CTD, which is critical for RPB1's role in transcription regulation. Third, functional analysis of these lysines revealed that their DSB-induced ubiquitylation by WWP2 is important to promote DNA-PK activation during cNHEJ. Given that WWP2 also promotes efficient accrual of Ku70/Ku80 and XRCC4 at DNA breaks, our observations strongly suggest that WWP2 promotes NHEJ by regulating RPB1 ubiquitylation following DSB induction. However, the fact that DNA-PKcs activity is required for RBP1 ubiquitylation and its removal from damaged chromatin may indicate that WWP2 is not involved in the initial recruitment of the NHEJ machinery to DNA breaks but rather promotes its stabilization at these lesions by clearing out the RNAPII machinery. Moreover, we cannot rule out the possibility that WWP2 also ubiquitylates other components of the cNHEJ machinery to regulate DSB repair. In addition, WWP2 may also target components of DNA repair pathways other than cNHEJ, potentially broadening its regulatory function in the DNA damage response. Future work will be required to unravel how widespread WWP2's role in this response is.

### WWP2 promotes transcription silencing of broken genes

What is the role of WWP2-mediated RPB1 ubiquitylation in transcription regulation at DSBs? We found that WWP2 promotes transcriptional silencing at sites of DNA damage induced by laser microirradiation as well as at bona fide DSBs induced at RNAPII transcribed genes. Our work suggests that this process strongly depends on the WWP2-mediated ubiquitylation of RPB1. First, this posttranslational modification triggered the proteasome-dependent eviction of RNAPII from DSB sites. Second, this local RNAPII eviction led to loss of transcription. Thus, WWP2 promotes transcription silencing following DSB induction at RNAPII transcribed genes by regulating RPB1 ubiquitylation and its local eviction. However, we observed that RNAPII eviction and transcription repression were mostly delayed and not completely abrogated in the absence of WWP2, suggesting the existence of alternative mechanisms potentially involving other E3 ubiquitin ligases that may cooperate with WWP2 to promote efficient transcriptional silencing at DSBs.

### WWP2-dependent transcription silencing and cNHEJ

How does WWP2-dependent transcription silencing of broken genes affect their repair by NHEJ? It has been shown that in response to DSBs, transcriptional elongation factor ENL (MLLT1) is phosphorylated by ATM (Ui et al. 2015; Ui and Yasui 2016). This phosphorylation enhances the interaction between ENL and PRC1 and enforces accrual of PRC1 at transcription elongation sites near DSBs, leading to transcriptional repression via PRC1-mediated ubiquitylation of histone H2A. Strikingly, both ENL and PRC1 are also necessary for the accumulation of Ku70 at DSBs near active transcription sites, suggesting a functional interplay between transcription repression and cNHEJ (Ui et al. 2015; Ui and Yasui 2016). Indeed, we observed that DNA-PK and WWP2 activities are required to repress transcription elongation when DSBs arise in actively transcribed genes, thereby also preserving the association of NHEJ factors with broken ends. These findings may suggest a scenario in which transcription silencing prevents direct collision between the elongating RNAPII machinery and the NHEJ machinery at DNA breaks, thereby preventing its early loss from DNA lesions and promoting efficient cNHEJ.

### Cross-talk of DNA-PK and WWP2 during transcription silencing of broken genes

We reported previously that transcription arrest in response to DSBs in RNAPII transcribed genes is regulated by DNA-PK activity (Pankotai et al. 2012; Pankotai and Soutoglou 2013). Here we demonstrate that DNA-PK activity triggers this process by promoting (1) WWP2-dependent K48-linked ubiquitylation of RPB1, (2) recruitment of the proteasome to broken genes, and (3) proteasome-dependent release of RPB1 from broken genes. However, while DNA-PK binding is restricted to DSB ends, we found that WWP2, RBP1, and the proteasome spread across DSB-containing genes. This raises the question of how DNA-PK can trigger a WWP2- and proteasome-dependent release of RBP1 across broken genes. A possibility is that a yet-to-be-identified protein becomes phosphorylated and activated by DNA-PK and signals to WWP2 to trigger ubiquitylation and proteasome-dependent release of RPB1. Future work may therefore focus on uncovering the identity and mode of action of this protein to increase our understanding of how DNA-PK- and WWP2-dependent transcriptional silencing at broken genes is orchestrated.

### DNA-PK- and WWP2-dependent transcription silencing is unique to broken genes

DSBs that arise in a gene that is actively transcribed by RNAPII lead to a DNA-PK- and WWP2-dependent arrest of transcription elongation. This process, which remained unaffected by ATM inhibition (Pankotai et al. 2012), is mediated by the ubiquitylation and eviction of RNAPII. In contrast, DSBs generated in close proximity to a gene lead to its transient repression through ATM- or PARP-1-mediated chromatin remodeling, which induces a chromatin context that is repressive for transcription (Shanbhag et al. 2010; Kakarougkas et al. 2014; Gong et al. 2015, 2017; Ui et al. 2015; Ui and Yasui 2016; Awwad et al. 2017; Abu-Zhayia et al. 2018; Gong and Miller 2018). This process, which was not affected by DNA-PK inhibition (Shanbhag et al. 2010), relies on the recruitment and activities of PRC1 and negative transcription factor NELF, which negatively impacted phospho-RPB1 (S2 and S5) levels but not that of unmodified RPB1 (Chou et al. 2010; Shanbhag et al. 2010; Polo et al. 2012; Awwad et al. 2017). Thus, ATM- and PARP1-dependent transcription silencing, in contrast to that regulated by DNA-PK and WWP2, may not involve RNAPII eviction and relies largely on a transient arrest of elongating RNAPII induced by chromatin remodeling and negative regulators of transcription elongation. Moreover, it suggests that two distinct mechanisms exist for the silencing of transcription when DSBs occur either within or in close proximity to an actively transcribed gene, relying on DNA-PK/WWP2 and ATM/PARP, respectively. A better understanding of the context in which the DNA-PK-, ATM-, and PARP1-dependent signaling pathways are activated will help to further clarify potential cross-talk between DNA-PK-, ATM-, and PARP1-mediated silencing at DNA breaks. Moreover, transcription can also be initiated from DSB sites to produce DNA damage-induced RNAs (ddRNAs) that regulate the DNA damage response (Ohle et al. 2016; Michelini et al. 2017). It will be of interest to unravel how the interplay between DNA-PK-, ATM-, and PARP1-mediated transcription silencing and transcription of ddRNAs is orchestrated at DSBs.

## Material and methods

### Cell culture

U2OS, HeLa GFP-Ku80 (a kind gift from D. van Gent), U2OS GFP-WWP2, U2OS GFP-RPB1, and U2OS-pEP15 cells were maintained in DMEM (Dulbecco's modified Eagle's medium) supplemented with 10% FBS (fetal bovine serum) and antibiotics. NIH3T3 GFP-RPB1 cells (a kind gift from A. Pombo) were maintained in DMEM GlutaMAX-I (Gibco) and HEPES (Gibco) supplemented with 10% FBS and antibiotics. All cell lines were cultured in 5% CO_2_ at 37°C.

### Generation of stable cell lines

U2OS-pEP15 cells were generated by cotransfection of U2OS cells with pWHE1-146 and pWHE1-320-HA-ER-I-PpoI plasmids (Lemaitre et al. 2014). pWHE1-320-HA-ER-I-PpoI was generated by cloning HA-ER-PpoI, which was obtained as an EcoRI fragment from pBABE-Puro-HA-ER-I-PpoI (Pankotai et al. 2012), into EcoRI-digested PWHE1-320. pWHE1-146 allowed for expression of the reverse tetracycline-controlled transcription activator (rtTA), which, upon Dox addition, can bind the tet operator in pWHE1-320-HA-ER-I-PpoI to drive expression of HA-ER-I-PpoI. Stable clones were selected by 1000 µg/mL G418 (Sigma-Aldrich) resistance and analyzed by immunostaining of HA-I-PpoI and γH2AX after Dox and 4-hydroxytamoxifen (4-OHT) treatment.

U2OS cells stably expressing GFP-tagged WWP2 were generated by transfection of U2OS cells with pEGFP-C1-WWP2-IRES-Puro plasmid. This plasmid was generated by cloning WWP2 cDNA, which was obtained as a BglII/EcoRI fragment from pDEST-WWP2, into BglII/EcoRI-digested pEGFP-C1-IRES-Puro. Stable clones were selected by 1 µg/mL puromycin resistance and subjected to Western blot analysis for GFP-WWP2 expression.

U2OS cells stably expressing α-amanitin-resistant EYFP-tagged RPB1 were generated by transfection of U2OS cells with the pYFP-RPB1aAMR plasmid (75284 from Addgene; originally from Roger Stinger). Thirty-six hours after transfection, the cells were incubated in the presence of 2 µg/mL α-amanitin (Sigma) for 4–5 d. Individual clones were selected by 500 µg/mL G418 (Sigma-Aldrich) resistance and subjected to Western blot analysis for EYFP-RPB1 expression.

### siRNA and plasmid transfections

Cells were transfected with siRNAs (Table 1) using RNAiMAX (Invitrogen) according to the manufacturer's instructions. Typically, cells were transfected twice with siRNAs at 0 and 24 h at a concentration of 20 nM. After 24 h, the medium was replaced by DMEM GlutaMAX-I (Gibco) supplemented with 10% FBS and antibiotics, and cells were used for further experiments. Cells were transfected with plasmid DNA using Lipofectamine 2000 (Invitrogen) or JetPEI (Polyplus) according to the manufacturer's instructions. Cells were typically analyzed 24 h after transfection.

**Table 1.**
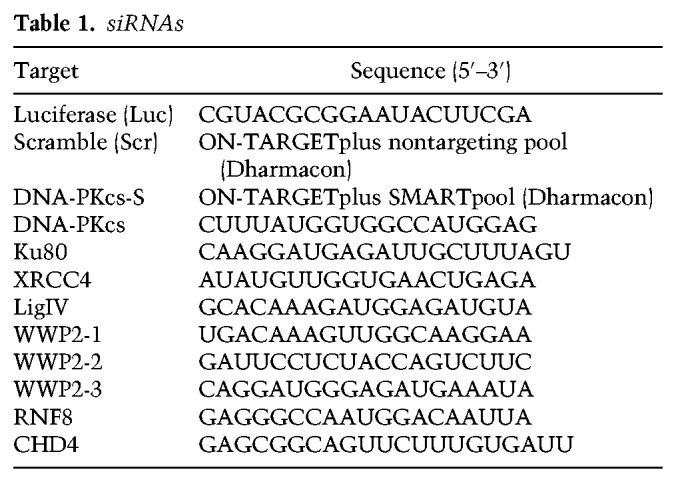
siRNAs

### Preparation of MS samples

For SILAC labeling, U2OS cells expressing WWP2-GFP or GFP-NLS were cultured for 14 d in medium containing “heavy” (H)- and “light” (L)-labeled forms of the amino acids arginine and lysine, respectively. SILAC-labeled WWP2-GFP (H) or GFP-NLS (L) cells were lysed in EBC buffer (50 mM Tris at pH 7.5, 150 mM NaCl, 0.5% NP-40, 2.5 mM MgCl_2_, protease inhibitor cocktail [Roche]) in the presence of 500 U of benzonase. Lysates were subjected to pull-down using GFP-Trap-A beads (Chromotek). The beads were subsequently washed twice with EBC-300 buffer and twice with 50 mM (NH_4_)_2_CO_3_ followed by overnight digestion using 2.5 µg of trypsin at 37°C under constant shaking. Peptides of the WWP2-GFP (H) or GFP-NLS (L) precipitates were mixed in a 1:1 ratio and desalted using a Sep-Pak tC18 cartridge by washing with 0.1% acetic acid. Finally, peptides were eluted with 0.1% acetic acid and 60% acetonitrile and lyophilized.

### MS analysis

MS was performed essentially as described previously (Schimmel et al. 2014). Samples were analyzed in technical duplicates on a Q-Exactive Orbitrap mass spectrometer (Thermo Scientific) coupled to an EASY-nanoLC 1000 system (Proxeon, Odense). Digested peptides were separated using a 13-cm fused silica capillary (ID: 75 µm, OD: 375 µm; Polymicro Technologies) packed in-house with 1.8-µm C18 beads (Reprospher; Dr. Maisch, Ammerburch-Entringen). Peptides were separated by liquid chromatography using a gradient of 2% to 95% acetonitrile with 0.1% formic acid at a flow rate of 200 nL/min for 2 h. The mass spectrometer was operated in positive-ion mode at 2.2 kV with the capillary heated to 200°C. Data-dependent acquisition mode was used to automatically switch between full-scan MS and tandem MS (MS/MS) scans, using a top 10 method. Full-scan MS spectra were obtained with a resolution of 70,000, a target value of 3 × 10^6^, and a scan range from 400 to 2000 *m/z*. Higher collisional dissociation (HCD) MS/MS was recorded with a resolution of 17,500, a target value of 1 × 10^5^, and a normalized collision energy of 25%. The precursor ion masses selected for MS/MS analysis were subsequently dynamically excluded from MS/MS analysis for 60 sec. Precursor ions with a charge state of 1 and >6 were excluded from triggering MS/MS events. Raw MS files were analyzed with the MaxQuant software suite (version 1.45.5.1; Max Planck Institute of Biochemistry). The data have been deposited to the ProteomeXchange Consortium via the PRIDE (Proteomics Identifications) partner repository with the data set identifier PXD012606.

### Chemicals

Cells were treated with phleomycin (InvivoGen) at the indicated concentrations for 1 h and collected for further analysis. Cells were treated with neocarzinostatin (Sigma-Aldrich) at a final concentration of 250 ng/mL for 15 min, washed, fixed, and harvested at the indicated time points after treatment. For multiphoton laser microirradiation, cells were exposed to DRB (Sigma-Aldrich), which was dissolved in DMSO, for 6 h at a final concentration of 100 µM. For chromatin fractionation experiments, cells were exposed to the proteasome inhibitor MG-132 (Tocris Bioscience) for 1 h at a final concentration of 20 µM, whereas for RPB1 ubiquitylation assays, cells were exposed to MG-132 (Sigma-Aldrich) for 85 min at a final concentration of 5 µM. For the analysis of p-RPB1 (S2) ubiquitylation, cells were exposed for 1 h to the broad-spectrum inhibitor of deubiquitylating enzymes PR169 (LifeSensors, SI9619), which was dissolved in DMSO and used at a final concentration of 20 μM. DNA-PKcs inhibitor (NU7026; Millipore) was dissolved in methanol and used at a final concentration of 10 µM in RPB1 ubiquitylation assays. DNA-PKcs inhibitor (NU7026; Sigma-Aldrich) was dissolved in DMSO and used at a 20 µM final concentration for ChIP and chromatin fractionation experiments.

### Generation of DSBs by IR

IR was delivered to U2OS and NIH3T3 cells by an YXlon X-ray generator machine (200 kV; 4 mA; dose rate 1 Gy/min).

### UV-A laser microirradiation

U2OS cells were grown on 18-mm coverslips and sensitized with 10 µM 5′-bromo-2-deoxyuridine (BrdU) for 24 h as described (Luijsterburg et al. 2016). For microirradiation, the cells were placed in a Chamlide TC-A live-cell imaging chamber that was mounted on the stage of a Leica DM IRBE wide-field microscope stand (Leica) integrated with a pulsed nitrogen laser (Micropoint Ablation Laser System; Andor). The pulsed nitrogen laser (16 Hz, 364 nm) was directly coupled to the epifluorescence path of the microscope and focused through a Leica 40× HCX plan apo 1.25–0.75 oil immersion objective. The growth medium was replaced by CO_2_-independent Leibovitz's L15 medium supplemented with 10% FCS and penicillin–streptomycin (Invitrogen), and cells were kept at 37°C. The laser output power was set to 72–78 to generate strictly localized subnuclear DNA damage. Following microirradiation, cells were incubated for the indicated time points at 37°C in Leibovitz's L15 and subsequently fixed with 4% formaldehyde before immunostaining. Cells were microirradiated (two iterations per pixel) within 7–10 min using Andor IQ software (Andor).

### Multiphoton laser microirradiation

U2OS and NIH3T3 cells were grown on 18-mm coverslips and placed in a Chamlide CMB magnetic chamber with CO_2_-independent Leibovitz's L15 medium supplemented with 10% FCS and penicillin–streptomycin (Invitrogen). Laser microirradiation was carried out on a Leica SP5 confocal microscope equipped with an environmental chamber set to 37°C. DSB-containing tracks (1.5-µm width) were generated with a Mira mode locked titanium–sapphire (Ti:sapphire) laser (*l* = 800 nm; pulse length = 200 fs; repetition rate = 76 MHz; output power = 80 mW) using a UV-transmitting 63× HCX plan apo 1.4 NA oil immersion objective (Leica). Confocal images were recorded before and after laser irradiation at 5- or 10-sec time intervals over a period of 3–5 min.

### EJ5-GFP reporter assay

HEK293 cell lines containing a stably integrated copy of the EJ5-GFP reporter were used to measure the repair of I-SceI-induced DSBs by NHEJ (Bennardo et al. 2008). Briefly, 48 h after siRNA transfection, cells were cotransfected with a mCherry expression vector and the I-SceI expression vector pCBASce. Forty-eight hours later, the percentage of GFP-positive cells among mCherry-positive cells was determined by FACS on a BD LSRII flow cytometer (BD Bioscience) using FACSDiva software version 5.0.3. Quantifications were performed using WinMDI 2.9 (freeware), FACSDiva (BD Biosciences), or FlowJo software (Flowing Software 5.2.1.).

### Random plasmid integration assay

U2OS cells were seeded (day 1) and transfected with siRNAs the following day (day 2). At the end of day 2, the cells were transfected with 2 µg of gel-purified BamHI–EcoRI-linearized pEGFP-C1 plasmid. The cells were subsequently transfected twice with siRNAs at 24 and 36 h after the first transfection (day 3 and day 4, respectively). On day 5, cells were collected, counted, seeded, and grown in medium without or with 0.5 mg/mL G418. The transfection efficiency was determined on the same day by FACS analysis. The cells were incubated at 37°C to allow colony formation, and the medium was refreshed on days 8 and 12. On day 15, the cells were washed with 0.9% NaCl and stained with methylene blue. Colonies of >50 cells were scored. Random plasmid integration efficiency was scored as the number of G418-resistant colonies normalized by the plating efficiency, which was determined by the number of colonies formed on plates without G418.

### Immunofluorescence (IF)

U2OS and NIH3T3 cells were grown on glass coverslips in a 12-well plate, rinsed three times with PBS (phosphate-buffered saline), and fixed on the coverslips with 4% formaldehyde for 12 min. Next, the cells were rinsed three times with PBS, permeabilized with 0.5% Triton for 5 min, and then rinsed again three times with PBS. Subsequently, 3% PBS-BSA (bovine serum albumin) was added to the cells for 30 min, after which the solution was removed, and a solution of 3% PBS-BSA with primary antibodies was added (Table 2). After incubation for 2 h or overnight, the cover slips were washed four times with 3% PBS-BSA, and a solution of 3% PBS-BSA with secondary antibodies was added. Next, coverslips were placed in the dark. After 2 h, cells were rinsed three times with PBS, and a solution of PBS-DAPI (4′,6-diamidino-2-phenylindole) was added to the cells. Ten minutes later, the solution was removed by washing three times with PBS. Next, a drop of Aqua-Polymont was placed on a microscope slide, and the coverslip was placed on top of it. Images were taken with a Zeiss AxioImager D2 wide-field fluorescence microscope equipped with a 40.63 and 100× plan apo (1.4 NA) oil immersion objective, an HXP 120 metal-halide lamp for excitation, and Zen 2012 software. ImageJ software was used for image analysis.

**Table 2.**
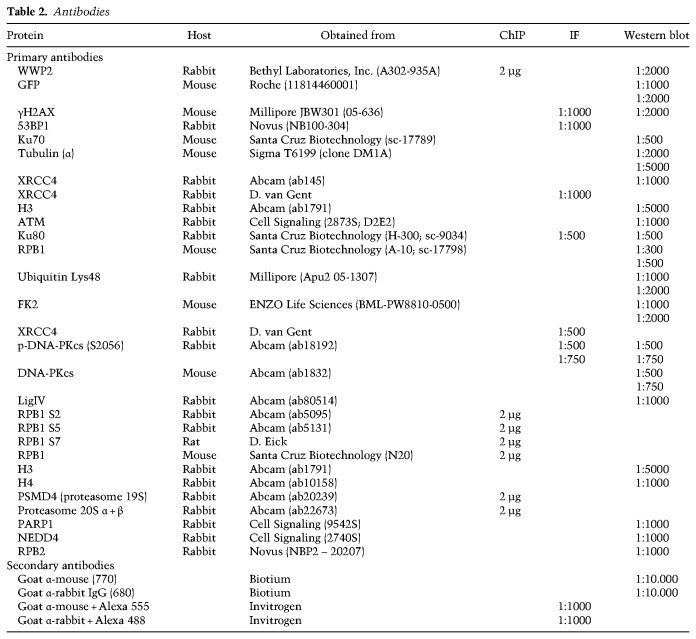
Antibodies

### ChIP after I-PpoI-induced DSBs

U2OS cells stably expressing HA-ER-I-PpoI (U2OS-pEP15) were transfected with siRNAs (Table 1) at a concentration of 25 nM using RNAiMAX (Invitrogen) according to the manufacturer's instructions. After 24 h, the medium was replaced by DMEM GlutaMAX-I (Gibco) supplemented with 10% FBS and antibiotics, and cells were used for further experiments. After 36 h of transfection, cells were treated with 1 µg/mL Dox (Sigma-Aldrich) for 12 h, and then 4-OHT (Sigma-Aldrich) was added at a 2 µM final concentration to induce nuclear translocation of HA-ER-I-PpoI. Cells were harvested at different time points up to 6 h after 4-OHT addition. DNA-PK inhibitor (NU7026; Sigma and Millipore) was added 1 h prior the addition of 4-OHT. ChIP was performed as described with few modifications (Pankotai et al. 2012). Briefly, one 150-mm dish with 50% confluent cells was used for each time point. The cells were cross-linked for 10 min in 0.75% (v/v) paraformaldehyde (Electron Microscopy Sciences) and then sonicated in sonication buffer (50 mM HEPES at pH 8, 140 mM NaCl, 1 mM EDTA, 1% [v/v] Triton X-100, 1% [v/v] SDS, protease inhibitor cocktail [Roche]). Twenty-five milligrams of chromatin was diluted 10 times with RIPA buffer (50 mM Tris-HCl at pH 8, 150 mM NaCl, 1 mM EDTA, 1% [v/v] Triton X-100, 0.1% [w/v] Na-deoxycholate, 0/1% [v/v] SDS) and subjected to immunoprecipitation using 2 µg of antibody (Table 2) and 30 µL of (∼10^7^) Dynabeads M-280 (Invitrogen). The beads were washed once for 10 min with low-salt buffer (20 mM Tris-HCl at pH 8, 150 mM NaCl, 2 mM EDTA, 1% [v/v] Triton X-100, 0.1% [v/v] SDS), once for 10 min with high-salt buffer (20 mM Tris-HCl at pH 8, 500 mM NaCl, 2 mM EDTA, 1% [v/v] Triton X-100, 0.1% [v/v] SDS), once for 10 min with wash buffer (10 mM Tris-HCl at pH 8, 250 mM LiCl, 1 mM EDTA, 1% [v/v] NP-40, 1% [w/v] Na-deoxycholate), and twice for 10 min each with TE buffer. The elution was done twice for 15 min at 65°C. Cross-links were reversed by incubation for 6 h at 65°C. The DNA was purified after Proteinase K and RNase A treatment using phenol-chloroform extraction and resuspended in 50 µL of TE buffer. The enrichment in each experiment was calculated using the formula (immunoprecipitation sample − IgG control)/input. Each value represents a relative DNA concentration calculated by the Ct values of samples with known DNA concentrations, which were used to generate a standard curve for absolute linear quantification.

### RT-qPCR-based mRNA analysis

Total cellular RNA was purified from U2OS HA-ER-I-PpoI cells using the RNeasy total RNA purification kit (Qiagen) according to the manufacturer's instructions. cDNA was synthesized with an RT-PCR kit (Qiagen) according to the manufacturer's instructions in a final volume of 10 μL. RT-qPCR analysis was performed using 10 ng of total RNA as a template and 1 pmol of each primer (Table 3). Reactions were carried out using a Roche LightCycler 480 II system for 50 cycles. The purity of the PCR products was determined by melting curve analysis. Each value represents a relative DNA concentration calculated by the Ct values of samples with known DNA concentrations, which were used to generate a standard curve for absolute linear quantification. For each condition, mRNA values were determined, and each sample was normalized to cyclophilin B (PPIB) mRNA levels.

**Table 3.**
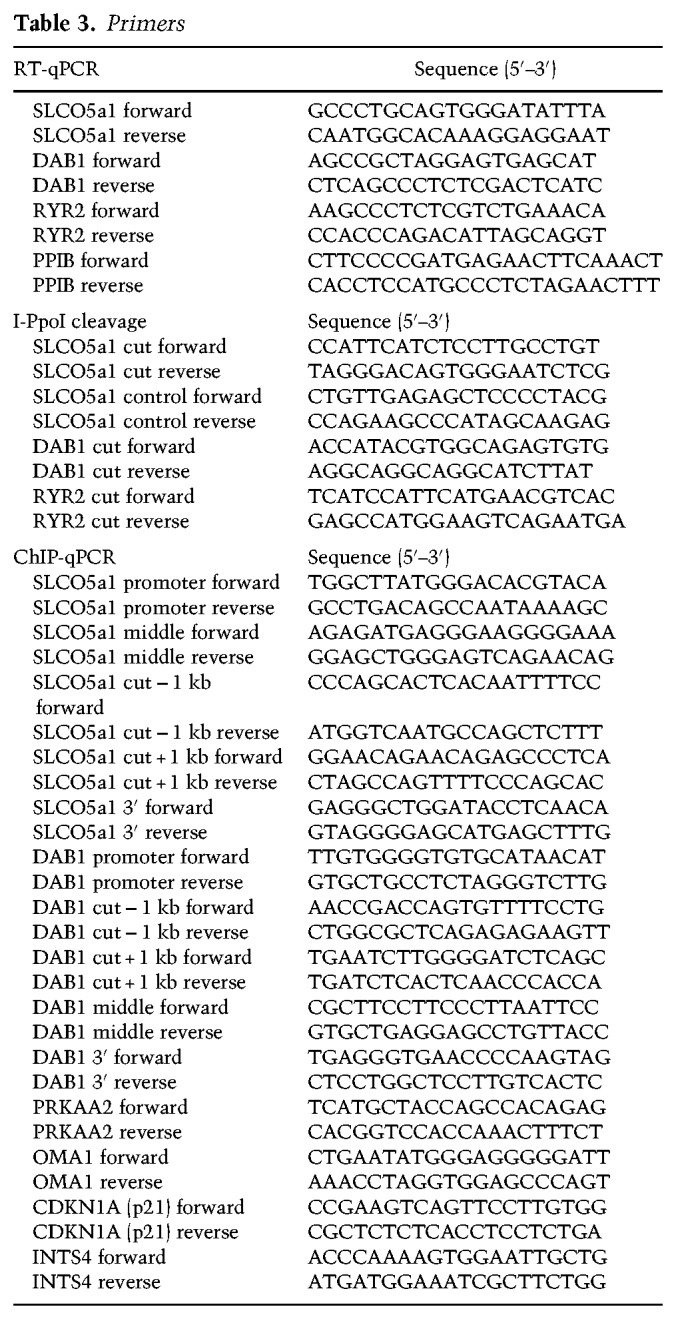
Primers

### qPCR-based analysis of I-PpoI-induced DSBs

Genomic DNA was purified from U2OS HA-ER-I-PpoI cells using a DNeasy blood and tissue purification kit (Qiagen) according to the manufacturer's instructions. qPCR analysis was performed in a final volume of 10 μL using 10 ng of genomic DNA as a template and 1 pmol of each primer (Table 3). Reactions were carried out using a Roche LightCycler 480 II system for 50 cycles. The purity of the PCR products was determined by melting curve analysis. Cutting efficiency was calculated by measuring the uncut DNA fraction by qPCR using primers spanning the I-PpoI recognition site and comparing that with the amplification of a part of the gene lacking such a site.

### Monitoring nascent transcription at DNA damage sites

Nascent transcription was monitored by 5-EU labeling of nascent RNA with 1 mM 5-EU, which was added 5 min after UV-A or multiphoton laser microirradiation. 5-EU incorporation was determined 1 h later by using a Click-it RNA imaging kit (Invitrogen) as described previously (Gong et al. 2015).

### TUBE assay

TUBE assays were performed according to the manufacturer's instructions (LifeSensors, UM401 and UM402), except that cells were collected from plates prior to cell lysis, and cell lysates were treated with 0.25 U/µL benzonase for 1 h at 4°C under constant mixing.

### GFP pull-down under denaturing conditions

For each immunoprecipitation, 2 × 10^6^ to 15 × 10^6^ cells were collected and resuspended in 200 µL of lysis buffer (20 mM Tris/CI at pH 7.5, 50 mM NaCI, 0.5% IGEPAL, 1% sodium deoxycholat, 1% SDS, 5 mM MgCl_2_, protease inhibitor cocktail [Roche]). Next, 800 µL of wash buffer was added (20 mM Tris at pH 7.5, 50 mM NaCI, 0.5% IGEPAL, 0.5% sodium deoxycholate, 0.5% SDS, Protease inhibitor cocktail tablets) with 0.25 U/µL benzonase (final concentration), and samples were incubated for 60 min at room temperature. Cell lysates were centrifuged at 15,000*g* for 10 min at room temperature. Fifty microliters of cell lysate (input) was collected in a separate tube and mixed with 2× Laemmli buffer. The remaining cell lysate was added to 25 µL of GFP-Trap-A beads (Chromotek) and incubated under constant mixing conditions for 2 h at room temperature. Beads were washed six times with 1000 µL of wash buffer. Twenty-five microliters of 2× Laemmli buffer was added to the beads, and samples were boiled for 10 min. After brief centrifugation at full speed, samples were separated from the beads and subjected to Western blot analysis.

### GFP pull-down under physiological conditions

For each immunoprecipitation reaction, a cell pellet (2 × 10^6^ to 15 × 10^6^ cells) was resuspended in 1000 µL of EBC buffer (50 mM Tris at pH 7.5, 150 mM NaCl, 0.5% NP-40, 2.5 mM MgCl_2_, Protease inhibitor cocktail tablets), and 0.25 U/µL benzonase was added. Samples were incubated for 60 min at 4°C under constant mixing. The cell lysate was centrifuged at 15,000*g* for 10 min at room temperature. Fifty microliters of cell lysate (input) was collected in a separate tube and mixed with 2× Laemmli buffer. Twenty-five microliters of prewashed GFP-Trap-A beads (Chromotek) was added to the remaining cell lysate and incubated under constant mixing conditions for 2 h or overnight at 4°C and then washed five times with the EBC buffer with the NaCl concentration adjusted to 250 mM. Twenty-five microliters of 2× Laemmli buffer was added to the beads and boiled for 10 min. After brief centrifugation at full speed, samples were separated from the beads and subjected to Western-blot analysis.

### Western blot analysis

Cells were lysed in 2× Laemmli buffer, and proteins were separated by SDS-PAGE for 3–4 h at 75 V using 4%–12% precast polyacrylamide gels (Bio-Rad) and 20× MOPS running buffer (Invitrogen). Next, proteins were transferred onto nitrocellulose membranes (Millipore) for 2 h at 52 V. The membrane was probed with primary antibodies for 2 h or overnight and washed three times with 0.1% PBS-Tween 20 followed by incubation with secondary antibodies for 2 h (Table 3). Membranes were scanned and analyzed using a Licor Odyssey scanner (LI-COR Biosciences).

### Chromatin fractionation

Cells were collected in PBS complemented with PIC (Roche). The pellets were collected by centrifugation and then resuspended in hypotonic buffer (10 mM Tris-HCl at pH 8.0, 1.5 mM MgCl_2_, 10 mM KCl, 1× PIC), and homogenized with a loose dounce homogenizer. Next, cells were supplemented with sucrose buffer (20 mM Tris-HCl at pH 8.0, 15 mM KCl, 60 mM NaCl, 0.34 mM sucrose [Sigma-Aldrich], 0.15 mM spermine [Sigma-Aldrich], 0.5 mM spermidine [Sigma-Aldrich], 1× PIC). The supernatant or cytoplamic extract was collected. The pellet was resuspended in sucrose buffer supplemented with high-salt buffer (20 mM Tris-HCl at pH 8.0, 25% glycerol [Fermentas], 1.5 mM MgCl_2_, 0.2 mM EDTA, 900 mM NaCl, 1× PIC) and incubated for 30 min on ice. The supernatant or nuclear extract was collected. The pellet was resuspended in sucrose buffer, sonicated with Diagenode Bioruptor (Diagenode), and used as the chromatin fraction.

### Chromatin-binding assay

The chromatin-binding assay after phleomycin treatment was based on a previously published protocol (Britton et al. 2013) and used with modifications. Briefly, 150,000 U2OS cells were grown per 6-cm dish for 24 h and then transfected with siRNAs. Next, the cells were treated with 500 µM phleomycin for 1 h, washed three times with PBS, and incubated in extraction buffer (RNase A, 0.7% Triton X-100 in CSK buffer. RNase A was added just prior to use to prevent inactivation by Triton. After 3 min of incubation, samples were taken for the chromatin-unbound fraction and mixed with the same amount of 2× Laemmli buffer. Cells were subjected to a second extraction, washed with PBS, lysed, and incubated in Laemmli buffer with benzonase for 15 min to obtain the chromatin-bound fraction. Samples were heated for 7 min at 90°C and subjected to Western blot analysis.

### Clonogenic survival assay

Cells were transfected with siRNA (see above), seeded in 10-cm dishes (2000, 4000, or 6000 cells per plate) and exposed to different doses of IR. Eight days later, the cells were washed with 0.9% NaCl and stained with 2.5 g/L methylene blue in 5% ethanol (Sigma-Aldrich). Colonies consisting of >50 cells were counted as positive.

## Supplementary Material

Supplemental Material

## References

[GAD321943CARC1] Abu-Zhayia ER, Awwad SW, Ben-Oz BM, Khoury-Haddad H, Ayoub N. 2018 CDYL1 fosters double-strand break-induced transcription silencing and promotes homology-directed repair. J Mol Cell Biol 10: 341–357. 10.1093/jmcb/mjx050 29177481

[GAD321943CARC2] Agarwal P, Jackson SP. 2016 G9a inhibition potentiates the anti-tumour activity of DNA double-strand break inducing agents by impairing DNA repair independent of p53 status. Cancer Lett 380: 467–475. 10.1016/j.canlet.2016.07.009 27431310 PMC5011428

[GAD321943CARC3] Awwad SW, Abu-Zhayia ER, Guttmann-Raviv N, Ayoub N. 2017 NELF-E is recruited to DNA double-strand break sites to promote transcriptional repression and repair. EMBO Rep 18: 745–764. 10.15252/embr.201643191 28336775 PMC5412775

[GAD321943CARC4] Bennardo N, Cheng A, Huang N, Stark JM. 2008 Alternative-NHEJ is a mechanistically distinct pathway of mammalian chromosome break repair. PLoS Genet 4: e1000110 10.1371/journal.pgen.1000110 18584027 PMC2430616

[GAD321943CARC5] Blackford AN, Jackson SP. 2017 ATM, ATR, and DNA-PK: the trinity at the heart of the DNA damage response. Mol Cell 66: 801–817. 10.1016/j.molcel.2017.05.015 28622525

[GAD321943CARC6] Bregman DB, Halaban R, van Gool AJ, Henning KA, Friedberg EC, Warren SL. 1996 UV-induced ubiquitination of RNA polymerase II: a novel modification deficient in Cockayne syndrome cells. Proc Natl Acad Sci 93: 11586–11590. 10.1073/pnas.93.21.11586 8876179 PMC38101

[GAD321943CARC07] Britton S, Coates J, Jackson SP. 2013 A new method for high-resolution imaging of Ku foci to decipher mechanisms of DNA double-strand break repair. J Cell Biol 202: 579–595. 10.1083/jcb.201303073 23897892 PMC3734090

[GAD321943CARC7] Brown JS, Lukashchuk N, Sczaniecka-Clift M, Britton S, le Sage C, Calsou P, Beli P, Galanty Y, Jackson SP. 2015 Neddylation promotes ubiquitylation and release of Ku from DNA-damage sites. Cell Rep 11: 704–714. 10.1016/j.celrep.2015.03.058 25921528 PMC4431666

[GAD321943CARC8] Butler LR, Densham RM, Jia J, Garvin AJ, Stone HR, Shah V, Weekes D, Festy F, Beesley J, Morris JR. 2012 The proteasomal de-ubiquitinating enzyme POH1 promotes the double-strand DNA break response. EMBO J 31: 3918–3934. 10.1038/emboj.2012.232 22909820 PMC3463844

[GAD321943CARC9] Chang HHY, Pannunzio NR, Adachi N, Lieber MR. 2017 Non-homologous DNA end joining and alternative pathways to double-strand break repair. Nat Rev Mol Cell Biol 18: 495–506. 10.1038/nrm.2017.48 28512351 PMC7062608

[GAD321943CARC10] Chou DM, Adamson B, Dephoure NE, Tan X, Nottke AC, Hurov KE, Gygi SP, Colaiacovo MP, Elledge SJ. 2010 A chromatin localization screen reveals poly (ADP ribose)-regulated recruitment of the repressive polycomb and NuRD complexes to sites of DNA damage. Proc Natl Acad Sci 107: 18475–18480. 10.1073/pnas.1012946107 20937877 PMC2972950

[GAD321943CARC11] Dantuma NP, van Attikum H. 2016 Spatiotemporal regulation of posttranslational modifications in the DNA damage response. EMBO J 35: 6–23. 10.15252/embj.201592595 26628622 PMC4717999

[GAD321943CARC12] Darzacq X, Shav-Tal Y, de Turris V, Brody Y, Shenoy SM, Phair RD, Singer RH. 2007 In vivo dynamics of RNA polymerase II transcription. Nat Struct Mol Biol 14: 796–806. 10.1038/nsmb1280 17676063 PMC4942130

[GAD321943CARC13] Deriano L, Roth DB. 2013 Modernizing the nonhomologous end-joining repertoire: alternative and classical NHEJ share the stage. Annu Rev Genet 47: 433–455. 10.1146/annurev-genet-110711-155540 24050180

[GAD321943CARC14] Dias JD, Rito T, Torlai Triglia E, Kukalev A, Ferrai C, Chotalia M, Brookes E, Kimura H, Pombo A. 2015 Methylation of RNA polymerase II non-consensus lysine residues marks early transcription in mammalian cells. Elife 4: e11215 10.7554/eLife.11215 26687004 PMC4758952

[GAD321943CARC15] Epshtein V, Nudler E. 2003 Cooperation between RNA polymerase molecules in transcription elongation. Science 300: 801–805. 10.1126/science.1083219 12730602

[GAD321943CARC16] Fell VL, Schild-Poulter C. 2015 The Ku heterodimer: function in DNA repair and beyond. Mutat Res Rev Mutat Res 763: 15–29. 10.1016/j.mrrev.2014.06.002 25795113

[GAD321943CARC17] Feng L, Chen J. 2012 The E3 ligase RNF8 regulates KU80 removal and NHEJ repair. Nat Struct Mol Biol 19: 201–206. 10.1038/nsmb.2211 22266820 PMC3888515

[GAD321943CARC18] Galanty Y, Belotserkovskaya R, Coates J, Polo S, Miller KM, Jackson SP. 2009 Mammalian SUMO E3-ligases PIAS1 and PIAS4 promote responses to DNA double-strand breaks. Nature 462: 935–939. 10.1038/nature08657 20016603 PMC2904806

[GAD321943CARC19] Galanty Y, Belotserkovskaya R, Coates J, Jackson SP. 2012 RNF4, a SUMO-targeted ubiquitin E3 ligase, promotes DNA double-strand break repair. Genes Dev 26: 1179–1195. 10.1101/gad.188284.112 22661229 PMC3371407

[GAD321943CARC20] Gong F, Miller KM. 2018 Double duty: ZMYND8 in the DNA damage response and cancer. Cell Cycle 17: 414–420. 10.1080/15384101.2017.1376150 29393731 PMC5927707

[GAD321943CARC21] Gong F, Chiu LY, Cox B, Aymard F, Clouaire T, Leung JW, Cammarata M, Perez M, Agarwal P, Brodbelt JS, 2015 Screen identifies bromodomain protein ZMYND8 in chromatin recognition of transcription-associated DNA damage that promotes homologous recombination. Genes Dev 29: 197–211. 10.1101/gad.252189.114 25593309 PMC4298138

[GAD321943CARC22] Gong F, Clouaire T, Aguirrebengoa M, Legube G, Miller KM. 2017 Histone demethylase KDM5A regulates the ZMYND8– NuRD chromatin remodeler to promote DNA repair. J Cell Biol 216: 1959–1974. 10.1083/jcb.201611135 28572115 PMC5496618

[GAD321943CARC23] Goodarzi AA, Noon AT, Deckbar D, Ziv Y, Shiloh Y, Löbrich M, Jeggo PA. 2008 ATM signaling facilitates repair of DNA double-strand breaks associated with heterochromatin. Mol Cell 31: 167–177. 10.1016/j.molcel.2008.05.017 18657500

[GAD321943CARC24] Hsin JP, Manley JL. 2012 The RNA polymerase II CTD coordinates transcription and RNA processing. Genes Dev 26: 2119–2137. 10.1101/gad.200303.112 23028141 PMC3465734

[GAD321943CARC25] Ishida N, Nakagawa T, Iemura SI, Yasui A, Shima H, Katoh Y, Nagasawa Y, Natsume T, Igarashi K, Nakayama K. 2017 Ubiquitylation of Ku80 by RNF126 promotes completion of nonhomologous end joining-mediated DNA repair. Mol Cell Biol 37: e00347-16 10.1128/MCB.00347-16 27895153 PMC5288581

[GAD321943CARC26] Ismail IH, Gagné JP, Genois MM, Strickfaden H, McDonald D, Xu Z, Poirier GG, Masson JY, Hendzel MJ. 2015 The RNF138 E3 ligase displaces Ku to promote DNA end resection and regulate DNA repair pathway choice. Nat Cell Biol 17: 1446–1457. 10.1038/ncb3259 26502055

[GAD321943CARC27] Jeronimo C, Collin P, Robert F. 2016 The RNA polymerase II CTD: the increasing complexity of a low-complexity protein domain. J Mol Biol 428: 2607–2622. 10.1016/j.jmb.2016.02.006 26876604

[GAD321943CARC28] Kakarougkas A, Ismail A, Chambers AL, Riballo E, Herbert AD, Kunzel J, Lobrich M, Jeggo PA, Downs JA. 2014 Requirement for PBAF in transcriptional repression and repair at DNA breaks in actively transcribed regions of chromatin. Mol Cell 55: 723–732. 10.1016/j.molcel.2014.06.028 25066234 PMC4157577

[GAD321943CARC29] Kochan JA, Desclos ECB, Bosch R, Meister L, Vriend LEM, van Attikum H, Krawczyk PM. 2017 Meta-analysis of DNA double-strand break response kinetics. Nucleic Acids Res 45: 12625–12637. 10.1093/nar/gkx1128 29182755 PMC5728399

[GAD321943CARC30] Krogan NJ, Lam MH, Fillingham J, Keogh MC, Gebbia M, Li J, Datta N, Cagney G, Buratowski S, Emili A, 2004 Proteasome involvement in the repair of DNA double-strand breaks. Mol Cell 16: 1027–1034. 10.1016/j.molcel.2004.11.033 15610744

[GAD321943CARC31] Lemaitre C, Grabarz A, Tsouroula K, Andronov L, Furst A, Pankotai T, Heyer V, Rogier M, Attwood KM, Kessler P, 2014 Nuclear position dictates DNA repair pathway choice. Genes Dev 28: 2450–2463. 10.1101/gad.248369.114 25366693 PMC4233239

[GAD321943CARC32] Li H, Zhang Z, Wang B, Zhang J, Zhao Y, Jin Y. 2007 Wwp2-mediated ubiquitination of the RNA polymerase II large subunit in mouse embryonic pluripotent stem cells. Mol Cell Biol 27: 5296–5305. 10.1128/MCB.01667-06 17526739 PMC1952083

[GAD321943CARC33] Li J, Liu Y, Rhee HS, Ghosh SK, Bai L, Pugh BF, Gilmour DS. 2013 Kinetic competition between elongation rate and binding of NELF controls promoter-proximal pausing. Mol Cell 50: 711–722. 10.1016/j.molcel.2013.05.016 23746353 PMC3695833

[GAD321943CARC34] Luijsterburg MS, de Krijger I, Wiegant WW, Shah RG, Smeenk G, de Groot AJL, Pines A, Vertegaal ACO, Jacobs JJL, Shah GM, 2016 PARP1 links CHD2-mediated chromatin expansion and H3.3 deposition to DNA repair by non-homologous end-joining. Mol Cell 61: 547–562. 10.1016/j.molcel.2016.01.019 26895424 PMC4769320

[GAD321943CARC35] Marcucci R, Brindle J, Paro S, Casadio A, Hempel S, Morrice N, Bisso A, Keegan LP, Del Sal G, O'Connell MA. 2011 Pin1 and WWP2 regulate GluR2 Q/R site RNA editing by ADAR2 with opposing effects. EMBO J 30: 4211–4222. 10.1038/emboj.2011.303 21847096 PMC3199391

[GAD321943CARC36] Marnef A, Cohen S, Legube G. 2017 Transcription-coupled DNA double-strand break repair: active genes need special care. J Mol Biol 429: 1277–1288. 10.1016/j.jmb.2017.03.024 28363678

[GAD321943CARC37] Mehta A, Haber JE. 2014 Sources of DNA double-strand breaks and models of recombinational DNA repair. Cold Spring Harb Perspect Biol 6: a016428 10.1101/cshperspect.a016428 25104768 PMC4142968

[GAD321943CARC38] Michelini F, Pitchiaya S, Vitelli V, Sharma S, Gioia U, Pessina F, Cabrini M, Wang Y, Capozzo I, Iannelli F, 2017 Damage-induced lncRNAs control the DNA damage response through interaction with DDRNAs at individual double-strand breaks. Nat Cell Biol 19: 1400–1411. 10.1038/ncb3643 29180822 PMC5714282

[GAD321943CARC39] Ohle C, Tesorero R, Schermann G, Dobrev N, Sinning I, Fischer T. 2016 Transient RNA–DNA hybrids are required for efficient double-strand break repair. Cell 167: 1001–1013.e7. 10.1016/j.cell.2016.10.001 27881299

[GAD321943CARC40] Pankotai T, Soutoglou E. 2013 Double strand breaks: hurdles for RNA polymerase II transcription? Transcription 4: 34–38. 10.4161/trns.22879 23340208 PMC3644041

[GAD321943CARC41] Pankotai T, Bonhomme C, Chen D, Soutoglou E. 2012 DNAPKcs-dependent arrest of RNA polymerase II transcription in the presence of DNA breaks. Nat Struct Mol Biol 19: 276–282. 10.1038/nsmb.2224 22343725

[GAD321943CARC42] Pannunzio NR, Watanabe G, Lieber MR. 2018 Nonhomologous DNA end-joining for repair of DNA double-strand breaks. J Biol Chem 293: 10512–10523. 10.1074/jbc.TM117.000374 29247009 PMC6036208

[GAD321943CARC43] Paul A, Wang B. 2017 RNF8- and Ube2S-dependent ubiquitin lysine 11-linkage modification in response to DNA damage. Mol Cell 66: 458–472.e5. 10.1016/j.molcel.2017.04.013 28525740 PMC5642944

[GAD321943CARC44] Polo SE, Blackford AN, Chapman JR, Baskcomb L, Gravel S, Rusch A, Thomas A, Blundred R, Smith P, Kzhyshkowska J, 2012 Regulation of DNA-end resection by hnRNPU-like proteins promotes DNA double-strand break signaling and repair. Mol Cell 45: 505–516. 10.1016/j.molcel.2011.12.035 22365830 PMC3550743

[GAD321943CARC45] Ratner JN, Balasubramanian B, Corden J, Warren SL, Bregman DB. 1998 Ultraviolet radiation-induced ubiquitination and proteasomal degradation of the large subunit of RNA polymerase II. Implications for transcription-coupled DNA repair. J Biol Chem 273: 5184–5189. 10.1074/jbc.273.9.5184 9478972

[GAD321943CARC46] Ray Chaudhuri A, Nussenzweig A. 2017 The multifaceted roles of PARP1 in DNA repair and chromatin remodelling. Nat Rev Mol Cell Biol 18: 610–621. 10.1038/nrm.2017.53 28676700 PMC6591728

[GAD321943CARC47] Scheffner M, Kumar S. 2014 Mammalian HECT ubiquitin–protein ligases: biological and pathophysiological aspects. Biochim Biophys Acta 1843: 61–74. 10.1016/j.bbamcr.2013.03.024 23545411

[GAD321943CARC048] Schimmel J, Eifler K, Sigurðsson JO, Cuijpers SA, Hendriks IA, Verlaan-de Vries M, Kelstrup CD, Francavilla C, Medema RH, Olsen JV, 2014 Uncovering SUMOylation dynamics during cell-cycle progression reveals FoxM1 as a key mitotic SUMO target protein. Mol Cell 53: 1053–1066. 10.1016/j.molcel.2014.02.001 24582501

[GAD321943CARC48] Shanbhag NM, Rafalska-Metcalf IU, Balane-Bolivar C, Janicki SM, Greenberg RA. 2010 ATM-dependent chromatin changes silence transcription in cis to DNA double-strand breaks. Cell 141: 970–981. 10.1016/j.cell.2010.04.038 20550933 PMC2920610

[GAD321943CARC49] Somesh BP, Reid J, Liu WF, Søgaard TM, Erdjument-Bromage H, Tempst P, Svejstrup JQ. 2005 Multiple mechanisms confining RNA polymerase II ubiquitylation to polymerases undergoing transcriptional arrest. Cell 121: 913–923. 10.1016/j.cell.2005.04.010 15960978

[GAD321943CARC50] Somesh BP, Sigurdsson S, Saeki H, Erdjument-Bromage H, Tempst P, Svejstrup JQ. 2007 Communication between distant sites in RNA polymerase II through ubiquitylation factors and the polymerase CTD. Cell 129: 57–68. 10.1016/j.cell.2007.01.046 17418786

[GAD321943CARC51] Sordet O, Larochelle S, Nicolas E, Stevens EV, Zhang C, Shokat KM, Fisher RP, Pommier Y. 2008 Hyperphosphorylation of RNA polymerase II in response to topoisomerase I cleavage complexes and its association with transcription- and BRCA1-dependent degradation of topoisomerase I. J Mol Biol 381: 540–549. 10.1016/j.jmb.2008.06.028 18588899 PMC2754794

[GAD321943CARC52] Spruijt CG, Luijsterburg MS, Menafra R, Lindeboom RG, Jansen PW, Edupuganti RR, Baltissen MP, Wiegant WW, Voelker-Albert MC, Matarese F, 2016 ZMYND8 co-localizes with NuRD on target genes and regulates poly(ADP-ribose)-dependent recruitment of GATAD2A/NuRD to sites of DNA damage. Cell Rep 17: 783–798. 10.1016/j.celrep.2016.09.037 27732854

[GAD321943CARC53] Ui A, Yasui A. 2016 Collaboration of MLLT1/ENL, Polycomb and ATM for transcription and genome integrity. Nucleus 7: 138–145. 10.1080/19491034.2016.1177681 27310306 PMC4916901

[GAD321943CARC54] Ui A, Nagaura Y, Yasui A. 2015 Transcriptional elongation factor ENL phosphorylated by ATM recruits polycomb and switches off transcription for DSB repair. Mol Cell 58: 468–482. 10.1016/j.molcel.2015.03.023 25921070

[GAD321943CARC55] van den Boom J, Wolf M, Weimann L, Schulze N, Li F, Kaschani F, Riemer A, Zierhut C, Kaiser M, Iliakis G, 2016 VCP/p97 extracts sterically trapped Ku70/80 rings from DNA in double-strand break repair. Mol Cell 64: 189–198. 10.1016/j.molcel.2016.08.037 27716483 PMC5161236

[GAD321943CARC56] van Haaften G, Romeijn R, Pothof J, Koole W, Mullenders LH, Pastink A, Plasterk RH, Tijsterman M. 2006 Identification of conserved pathways of DNA-damage response and radiation protection by genome-wide RNAi. Curr Biol 16: 1344–1350. 10.1016/j.cub.2006.05.047 16824923

[GAD321943CARC57] Verma R, Oania R, Fang R, Smith GT, Deshaies RJ. 2011 Cdc48/p97 mediates UV-dependent turnover of RNA Pol II. Mol Cell 41: 82–92. 10.1016/j.molcel.2010.12.017 21211725 PMC3063307

[GAD321943CARC58] Wang Q, Ma S, Song N, Li X, Liu L, Yang S, Ding X, Shan L, Zhou X, Su D, 2016 Stabilization of histone demethylase PHF8 by USP7 promotes breast carcinogenesis. J Clin Invest 126: 2205–2220. 10.1172/JCI85747 27183383 PMC4887182

[GAD321943CARC59] Wild T, Cramer P. 2012 Biogenesis of multisubunit RNA polymerases. Trends Biochem Sci 37: 99–105. 10.1016/j.tibs.2011.12.001 22260999

[GAD321943CARC60] Wilson MD, Harreman M, Svejstrup JQ. 2013 Ubiquitylation and degradation of elongating RNA polymerase II: the last resort. Biochim Biophys Acta 1829: 151–157. 10.1016/j.bbagrm.2012.08.002 22960598

[GAD321943CARC61] Yang F, Kemp CJ, Henikoff S. 2015 Anthracyclines induce double-strand DNA breaks at active gene promoters. Mutat Res 773: 9–15. 10.1016/j.mrfmmm.2015.01.007 25705119 PMC4332850

[GAD321943CARC62] Yasukawa T, Kamura T, Kitajima S, Conaway RC, Conaway JW, Aso T. 2008 Mammalian Elongin A complex mediates DNA-damage-induced ubiquitylation and degradation of Rpb1. EMBO J 27: 3256–3266. 10.1038/emboj.2008.249 19037258 PMC2609743

[GAD321943CARC63] Zhang Q, Karnak D, Tan M, Lawrence TS, Morgan MA, Sun Y. 2016 FBXW7 facilitates nonhomologous end-joining via K63-linked polyubiquitylation of XRCC4. Mol Cell 61: 419–433. 10.1016/j.molcel.2015.12.010 26774286 PMC4744117

