# Supplementary material for: WWP2 ubiquitylates RNA polymerase II for DNA-PK-dependent transcription arrest and repair at DNA breaks
Source: Genes Dev. 2019 Jun 1;33(11-12):684–704. doi: 10.1101/gad.321943.118 (PMC6546063; doi:10.1101/gad.321943.118)
Supplement: Supplemental Material [file supp_33_11-12_684__index.html]

WWP2 ubiquitylates RNA polymerase II for DNA-PK-dependent transcription arrest and repair at DNA breaks — Supplemental Material 

# WWP2 ubiquitylates RNA polymerase II for DNA-PK-dependent transcription arrest and repair at DNA breaks

## Supplemental Material

- Supplemental\_Table\_S1.xlsx
- Supplemental\_Figures\_and\_Tables.pdf
