# Supplementary material for: WWP2 ubiquitylates RNA polymerase II for DNA-PK-dependent transcription arrest and repair at DNA breaks
Source: Genes Dev. 2019 Jun 1;33(11-12):684–704. doi: 10.1101/gad.321943.118 (PMC6546063; doi:10.1101/gad.321943.118)
Supplement: Supplemental Material [file supp_gad.321943.118_Supplemental_Figures_and_Tables1_.pdf]

Caron_Supplemental Figure S1

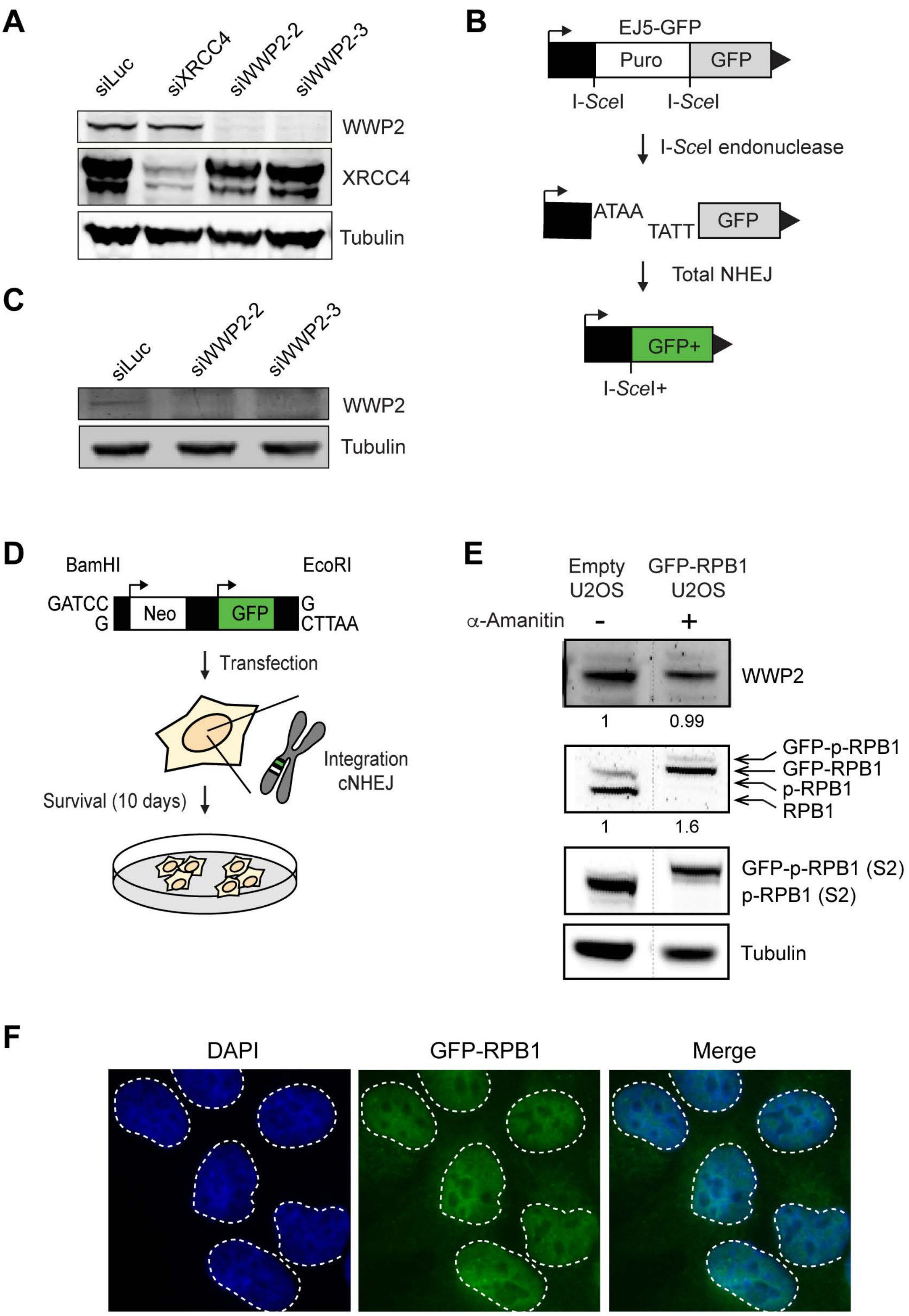

Supplemental Figure S1. DSB repair assays and generation of a stable U2OS GFP-RPB1 cell line

(A) Western blot analysis of XRCC4 and WWP2 expression in cells from Fig. 1A. Tubulin is a loading control.

(B) Schematic representation of the EJ5-GFP reporter for NHEJ.

(C) Western blot analysis of WWP2 expression in cells from Fig. 1B. Tubulin is a loading control.

(D) Schematic representation of the plasmid integration assay.

(E) Western blot analysis of WWP2, RPB1 and p-RPB1 (S2) expression in U2OS and stable U2OS GFP-RPB1 cells. Tubulin is a loading control. Relative expression levels of the indicated proteins in U2OS GFP-RPB1 versus U2OS cells (set to 1) following correction for loading based on tubulin levels is indicated below the blots.

(F) Representative microscopy images showing nuclear localization of GFP-RPB1.

Caron_Supplemental Figure S2

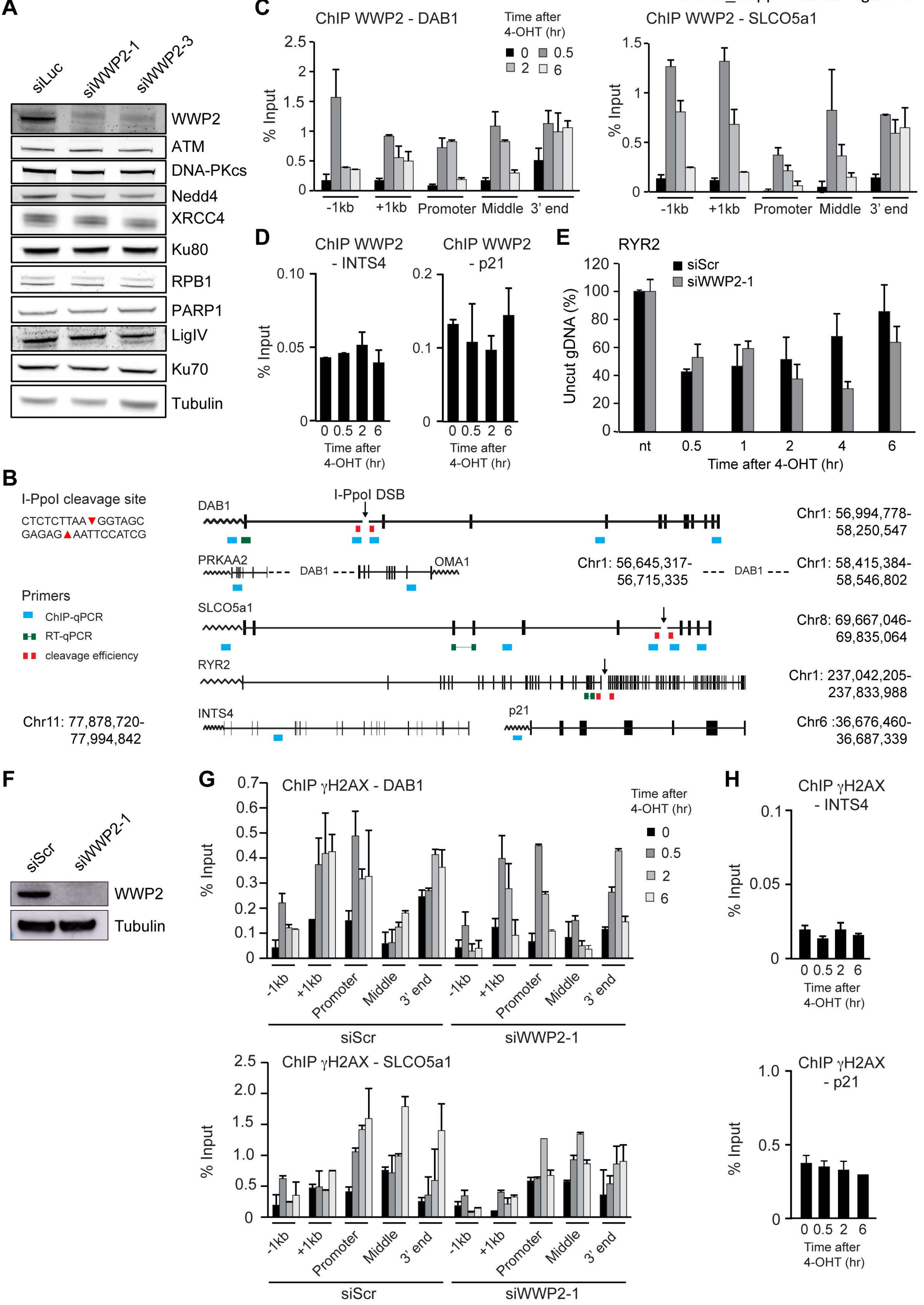

Supplemental Figure S2. WWP2 is recruited to sites of damage and promotes DNA repair

(A) Western blot analysis of the indicated proteins in U2OS cells transfected with the indicated siRNAs. Tubulin is a loading control.

(B) Schematic of the HA-ER-I-PpoI system in U2OS cells used to generate site-specific DSBs at the indicated genes following 4-hydroxytamoxifen (4-OHT) treatment. Blue boxes indicate positions of primers used to monitor protein binding by ChIP-qPCR. Green boxes indicate positions of primers used to quantify mRNA levels of the indicated genes by RT-qPCR. Red boxes indicate positions of primers used to measure the cleavage efficiency of I-PpoI.

(C) ChIP-qPCR against WWP2 in U2OS HA-ER-I-PpoI cells at the indicated time points after (4-OHT) treatment and at the indicated positions at DAB1 and SLCO5a1. The mean ±SD from qPCR replicates of one experiment is shown. This is a repeat of the experiment shown in Fig. 2D.

(D) Same as in D, except that WWP2 levels were monitored at INTS4 and p21.

(E) Cutting efficiency at RYR2 at the indicated time points after (4-OHT) treatment in U2OS HA-ER-I-PpoI cells transfected with the indicated siRNAs. The mean ±SD from qPCR replicates of a representative experiment is shown. A repeat of the experiment is shown in Supplemental Fig. S8A.

(F) Western blot analysis of WWP2 expression in cells from Fig. 2E. Tubulin is a loading control.

(G) ChIP-qPCR against γH2AX in U2OS HA-ER-I-PpoI cells at the indicated time points after (4-OHT) treatment and at the indicated positions at DAB1 and SLCO5a1. The mean ±SD from qPCR replicates of one experiment is shown. A repeat of the experiment shown in Supplemental Fig. S9.

(H) As in G, except that γH2AX levels were monitored at INTS4 and p21.

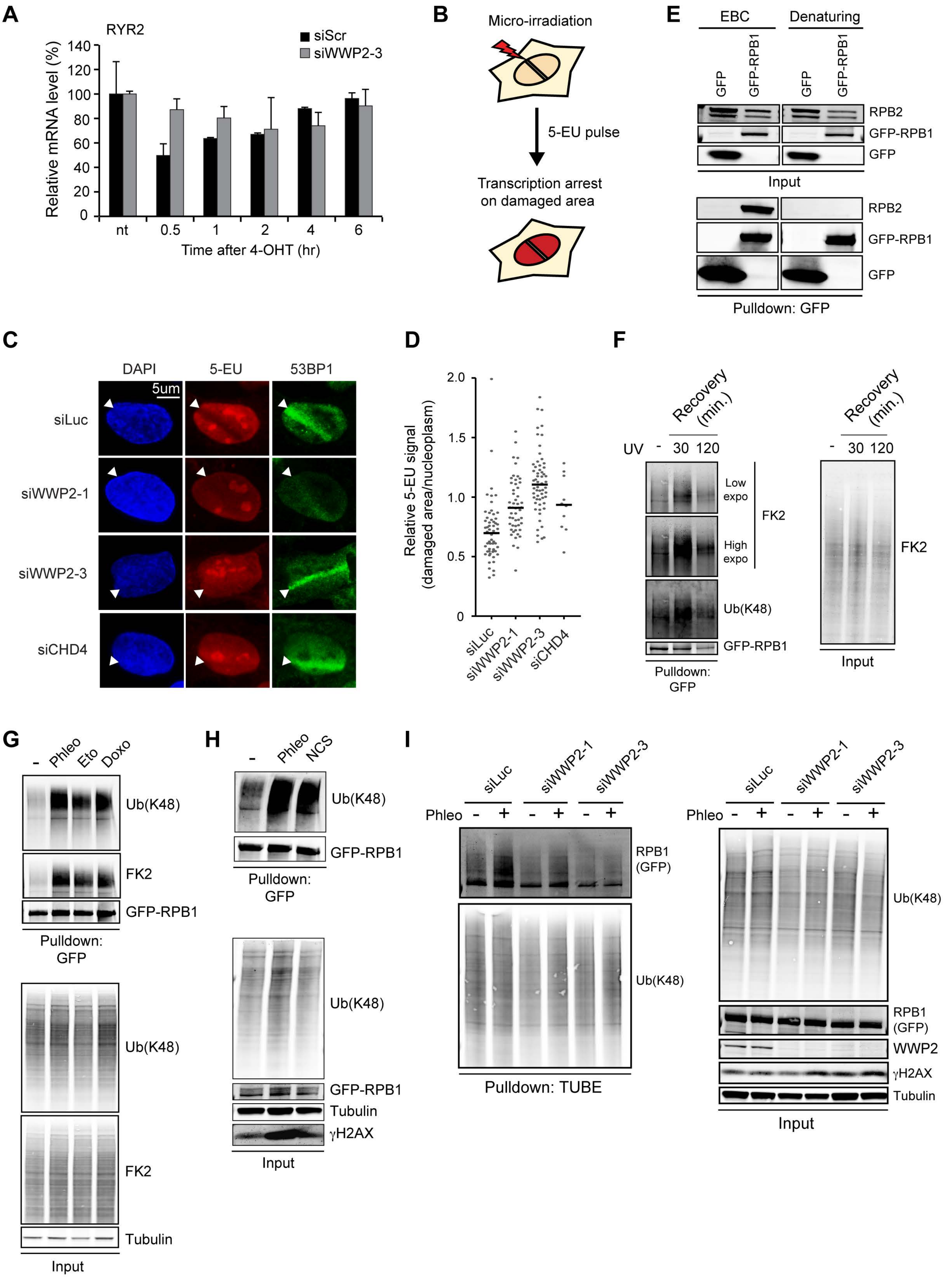
A
RYR2
siScr
siWWP2-3
Relative mRNA level (%)
Time after 4-OHT (hr)
nt 0.5 1 2 4 6
B
Micro-irradiation
5-EU pulse
Transcription arrest on damaged area
E
EBC
Denaturing
GFP
GFP-RPB1
RPB2
GFP-RPB1
GFP
Input
Pulldown: GFP
C
DAPI
5-EU
53BP1
5um
siLuc
siWWP2-1
siWWP2-3
siCHD4
D
Relative 5-EU signal (damaged area/nucleoplasm)
siLuc
siWWP2-1
siWWP2-3
siCHD4
F
Recovery (min.)
UV
- 30 120
Low expo
High expo
FK2
Ub(K48)
GFP-RPB1
Pulldown: GFP
Input
G
- Phleo Eto Doxo
Ub(K48)
FK2
GFP-RPB1
Pulldown: GFP
Tubulin
Input
H
- Phleo NCS
Ub(K48)
GFP-RPB1
Pulldown: GFP
Tubulin
γH2AX
Input
I
siLuc
siWWP2-1
siWWP2-3
Phleo
RPB1 (GFP)
Ub(K48)
Pulldown: TUBE
WWP2
γH2AX
Tubulin
Input

Supplemental Figure S3. WWP2 promotes DSB-induced transcription silencing and RPB1 is ubiquitylated after DNA damage

(A) RT-qPCR analysis of RYR2 expression levels in U2OS ER-I-PpoI cells at the indicated timepoints after (4-OHT) treatment and transfected with the indicated siRNAs. RYR2 mRNA levels are normalized to those of cyclophilin B. The mean ±SD from qPCR replicates of a representative experiment is shown. A repeat of the experiment is shown in Supplemental Fig. S8B.

(B) Schematic of the assay used to monitor nascent transcription by 5-ethynyl uridine (5-EU) incorporation at DNA damage sites induced by UV-A laser micro-irradiation (upper panel).

(C) UV-A laser-micro-irradiation of U2OS cells transfected with the indicated siRNAs and analyzed by immunofluorescence following 5-EU and DAPI incorporation. 53BP1 is a DNA damage marker.

(D) Quantification of nascent transcription in C. Grey dots represent nascent transcription levels in individual nuclei. Black lines represent the mean nascent transcription level in all nuclei analyzed per sample.

(E) Pulldowns of GFP GFP-RPB1 under non-denaturing (EBC) and denaturing conditions in U2OS cells. Blots were probed for RPB2 and GFP.

(F) Pulldowns of GFP-RPB1 under denaturing conditions in untreated and UV-irradiated U2OS cells. Blots were probed for FK2 (ubiquitin), Ub(K48) and GFP.

(G) Pulldowns of GFP-RPB1 under denaturing conditions in untreated and phleomycin (Phleo)-, etoposide (Eto)- or doxorubicin (Doxo)-treated U2OS cells. Cells were also treated with proteasome inhibitor (MG-132) 25 minutes before treatment with DNA damaging agents. Blots were probed for Ub(K48), FK2 (ubiquitin) and GFP. Tubulin is a loading control.

(H) Same as in G, except that phleomycin (Phleo) and neocarsinostatin (NCS) were used to induce DSBs. Blots were probed for Ub(K48), GFP and γH2AX. Tubulin is a loading control.

(I) Pulldown of ubiquitylated proteins by the TUBE approach in U2OS cells stably expressing GFP-RPB1. Cells were treated with the indicated siRNAs and with proteasome inhibitor (MG-132) 25 minutes before treatment with DNA damaging agents. Blots were probed for Ub(K48), GFP, WWP2 and γH2AX. Tubulin is a loading control.

Caron_Supplemental Figure S4

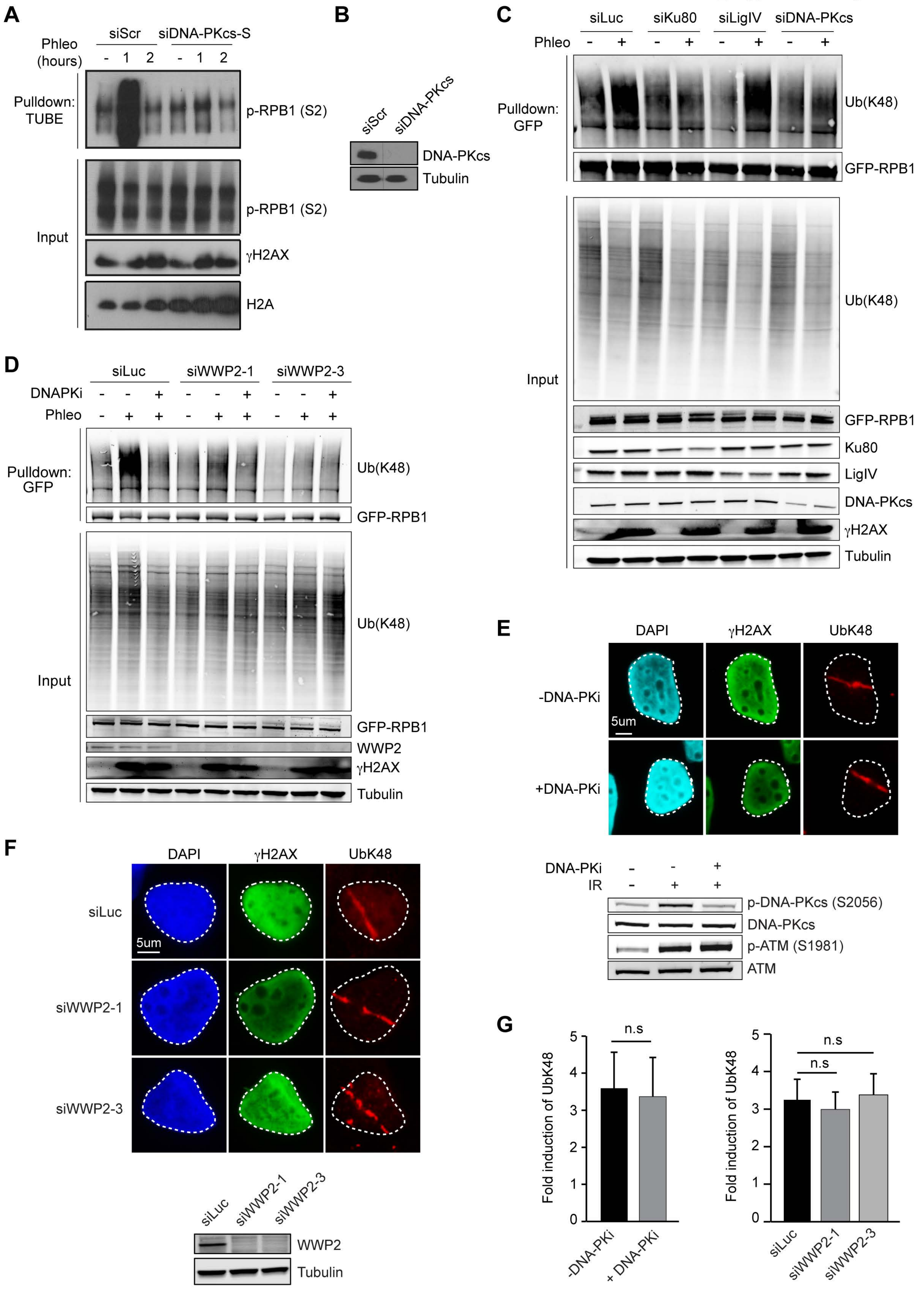

Supplemental Figure S4. DNA-PK and WWP2 co-operate to promote DNA damage-induced RPB1 ubiquitylation

(A) Pulldowns of ubiquitylated proteins in U2OS using the TUBE approach. Cells were treated with a broad-spectrum inhibitor of de-ubiquitylating enzymes (PR169) and the indicated siRNAs before phleomycin (Phleo) treatment. Blots were probed for p-RPB1 (S2), γH2AX and H2A.

(B) Western blot analysis of the indicated proteins in U2OS cells from A. Tubulin is a loading control.

(C) Pulldowns of GFP-RPB1 under denaturing conditions in U2OS cells treated with the indicated siRNAs. Cells were also treated with proteasome inhibitor (MG-132) 25 minutes before the phleomycin treatment. Blots were probed for Ub(K48), GFP, γH2AX, H3, Ku80, LigIV and DNA-PKcs. Tubulin is a loading control.

(D) As in C, except that cells were treated with siRNAs against Luciferase (siLuc) and WWP2 and that blots were probed for WWP2 instead of Ku80, LigIV and DNA-PKcs.

(E) Immunofluorescence images (upper panel) of Ub(K48) accumulation at DNA damage tracks generated by multiphoton laser micro-irradiation in U2OS cells that were left untreated or treated with DNA-PK inhibitor (DNA-PKi). γH2AX is a DNA damage marker. Western blot analysis (lower panel) of DNA-PK activation 1 hour after 10 Gy of irradiation radiation (IR). Blots were probed for p-DNA-PKcs (S2056), DNA-PKcs, p-ATM (S1981) and ATM.

(F) Same as in E, except that cells were transfected with the indicated siRNA (upper panel). Western blot analysis (lower panel) of WWP2 expression levels. Blots were probed for WWP2. Tubulin is a loading control.

(G) Quantification of upper panels in E (left) and F (right). The mean ±S.E.M from 3 independent experiments is shown. Statistical significance was calculated using the Student's t-test (p<0,05 *, p<0,01 **, p<0,001 ***).

Caron_Supplemental Figure S5

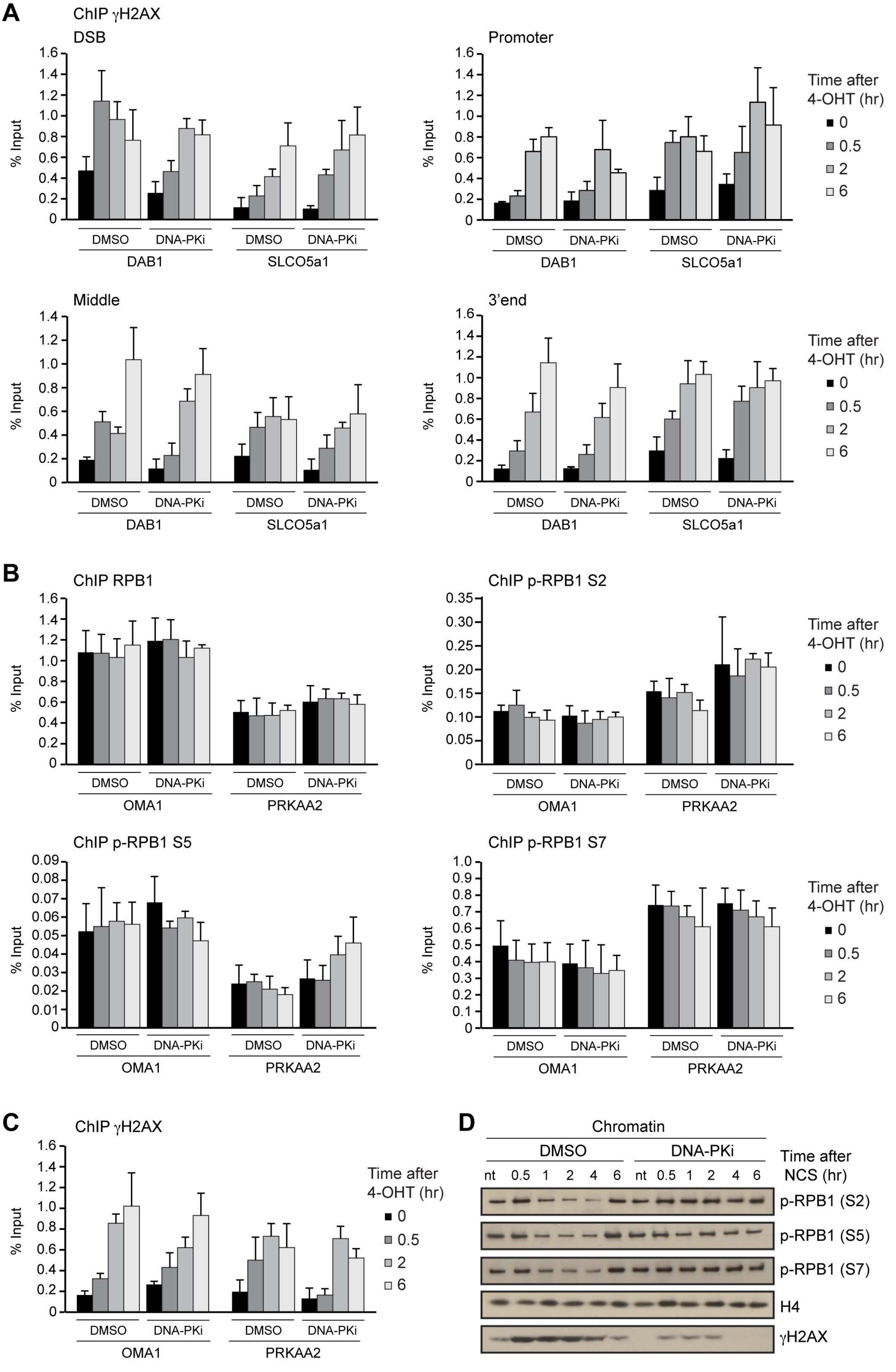

Supplemental Figure S5. DNA-PK regulates RPB1 levels at broken genes

(A) ChIP-qPCR against γH2AX in DMSO-treated (control) and DNA-PKi-treated U2OS HA-ER-I-PpoI cells at the indicated time points after (4-OHT) treatment and at the indicated positions at DAB1 and SLCO5a1. The mean ±SD from qPCR replicates of a representative experiment is shown.

(B) As in A, expect that RPB1 and S2, S5 or S7 phosphorylated (p-)RPB1 were examined at the OMA1 and PRKAA2 genes. A repeat of the experiment is shown in Supplemental Fig. S12A-B.

(C) As in A, expect for OMA1 and PRKAA2. A repeat of the experiment is shown in Supplemental Fig. S12A-B.

(D) Western blot analysis of RPB1 and S2, S5 or S7 phosphorylated (p-)RPB1 levels on chromatin isolated from DMSO-treated (control) and DNA-PKi-treated U2OS cells at the indicated time points after treatment with neocarzinostatin (NCS). H4 is a loading control. γH2AX is a DNA damage marker.

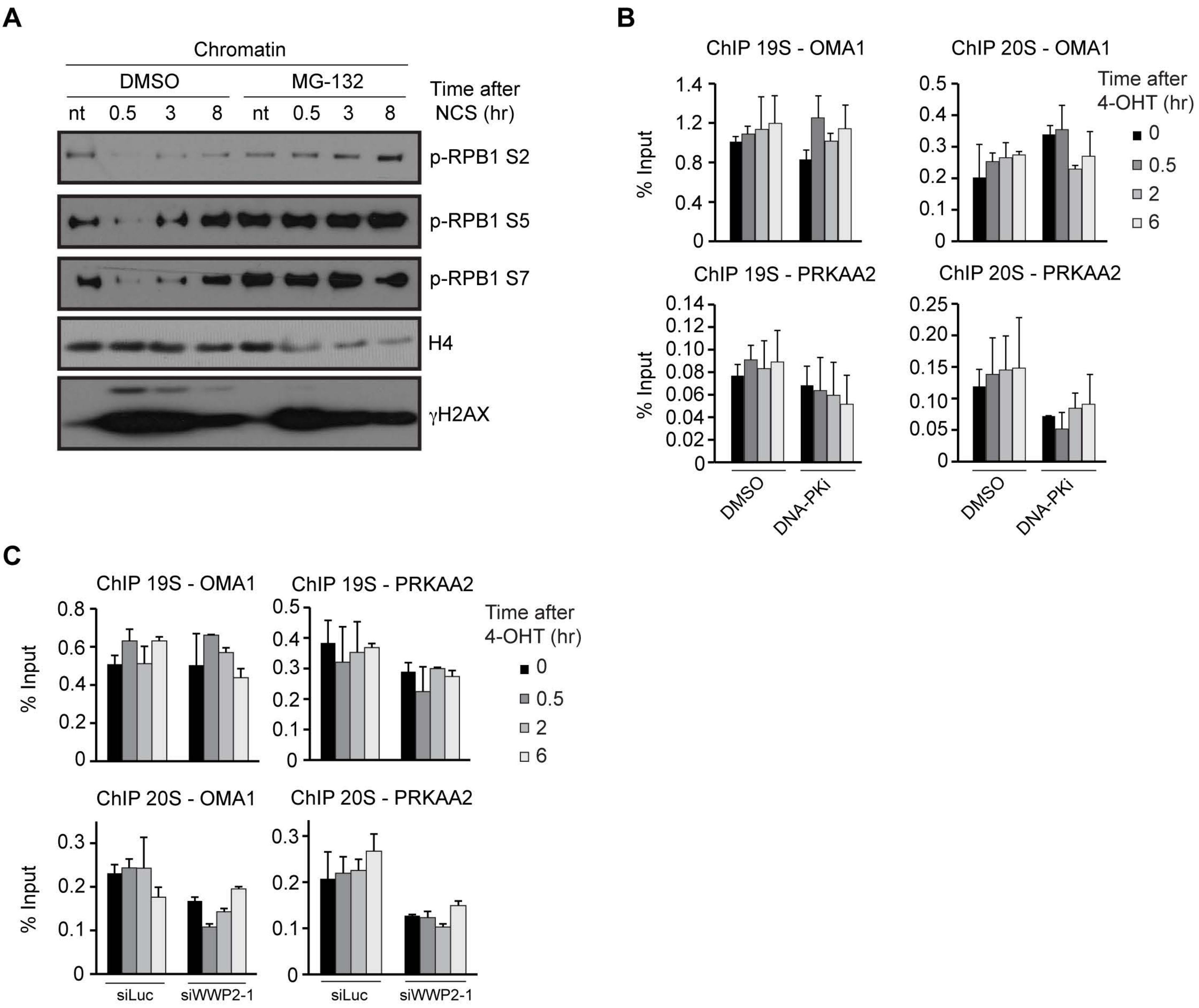
A
Chromatin
DMSO
MG-132
nt 0.5 3 8 nt 0.5 3 8
Time after NCS (hr)
p-RPB1 S2
p-RPB1 S5
p-RPB1 S7
H4
γH2AX
B
ChIP 19S - OMA1
ChIP 20S - OMA1
ChIP 19S - PRKAA2
ChIP 20S - PRKAA2
% Input
Time after 4-OHT (hr)
0
0.5
2
6
DMSO
DNA-PKi
C
ChIP 19S - OMA1
ChIP 19S - PRKAA2
ChIP 20S - OMA1
ChIP 20S - PRKAA2
% Input
Time after 4-OHT (hr)
0
0.5
2
6
siLuc
siWWP2-1

Supplemental Figure S6. Proteasome is recruited to broken genes in a DNA-PKcs- and WWP2-dependent manner

(A) Western blot analysis of S2, S5 or S7 phosphorylated (p-)RPB1 levels on chromatin isolated from DMSO-treated (control) and MG-132-treated U2OS cells at the indicated time points after treatment with neocarzinostatin (NCS). H4 is a loading control. γH2AX is a DNA damage marker.

(B) ChIP-qPCR against the 19S and 20S proteasome in U2OS HA-ER-I-PpoI cells at the indicated time points after (4-OHT) treatment at OMA1 and PRKAA2. The mean ±SD from qPCR replicates of a representative experiment is shown. A repeat of the experiment is shown in Supplemental Fig. S13C.

(C) Same as in B, except that cells were transfected with the indicated siRNA. A repeat of the experiment is shown in Supplemental Fig. S14C.

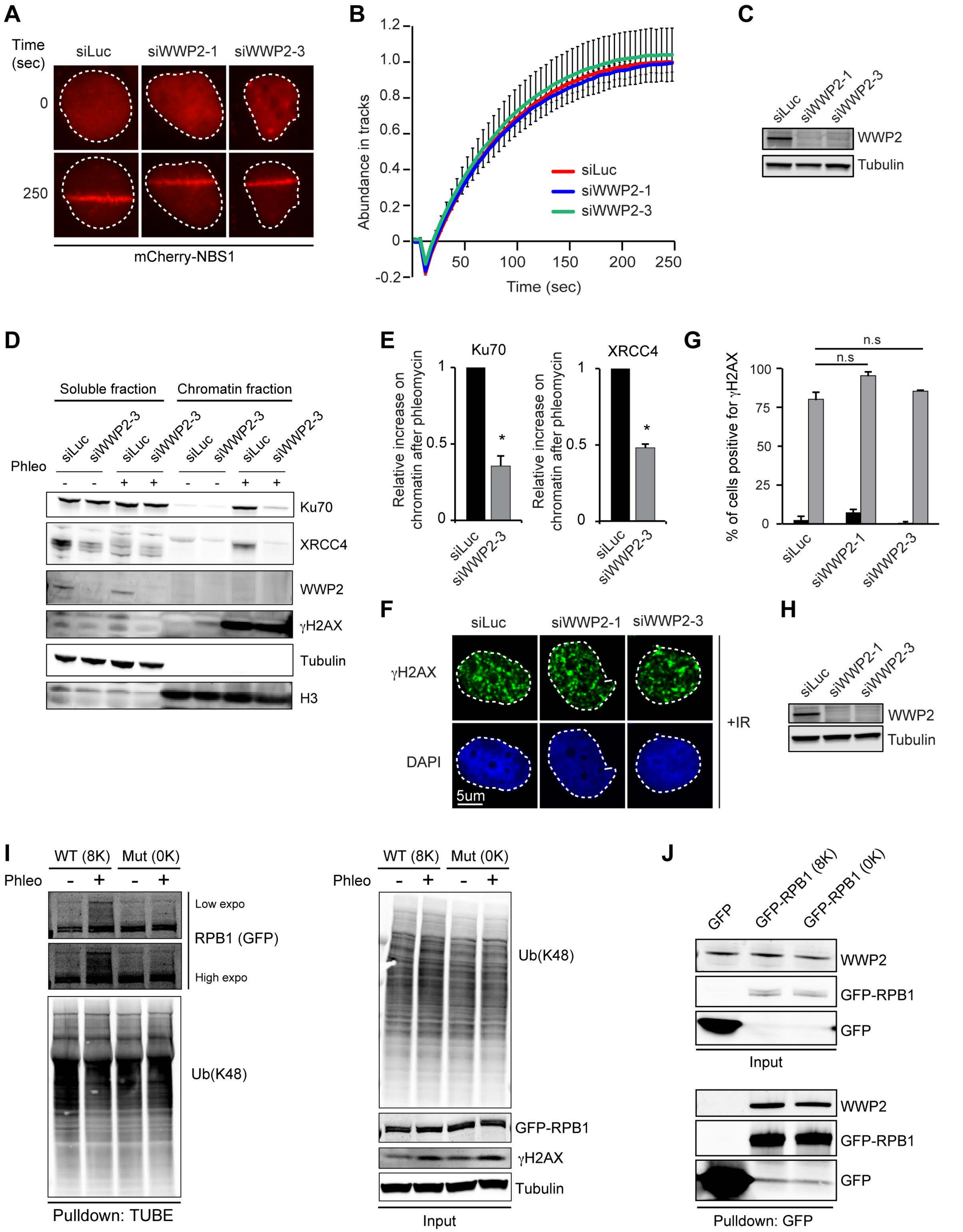
A
Time (sec)
siLuc
siWWP2-1
siWWP2-3
0
250
mCherry-NBS1
B
Abundance in tracks
Time (sec)
siLuc
siWWP2-1
siWWP2-3
C
WWP2
Tubulin
D
Soluble fraction
Chromatin fraction
Phleo
Ku70
XRCC4
WWP2
γH2AX
Tubulin
H3
E
Ku70
XRCC4
Relative increase on chromatin after phleomycin
siLuc
siWWP2-3
F
γH2AX
DAPI
5um
+IR
G
n.s
% of cells positive for γH2AX
H
I
WT (8K)
Mut (0K)
Low expo
RPB1 (GFP)
High expo
Ub(K48)
Pulldown: TUBE
GFP-RPB1
Input
J
GFP
GFP-RPB1 (8K)
GFP-RPB1 (0K)
Pulldown: GFP

Supplemental Figure S7. WWP2 promotes binding of NHEJ factors on damaged chromatin

(A) Immunofluorescence images of mCherry-NBS1 recruitment to DNA damage tracks generated by UV-A laser-micro-irradiation in U2OS cells transfected with the indicated siRNAs and an mCherry-NBS1 expression vector. The mean ±SD from 3 independent experiments is shown.

(B) Quantification of A. The mean ±S.E.M from 3 independent experiments is shown. Statistical significance was calculated using the Student's t-test ($p<0,05$ *, $p<0,01$ **, $p<0,001$ ***).

(C) Western blot analysis of WWP2 expression in cells from A. Blots were probed for WWP2. Tubulin is a loading control.

(D) Western blot analysis of the indicated proteins in soluble and chromatin fractions from untreated and Phleomycin (Phleo-)treated U2OS cells. Tubulin and H4 are loading controls. γH2AX is a DNA damage marker. A representative experiment is shown.

(E) Quantifications of Ku70 and XRCC4 levels in D. The mean ±SD from 3 independent experiments is shown. Statistical significance was calculated using the Student's t-test ($p<0,05$ *, $p<0,01$ **, $p<0,001$ ***).

(F) Immunofluorescence images of γH2AX foci formation 1 hour after 10 Gy of IR in U2OS cells transfected with the indicated siRNAs.

(G) Quantification of F. The mean ±S.E.M from 3 independent experiments is shown. Statistical significance was calculated using the Student's t-test ($p<0,05$ *, $p<0,01$ **, $p<0,001$ ***).

(H) Western blot analysis of WWP2 expression in cells from F. Blots were probed for WWP2. Tubulin is a loading control.

(I) Pulldown of ubiquitylated proteins using the TUBE approach in NIH3T3 cells stably expressing wild-type (8K) or mutant (0K) GFP-RPB1. Cells were treated with proteasome inhibitor (MG-132) 25 minutes before phleomycin treatment. Blots were probed for GFP, Ub(K48) and γH2AX. Tubulin is a loading control.

(J) Pulldown of GFP from U2OS cells transiently expressing GFP, wild-type (8K) GFP-RPB1 or mutant (0K) GFP-RPB1. Blots were probed for WWP2 and GFP.

Caron_Supplemental Figure S8

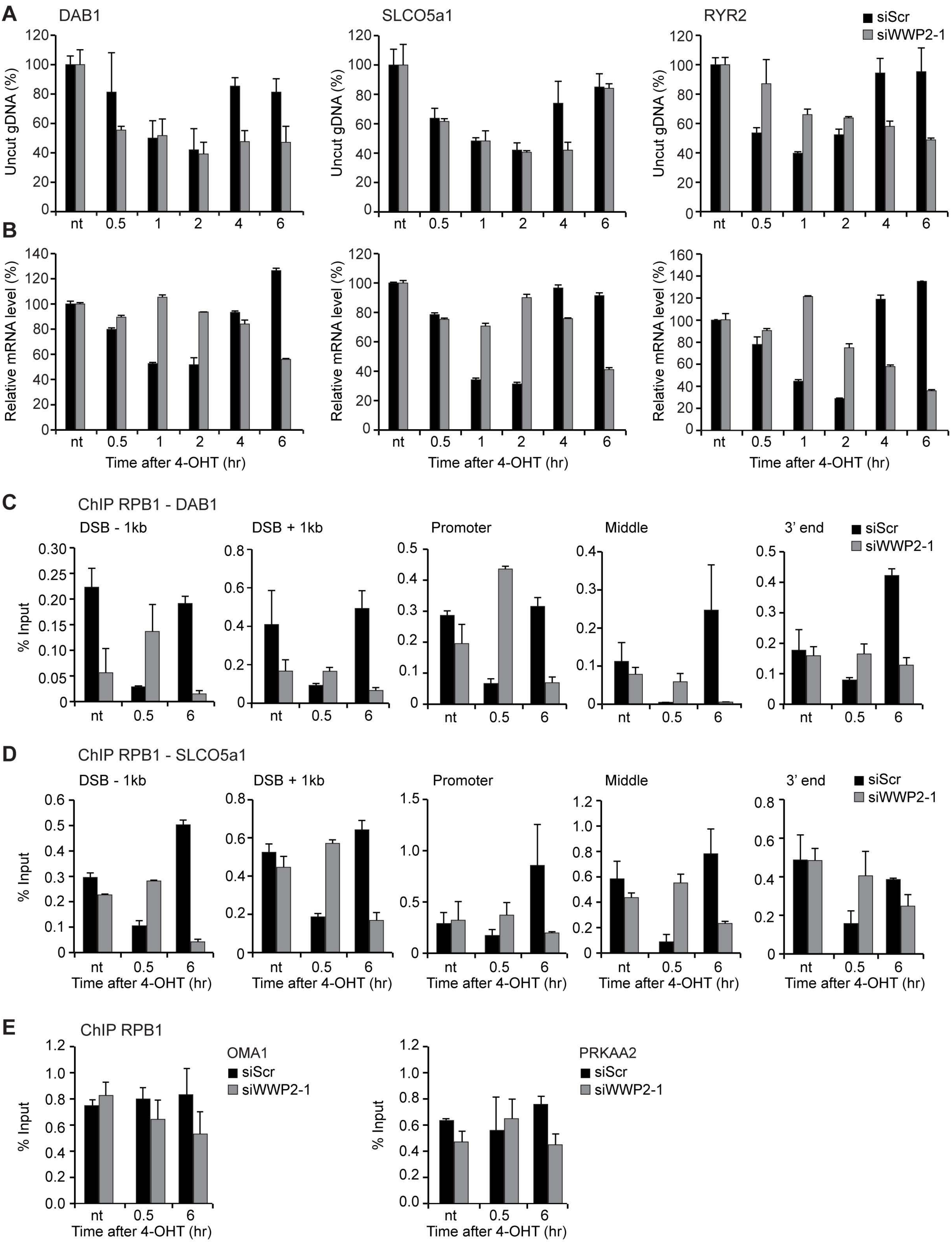

Supplemental Figure S8. WWP2 promotes DNA repair and transcription arrest at broken genes

(A) Repeat of the experiments shown in Fig. 2H and Supplemental Fig. S2E.

(B) Repeat of the experiments shown in Fig. 3A-B and Supplemental Fig. S3A.

(C) Repeat of the experiments shown in Fig. 3C.

(D) Repeat of the experiments shown in Fig. 3D.

(E) Repeat of the experiments shown in Fig. 3E.

Caron_Supplemental Figure S9

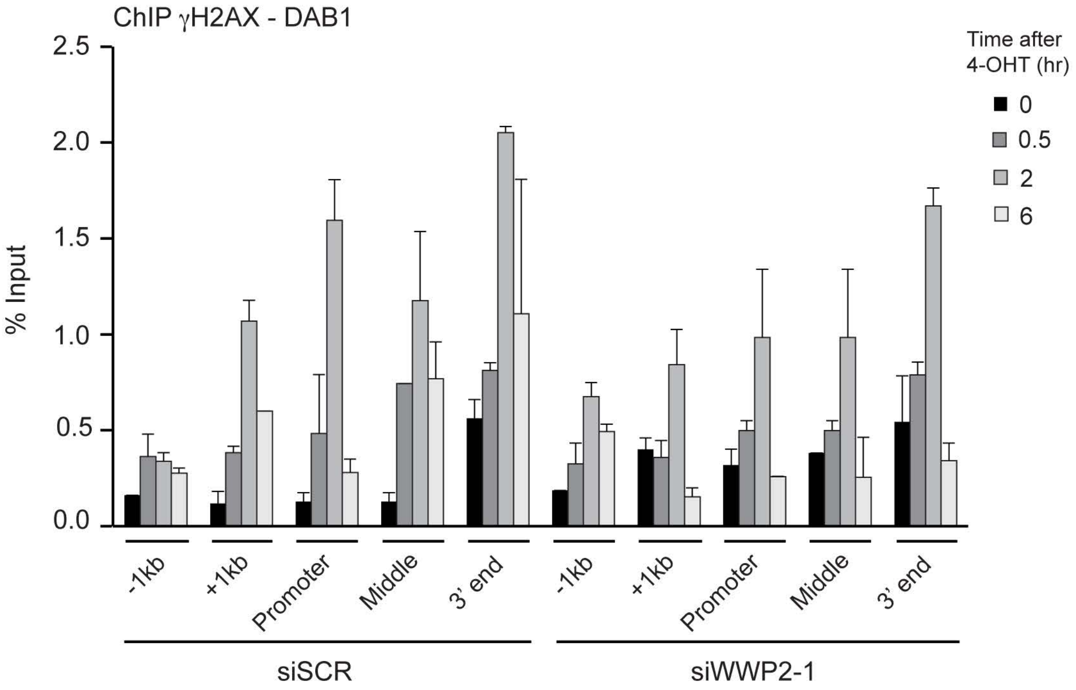

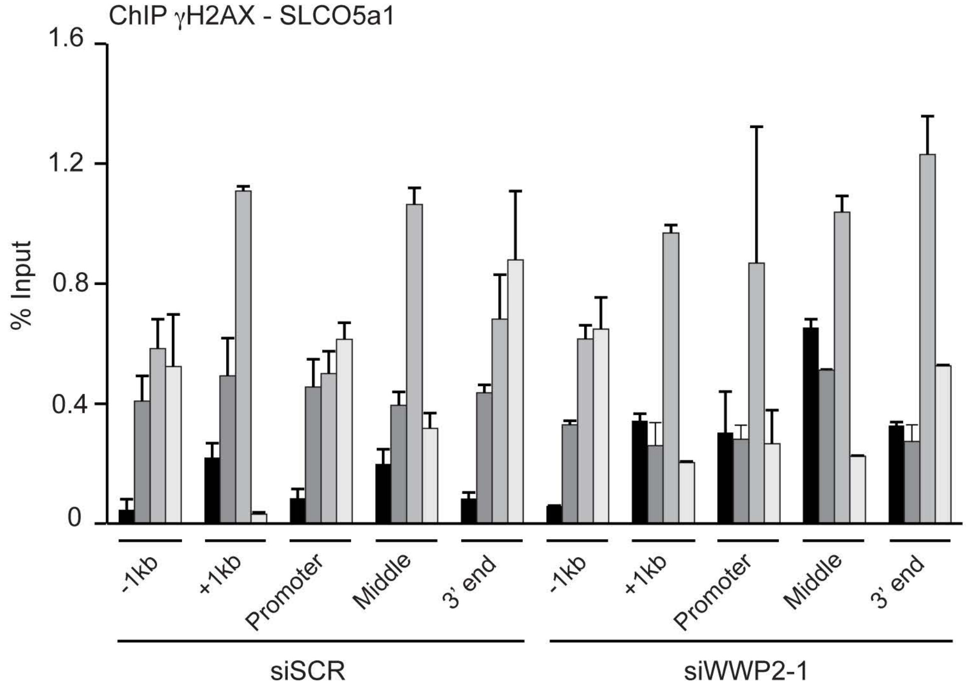

Supplemental Figure S9. Induction of γH2AX in WWP2-depleted cells

(A) Repeat of the experiments shown in Supplemental Fig. 2G.

(B) Repeat of the experiments shown in Supplemental Fig. 2G.

Caron_Supplemental Figure S10

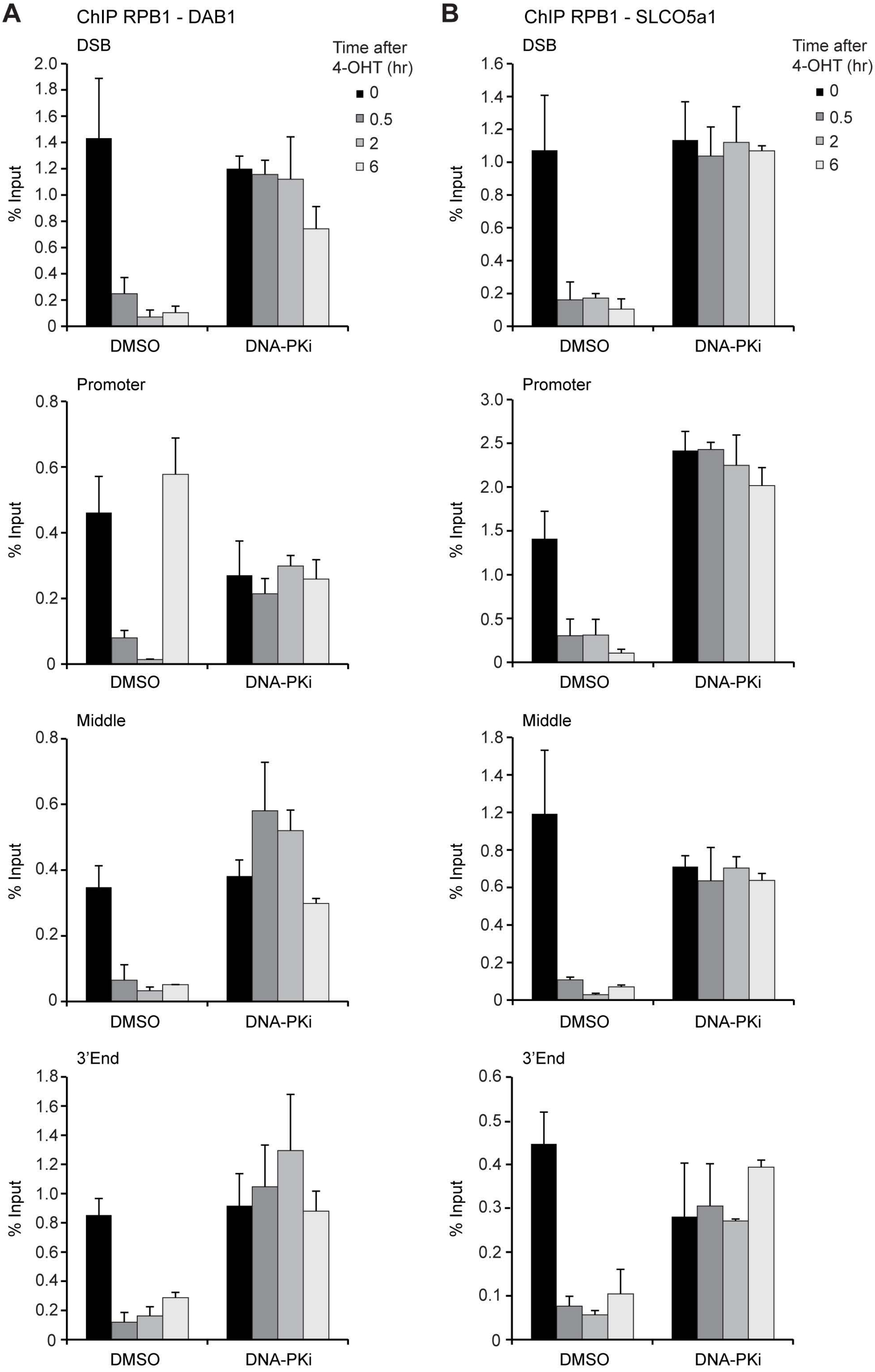

Supplemental Figure S10. DNA-PK controls RPB1 levels at broken genes

(A) Repeat of the experiments shown in Fig. 4C.

(B) Repeat of the experiments shown in Fig. 4D.

Caron_Supplemental Figure S11

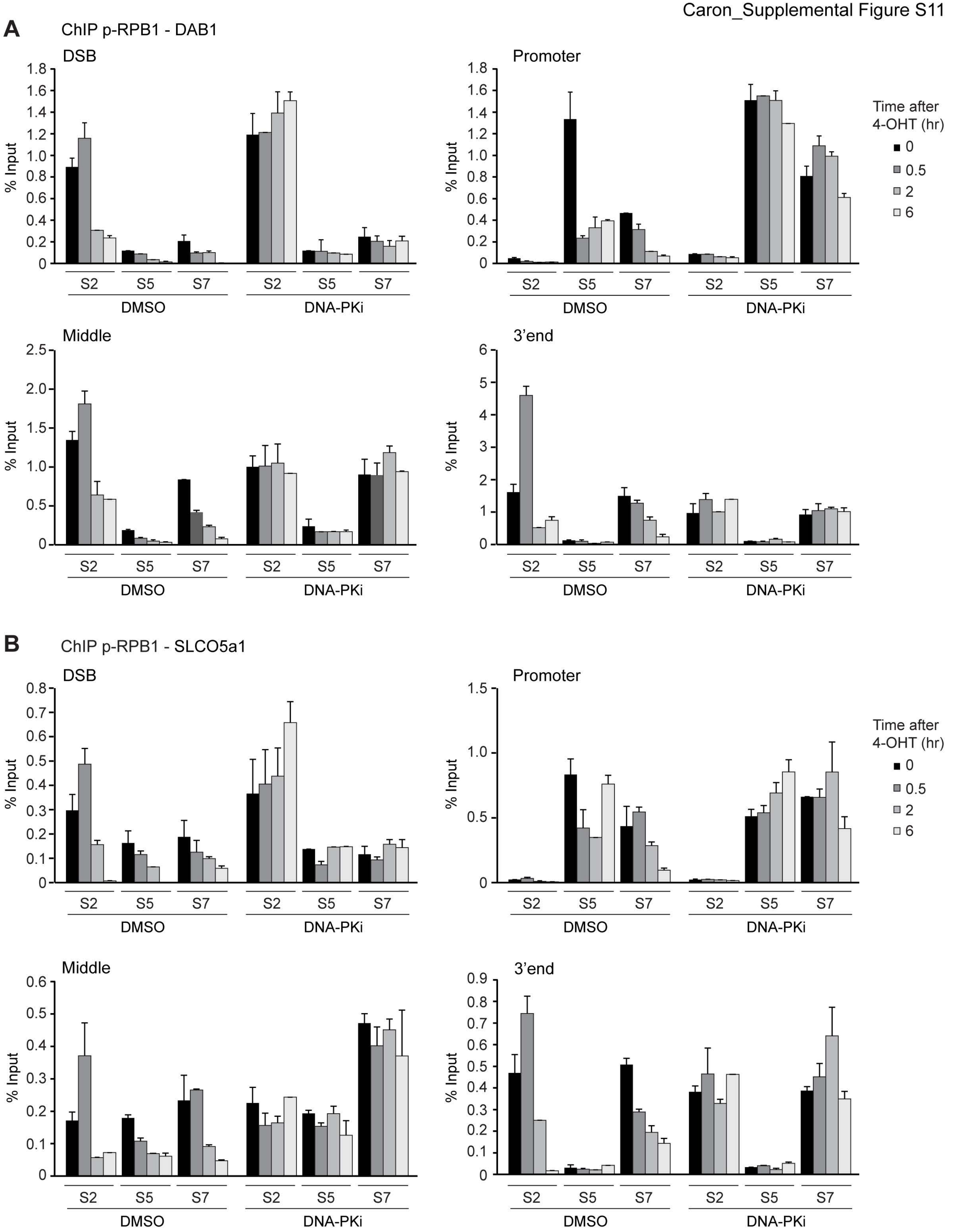

Supplemental Figure S11. DNA-PK controls phosphorylated RPB1 levels at broken genes

(A) Repeat of the experiments shown in Fig. 4C.

(B) Repeat of the experiments shown in Fig. 4D.

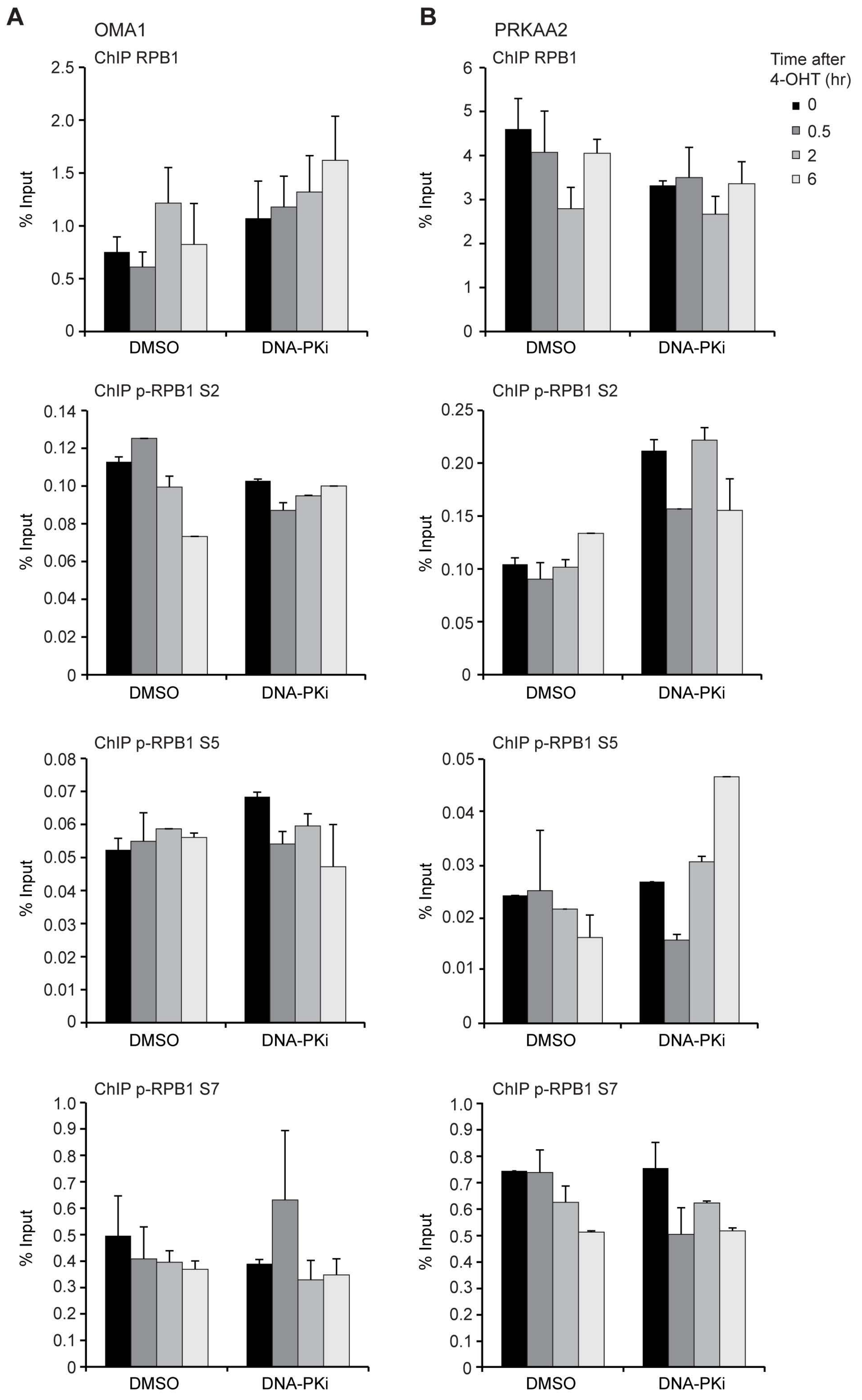
A
OMA1
ChIP RPB1
% Input
DMSO
DNA-PKi
ChIP p-RPB1 S2
ChIP p-RPB1 S5
ChIP p-RPB1 S7
B
PRKAA2
Time after
4-OHT (hr)
0
0.5
2
6

Supplemental Figure S12. RPB1 and phosphorylated RPB1 levels remain unaffected at non-damaged genes

Repeat of the experiments shown in Supplemental Fig. S5B.

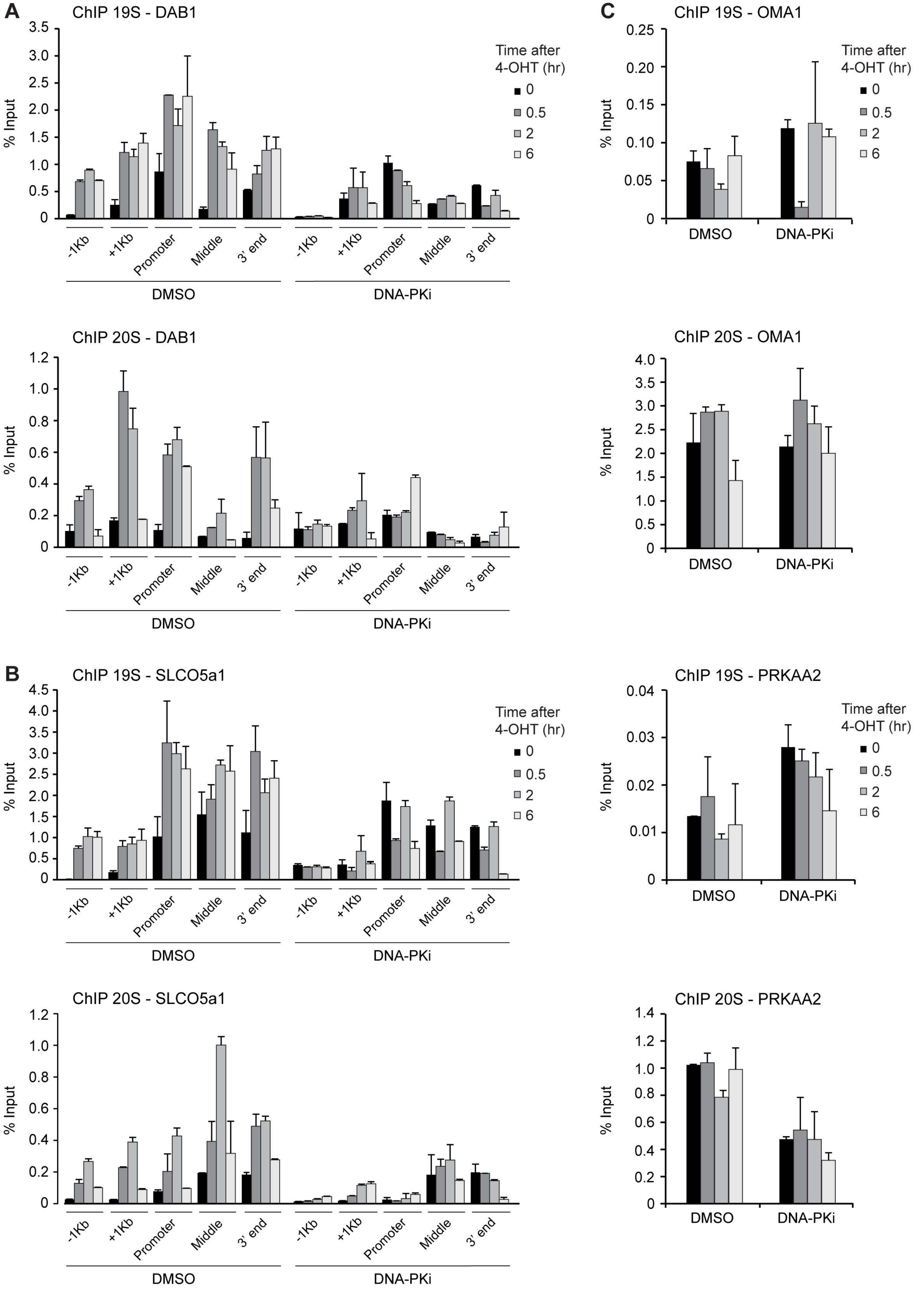
A
ChIP 19S - DAB1
% Input
Time after 4-OHT (hr)
0
0.5
2
6
-1Kb
+1Kb
Promoter
Middle
3' end
DMSO
DNA-PKi
ChIP 20S - DAB1
B
ChIP 19S - SLCO5a1
ChIP 20S - SLCO5a1
C
ChIP 19S - OMA1
ChIP 20S - OMA1
ChIP 19S - PRKAA2
ChIP 20S - PRKAA2

Supplemental Figure S13. Proteasome is recruited to broken genes

(A) Repeat of the experiments shown in Fig. 5A-B.

(B) Repeat of the experiments shown in Fig. 5C-D.

(C) Repeat of the experiments shown in Supplemental Fig. 6B.

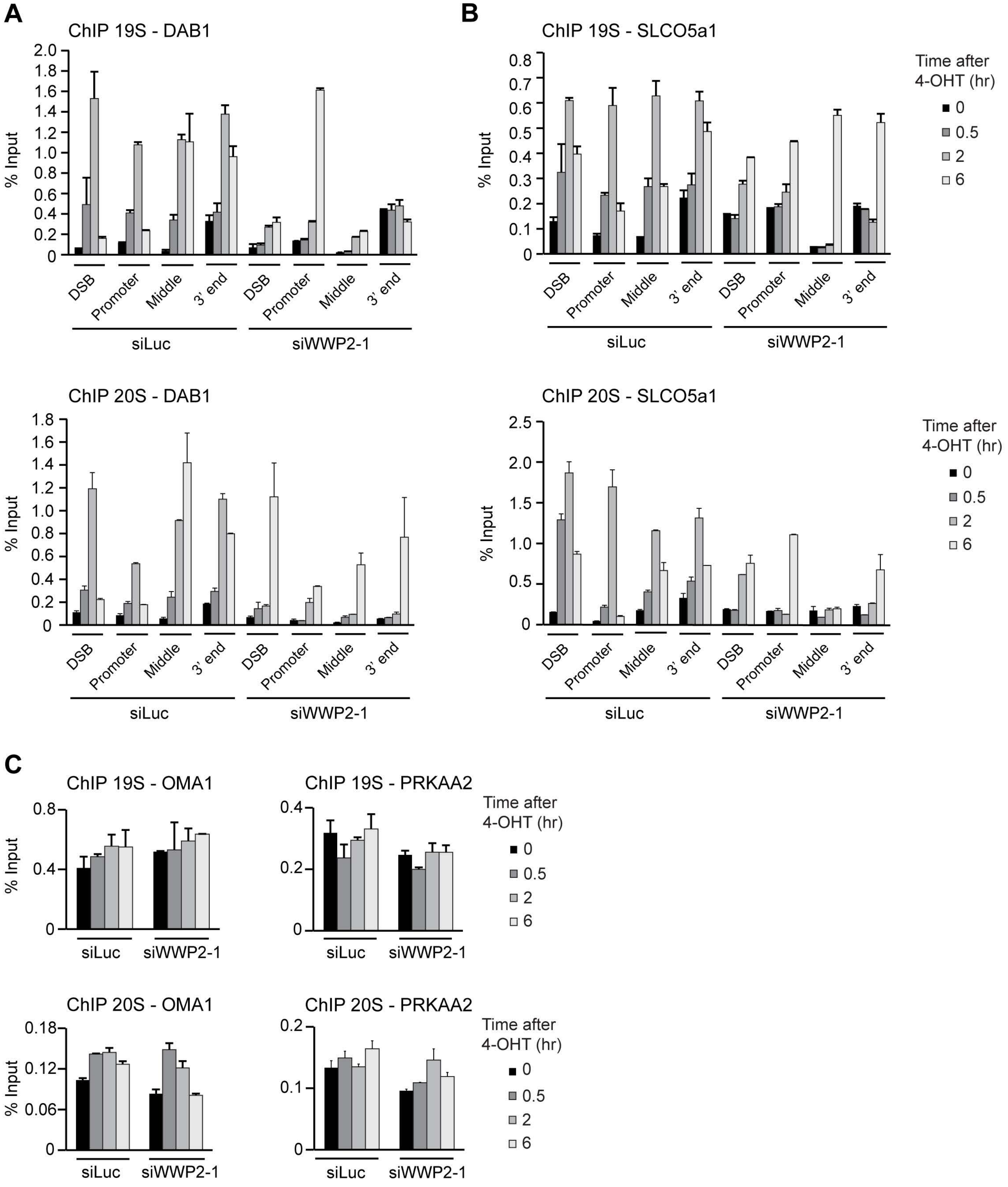
A
ChIP 19S - DAB1
% Input
DSB
Promoter
Middle
3' end
siLuc
siWWP2-1
ChIP 20S - DAB1
B
ChIP 19S - SLCO5a1
ChIP 20S - SLCO5a1
Time after 4-OHT (hr)
0
0.5
2
6
C
ChIP 19S - OMA1
ChIP 19S - PRKAA2
ChIP 20S - OMA1
ChIP 20S - PRKAA2

Supplemental Figure S14. Proteasome is recruited to broken genes

(A) Repeat of the experiments shown in Fig. 5E-F.

(B) Repeat of the experiments shown in Fig. 5G-H.

(C) Repeat of the experiments shown in Supplemental Fig. S6C.

Supplemental Table S1: Proteins identified as interactors of WWP2 by SILAC LC-MS/MS

Light (L): U2OS-GFP
Heavy (H): U2OS-WWP2-GFP

Protein names	Gene names	Fasta headers	Ratio H/L
S1 RNA-binding domain-containing protein 1	SRBD1	sp|Q8N5C6-2|SRB	47,74
RNA-binding protein 14	RBM14	sp|Q96PK6|RBM1	43,47
WD repeat-containing protein 92	WDR92;DKFZp434E	sp|Q96MX6|WDR9	42,48
DNA-directed RNA polymerase;DNA-directed	POLR2B	tr|C9J2Y9|C9J2Y9_	42,12
DNA-directed RNA polymerase II subunit RP	POLR2I	sp|P36954|RPB9_	41,55
DNA-directed RNA polymerase II subunit RP	POLR2C	sp|P19387|RPB3_	41,45
Heterogeneous nuclear ribonucleoprotein U-	HNRNPUL1	sp|Q9BUJ2-2|HNR	39,08
Low-density lipoprotein receptor class A dom	LDLRAD4	tr|K7EN38|K7EN3	38,70
DNA-directed RNA polymerase II subunit RP	POLR2D	sp|O15514|RPB4_	38,34
Cleavage and polyadenylation specificity fac	CPSF7	sp|Q8N684-2|CPS	37,88
NEDD4-like E3 ubiquitin-protein ligase WWP	WWP1	sp|Q9H0M0-6|WW	37,07
60S ribosomal protein L38	RPL38	sp|P63173|RL38_	37,05
DNA-directed RNA polymerases I, II, and III	POLR2F	tr|U3KQS8|U3KQS	36,37
Probable ubiquitin carboxyl-terminal hydrolas	USP9X	sp|Q93008-1|USP	36,09
BRISC and BRCA1-A complex member 1	BABAM1	tr|M0R0I0|M0R0I0_	35,41
Cleavage and polyadenylation specificity fac	CPSF6	tr|F8WJN3|F8WJN	34,77
DNA-directed RNA polymerases I, II, and III	POLR2H	tr|C9JLU1|C9JLU1_	33,88
DNA-directed RNA polymerase II subunit GR	POLR2M;GCOM1;G	sp|P0CAP2|GRL1	33,73
60S ribosomal protein L35	RPL35	sp|P42766|RL35_	32,67
BRISC complex subunit Abro1	FAM175B	sp|Q15018|F175B	31,31
Protein UXT	UXT	sp|Q9UBK9|UXT_	31,17
Protein PRRC2A	PRRC2A	sp|P48634|PRC2A	30,78
DNA-directed RNA polymerase II subunit RP	POLR2J;POLR2J1;	sp|P52435|RPB11	30,78
DNA-directed RNA polymerases I, II, and III	POLR2E	sp|P19388|RPAB1	30,56
Y-box-binding protein 3	YBX3	sp|P16989-2|YBO	30,34
NEDD4-like E3 ubiquitin-protein ligase WWP	WWP2	sp|O00308|WWP2_	30,22
ATP-dependent DNA helicase Q5	RECQL5	tr|Q6P4G0|Q6P4G	30,06
Transcription elongation factor SPT5	SUPT5H	sp|O00267-2|SPT	29,91
60S ribosomal protein L8	RPL8	sp|P62917|RL8_H	29,79
Replication factor C subunit 5	RFC5	sp|P40937-2|RFC5	29,05
General transcription factor IIF subunit 2	GTF2F2	sp|P13984|T2FB_	28,69
Eukaryotic translation initiation factor 3 subu	EIF3B	sp|P55884|EIF3B_	28,42
ATP-dependent RNA helicase A	DHX9	sp|Q08211|DHX9_	28,37
Transcription activator BRG1	SMARCA4	tr|Q9HBD4|Q9HBD	28,36
Cleavage and polyadenylation specificity fac	NUDT21	sp|O43809|CPSF5	28,08
DNA-directed RNA polymerase II subunit RP	POLR2A	sp|P24928|RPB1_	27,80
DNA-directed RNA polymerases I, II, and III	POLR2L	sp|P62875|RPAB5	27,43
60S acidic ribosomal protein P2	RPLP2	sp|P05387|RLA2_	27,41
Proliferation-associated protein 2G4	PA2G4	sp|Q9UQ80|PA2G	27,20
Putative RNA polymerase II subunit B1 CTD	RPAP2	sp|Q8IXW5|RPAP	27,08
Protein FAM168A	FAM168A	sp|Q92567-2|F168	26,88
DNA-directed RNA polymerase II subunit RP	POLR2G	sp|P62487|RPB7_	26,70
60S ribosomal protein L37;Ribosomal protei	RPL37	sp|P61927|RL37_	26,62
40S ribosomal protein S8	RPS8	tr|Q5JR95|Q5JR95	26,55
60S ribosomal protein L30	RPL30	sp|P62888|RL30_	26,32
40S ribosomal protein S15a	RPS15A	sp|P62244|RS15A	26,13
60S ribosomal protein L27	RPL27	sp|P61353|RL27_	25,89
Transcription elongation factor SPT4	SUPT4H1	tr|D3DTZ5|D3DTZ5	24,94
RNA polymerase II-associated protein 3	RPAP3	sp|Q9H6T3|RPAP	24,91
WD repeat-containing protein 5	WDR5	sp|P61964|WDR5_	24,90

40S ribosomal protein S9	RPS9	sp|P46781|RS9_H	24,72
Transducin beta-like protein 2	TBL2	tr|E9PF19|E9PF19	24,57
Histone H1.1	HIST1H1A	sp|Q02539|H11_H	24,48
60S ribosomal protein L10	RPL10	tr|F8W7C6|F8W7C	24,45
p53 and DNA damage-regulated protein 1	PDRG1	sp|Q9NUG6|PDRG	24,41
60S ribosomal protein L32	RPL32	tr|F8W727|F8W727	24,32
60S ribosomal protein L22	RPL22	tr|K7EP65|K7EP65	24,28
40S ribosomal protein S16	RPS16	sp|P62249|RS16_	24,27
ATP-dependent RNA helicase DDX1	DDX1	sp|Q92499|DDX1_	24,18
40S ribosomal protein S6	RPS6	sp|P62753|RS6_H	24,12
60S ribosomal protein L22-like 1	RPL22L1	tr|C9JYQ9|C9JYQ9	24,12
40S ribosomal protein S3a	RPS3A	sp|P61247|RS3A_	23,90
40S ribosomal protein SA	RPSA;RPSAP58	tr|C9J9K3|C9J9K3_	23,85
40S ribosomal protein S29	RPS29	sp|P62273|RS29_	23,77
60S ribosomal protein L7	RPL7	sp|P18124|RL7_H	23,75
40S ribosomal protein S23	RPS23	sp|P62266|RS23_	23,38
60S ribosomal protein L13	RPL13	sp|P26373|RL13_	23,31
Lys-63-specific deubiquitinase BRCC36	BRCC3	sp|P46736-2|BRC	23,29
60S ribosomal protein L6	RPL6	sp|Q02878|RL6_H	23,23
60S ribosomal protein L3	RPL3	sp|P39023|RL3_H	23,17
40S ribosomal protein S14	RPS14	sp|P62263|RS14_	23,14
40S ribosomal protein S24	RPS24	tr|E7ETK0|E7ETK	23,10
Ribosomal protein L19;60S ribosomal protei	RPL19	tr|J3QR09|J3QR09	23,10
Ras GTPase-activating protein-binding prote	G3BP1	sp|Q13283|G3BP1	23,07
Polyadenylate-binding protein 1;Polyadenyl	PABPC1;PABPC3	sp|P11940|PABP1	23,06
Putative 60S ribosomal protein L39-like 5;60	RPL39P5;RPL39	sp|Q59GN2|R39L5	23,06
Ras GTPase-activating protein-binding prote	G3BP2	sp|Q9UN86-2|G3B	22,97
40S ribosomal protein S15	RPS15	tr|K7EM56|K7EM5	22,89
Interleukin enhancer-binding factor 2	ILF2	sp|Q12905|ILF2_H	22,87
60S ribosomal protein L27a	RPL27A	tr|E9PLL6|E9PLL6	22,81
40S ribosomal protein S12	RPS12	sp|P25398|RS12_	22,69
60S ribosomal protein L35a	RPL35A	sp|P18077|RL35A	22,59
40S ribosomal protein S13	RPS13	sp|P62277|RS13_	22,54
40S ribosomal protein S11	RPS11	tr|M0QZC5|M0QZC	22,50
40S ribosomal protein S28	RPS28	sp|P62857|RS28_	22,33
60S ribosomal protein L26	RPL26	sp|P61254|RL26_	22,20
60S ribosomal protein L14	RPL14	sp|P50914|RL14_	22,14
40S ribosomal protein S25	RPS25	sp|P62851|RS25_	22,13
60S ribosomal protein L31	RPL31	sp|P62899|RL31_	21,82
60S ribosomal protein L4	RPL4	sp|P36578|RL4_H	21,67
Nucleophosmin	NPM1	sp|P06748-3|NPM_	21,46
Angiomotin	AMOT	sp|Q4VCS5|AMOT	21,42
Serine/arginine-rich splicing factor 7	SRSF7	tr|C9JAB2|C9JAB2	21,38
Splicing factor, proline- and glutamine-rich	SFPQ	sp|P23246|SFPQ_	21,33
USP6 N-terminal-like protein	USP6NL	sp|Q92738|US6NL	21,29
60S ribosomal protein L12	RPL12	sp|P30050|RL12_	21,18
60S acidic ribosomal protein P1	RPLP1	sp|P05386|RLA1_	21,17
40S ribosomal protein S18	RPS18	sp|P62269|RS18_	21,15
Kinesin-like protein KIF2A	KIF2A	sp|O00139-2|KIF2	21,09
60S ribosomal protein L23	RPL23	sp|P62829|RL23_	21,04
40S ribosomal protein S7	RPS7	sp|P62081|RS7_H	21,03
60S ribosomal protein L13a;Putative 60S rib	RPL13A;RPL13AP3	sp|P40429|RL13A	20,99
60S ribosomal protein L5	RPL5	sp|P46777|RL5_H	20,96
40S ribosomal protein S19	RPS19	sp|P39019|RS19_	20,94
60S ribosomal protein L18a	RPL18A	tr|M0R3D6|M0R3D	20,94
60S ribosomal protein L15;Ribosomal protei	RPL15	sp|P61313|RL15_	20,82

60S acidic ribosomal protein P0;60S acidic ri	RPLP0;RPLP0P6	sp|P05388|RLA0_	20,82
RuvB-like 1	RUVBL1	sp|Q9Y265|RUVB1	20,79
40S ribosomal protein S20	RPS20	sp|P60866|RS20_	20,76
60S ribosomal protein L7a	RPL7A	sp|P62424|RL7A_	20,71
60S ribosomal protein L37a;Putative 60S rib	RPL37A;RPL37AP8	tr|C9J4Z3|C9J4Z3_	20,64
Fragile X mental retardation syndrome-relate	FXR1	sp|P51114|FXR1_	20,64
Heterogeneous nuclear ribonucleoprotein M	HNRNPM	sp|P52272-2|HNRI	20,58
RuvB-like 2	RUVBL2	sp|Q9Y230|RUVB2	20,56
40S ribosomal protein S27	RPS27	tr|Q5T4L4|Q5T4L4	20,50
Interleukin enhancer-binding factor 3	ILF3	sp|Q12906|ILF3_H	20,47
Eukaryotic translation initiation factor 3 subu	EIF3A	sp|Q14152|EIF3A_	20,41
60S ribosomal protein L36	RPL36	sp|Q9Y3U8|RL36_	20,40
40S ribosomal protein S10;Putative 40S ribo	RPS10;RPS10-NUD	sp|P46783|RS10_	20,30
60S ribosomal protein L18	RPL18	tr|G3V203|G3V203	20,28
Eukaryotic translation initiation factor 3 subu	EIF3C;EIF3CL	tr|H3BRV0|H3BRV(	20,23
Prolyl 3-hydroxylase 3	LEPREL2	tr|G8JLI6|G8JLI6_	20,22
Serine/arginine-rich splicing factor 1	SRSF1	tr|J3KTL2|J3KTL2_	20,20
40S ribosomal protein S2	RPS2	sp|P15880|RS2_H	20,20
60S ribosomal protein L34	RPL34	sp|P49207|RL34_	20,03
60S ribosomal protein L21	RPL21	sp|P46778|RL21_	20,01
Probable ATP-dependent RNA helicase DD)	DDX47	sp|Q9H0S4|DDX47	19,93
40S ribosomal protein S5;40S ribosomal pro	RPS5	tr|M0R0F0|M0R0F(	19,92
Tyrosine-protein phosphatase non-receptor	PTPN14	sp|Q15678|PTN14	19,84
60S ribosomal protein L11	RPL11	sp|P62913-2|RL11	19,76
60S ribosomal protein L23a	RPL23A	sp|P62750|RL23A	19,75
Serine/arginine-rich splicing factor 6	SRSF6	sp|Q13247-3|SRSI	19,74
60S ribosomal protein L9	RPL9	tr|D6RAN4|D6RAN	19,66
Eukaryotic translation initiation factor 3 subu	EIF3H	tr|B3KS98|B3KS98	19,65
Protein PRRC2C	PRRC2C	tr|E7EPN9|E7EPN	19,64
40S ribosomal protein S3	RPS3	sp|P23396|RS3_H	19,63
60S ribosomal protein L36a	RPL36A;RPL36A-HI	tr|J3KQN4|J3KQN4	19,58
Eukaryotic translation initiation factor 3 subu	EIF3F	tr|B3KSH1|B3KSH	19,57
60S ribosomal protein L17	RPL17	tr|J3QQT2|J3QQT2	19,53
Cancer/testis antigen family 45 member A3;(	CT45A3;CT45A4;CT	sp|Q8NHU0|CT453	19,52
Plasminogen activator inhibitor 1 RNA-bindin	SERBP1	sp|Q8NC51-3|PAIF	19,47
Double-stranded RNA-binding protein Staufe	STAU1	sp|O95793-2|STAU	19,47
Plakophilin-2	PKP2	sp|Q99959-2|PKP2	19,45
40S ribosomal protein S4, X isoform;40S ribo	RPS4X;RPS4Y2;RF	sp|P62701|RS4X_	19,36
Transducin beta-like protein 3	TBL3	tr|J3KNP2|J3KNP2	19,33
Uncharacterized protein C7orf50	C7orf50	tr|C9JQV0|C9JQV(	19,08
Negative elongation factor C/D	TH1L;NELFCD	tr|H0UI80|H0UI80_	19,07
60S ribosomal protein L28	RPL28	tr|H0YLP6|H0YLP6	18,99
60S ribosomal protein L29	RPL29	sp|P47914|RL29_	18,96
60S ribosomal protein L24	RPL24	tr|C9JXB8|C9JXB8	18,92
Guanine nucleotide-binding protein subunit	GNB2L1	sp|P63244|GBLP_	18,85
Actin-like protein 6A	ACTL6A	sp|O96019-2|ACL6	18,84
Polyadenylate-binding protein;Polyadenylate	PABPC4	tr|B1ANR0|B1ANR(	18,71
Eukaryotic translation initiation factor 3 subu	EIF3J	sp|O75822|EIF3J_	18,71
Unconventional prefoldin RPB5 interactor 1	URI1	tr|F8W9T0|F8W9T(	18,68
40S ribosomal protein S27;40S ribosomal pr	RPS27L	tr|C9JLI6|C9JLI6_H	18,62
Eukaryotic translation initiation factor 3 subu	EIF3I	sp|Q13347|EIF3I_	18,25
Caprin-1	CAPRIN1	sp|Q14444-2|CAPI	18,24
60S ribosomal protein L10a	RPL10A	sp|P62906|RL10A	18,14
Heterogeneous nuclear ribonucleoprotein U	HNRNPU	sp|Q00839-2|HNRI	18,08
Eukaryotic translation initiation factor 3 subu	EIF3L	tr|B0QY89|B0QY8	18,07
Angiomotin-like protein 1	AMOTL1	sp|Q8IY63-2|AMOI	18,00

Interferon-related developmental regulator 1	IFRD1	tr|C9JLG5|C9JLG5	17,99
Uncharacterized protein C11orf98	C11orf98	tr|E9PLD3|E9PLD3	17,98
SWI/SNF-related matrix-associated actin-dep	SMARCE1	tr|B4DGM3|B4DGM	17,95
Helicase SKI2W	SKIV2L	tr|F5H7B0|F5H7B0	17,94
Probable ATP-dependent RNA helicase DD)	DDX17	tr|H3BLZ8|H3BLZ8	17,79
Ubiquitin-associated protein 2-like	UBAP2L	sp|Q14157-4|UBP2	17,58
Pre-mRNA-splicing factor SPF27	BCAS2	sp|O75934|SPF27	17,49
Negative elongation factor B	NELFB	sp|Q8WX92|NELFI	17,33
Eukaryotic translation initiation factor 6	EIF6	sp|P56537|IF6_HU	17,32
Probable ATP-dependent RNA helicase DD)	DDX5	tr|J3KTA4|J3KTA4	17,30
UPF0568 protein C14orf166	C14orf166	sp|Q9Y224|CN166	17,20
Transcription factor ETV6	ETV6	sp|P41212|ETV6_	17,12
Heterogeneous nuclear ribonucleoprotein A	HNRNPA1;HNRNPA	tr|F8W6I7|F8W6I7_	16,96
Dimethyladenosine transferase 1, mitochonc	TFB1M	sp|Q8WVM0|TFB1	16,95
Eukaryotic translation initiation factor 3 subu	EIF3K	tr|K7ES31|K7ES31	16,79
Roquin-1	RC3H1	sp|Q5TC82-2|RC3I	16,49
Eukaryotic translation initiation factor 3 subu	EIF3E	sp|P60228|EIF3E_	16,48
Coiled-coil domain-containing protein 137	CCDC137	tr|I3L0U5|I3L0U5_	16,43
Cleavage and polyadenylation specificity fac	CPSF3	tr|G5E9W3|G5E9W	16,30
40S ribosomal protein S21	RPS21	tr|Q8WVC2|Q8WV	16,28
Angiomotin-like protein 2	AMOTL2	sp|Q9Y2J4|AMOL2	16,27
Histone H1.2;Histone H1.3	HIST1H1C;HIST1H1	sp|P16403|H12_H	16,23
Prefoldin subunit 6	PFDN6	sp|O15212|PFD6_	16,09
Heterogeneous nuclear ribonucleoprotein A(	HNRNPA0	sp|Q13151|ROA0_	15,96
Poly(A) RNA polymerase, mitochondrial	MTPAP	sp|Q9NVV4|PAPD	15,63
Nucleolar RNA helicase 2	DDX21	sp|Q9NR30|DDX21	15,49
Eukaryotic translation initiation factor 3 subu	EIF3D	tr|B4DVY1|B4DVY	15,42
Protein FAM98A	FAM98A	sp|Q8NCA5-2|FA9	15,25
Splicing factor 3B subunit 3	SF3B3	sp|Q15393|SF3B3	15,23
Eukaryotic translation initiation factor 3 subu	EIF3G	tr|K7ER90|K7ER90	15,19
Guanine nucleotide-binding protein-like 3	GNL3	sp|Q9BVP2-2|GNL	15,04
tRNA-splicing ligase RtcB homolog	RTCB	sp|Q9Y3I0|RTCB_	14,98
PIH1 domain-containing protein 1	PIH1D1	sp|Q9NWS0|PIHD1	14,95
Staphylococcal nuclease domain-containing	SND1	sp|Q7KZF4|SND1_	14,93
SWI/SNF complex subunit SMARCC1	SMARCC1	sp|Q92922|SMRC1	14,89
SPATS2-like protein	SPATS2L	sp|Q9NUQ6-2|SPS	14,83
La-related protein 4	LARP4	tr|Q96J85|Q96J85_	14,75
Pre-mRNA 3-end-processing factor FIP1	FIP1L1	sp|Q6UN15-5|FIP1	14,65
Coiled-coil domain-containing protein 124	CCDC124	sp|Q96CT7|CC124	14,63
Splicing factor 3B subunit 4	SF3B4	sp|Q15427|SF3B4	14,54
Galectin-3-binding protein	LGALS3BP	sp|Q08380|LG3BF	14,49
Procollagen-lysine,2-oxoglutarate 5-dioxyge	PLOD1	sp|Q02809|PLOD1	14,38
Nuclear cap-binding protein subunit 2	NCBP2	sp|P52298|NCBP2	14,33
Putative ATP-dependent RNA helicase DHX	DHX30	tr|H7BXY3|H7BXY	14,29
Eukaryotic translation initiation factor 5B	EIF5B	sp|O60841|IF2P_H	14,28
Mitogen-activated protein kinase kinase kina	ZAK	sp|Q9NYL2|MLTK_	14,27
ATP-dependent RNA helicase DDX50	DDX50	sp|Q9BQ39|DDX50	14,19
Synaptonemal complex protein SC65	P3H4;LEPREL4	tr|K7ERA3|K7ERA	14,16
U3 small nucleolar ribonucleoprotein protein	MPHOSPH10	sp|O00566|MPP10	14,12
Serine/threonine-protein phosphatase PGAI	PGAM5	sp|Q96HS1-2|PGA	14,09
Prohibitin	PHB	sp|P35232|PHB_H	14,07
Transcription elongation factor SPT6	SUPT6H	sp|Q7KZ85|SPT6H	13,95
Small nuclear ribonucleoprotein Sm D2	SNRPD2	tr|K7ERG4|K7ERG	13,93
Signal recognition particle 54 kDa protein	SRP54	tr|G3V4F7|G3V4F7	13,89
Serine/arginine-rich splicing factor 5	SRSF5	sp|Q13243-3|SRSI	13,88
PDZ and LIM domain protein 7	PDLIM7	sp|Q9NR12|PDLI7_	13,80

Splicing factor 45	RBM17	sp|Q96I25|SPF45_	13,79
Prefoldin subunit 2	PFDN2	sp|Q9UHV9|PFD2_	13,71
Zinc finger protein ubi-d4	DPF2	tr|J3KMZ8|J3KMZ8	13,67
Signal recognition particle subunit SRP72	SRP72	sp|O76094-2|SRP7	13,66
Ubiquitin carboxyl-terminal hydrolase 10	USP10	sp|Q14694|UBP10	13,65
Histone H1x	H1FX	sp|Q92522|H1X_H	13,62
Exosome complex component MTR3	EXOSC6	sp|Q5RKV6|EXOS	13,54
Enhancer of rudimentary homolog	ERH	sp|P84090|ERH_H	13,48
40S ribosomal protein S30	FAU	tr|E9PR30|E9PR30	13,46
Probable rRNA-processing protein EBP2	EBNA1BP2	tr|H7C2Q8|H7C2Q	13,38
RNA-binding motif protein, X chromosome;R	RBMX	sp|P38159|RBMX_	13,36
NEDD4 family-interacting protein 1	NDFIP1	sp|Q9BT67|NFIP1_	13,17
WD repeat-containing protein 61;WD repeat-	WDR61	tr|H0YN81|H0YN81	13,14
Endothelial differentiation-related factor 1	EDF1	sp|O60869-2|EDF1	13,03
Prohibitin-2	PHB2	tr|F5GY37|F5GY37	13,01
Nucleolin	NCL	sp|P19338|NUCL_	13,00
	ASNSD1	tr|L0R819|L0R819	12,99
ELAV-like protein 1	ELAVL1	sp|Q15717|ELAV1	12,81
Pre-mRNA-processing factor 19	PRPF19	sp|Q9UMS4|PRP1	12,72
Transcription elongation factor B polypeptide	TCEB2	tr|B8ZZU8|B8ZZU8	12,69
WW domain-binding protein 2	WBP2	tr|K7EIJ0|K7EIJ0_	12,66
Eukaryotic initiation factor 4A-I;Eukaryotic ini	EIF4A1;EIF4A2	sp|P60842|IF4A1_	12,64
Ubiquitin carboxyl-terminal hydrolase 24	USP24	sp|Q9UPU5|UBP24	12,63
GPN-loop GTPase 3	GPN3	sp|Q9UHW5|GPN3	12,42
Putative 40S ribosomal protein S26-like 1;40	RPS26P11;RPS26	sp|Q5JNZ5|RS26L	12,38
Integral membrane protein DGCR2/IDD	DKFZp686I1730;DG	tr|Q5CZ70|Q5CZ70	12,28
SWI/SNF-related matrix-associated actin-dep	SMARCD1	sp|Q96GM5|SMRD	12,26
Interferon-inducible double-stranded RNA-de	PRKRA	sp|O75569-3|PRKI	12,23
Pre-mRNA-splicing factor ATP-dependent RN	DHX15	sp|O43143|DHX15	12,10
Glutamate-rich WD repeat-containing protein	GRWD1	sp|Q9BQ67|GRWD	12,09
Heterogeneous nuclear ribonucleoproteins C	HNRNPC	tr|G3V4W0|G3V4W	12,08
RNA-binding protein 34	RBM34	tr|A2A2V2|A2A2V2	11,99
Zinc finger protein 638	ZNF638	sp|Q14966-4|ZN63	11,85
Serine/threonine-protein phosphatase 1 regu	PPP1R10	sp|Q96QC0|PP1RA	11,83
La-related protein 1	LARP1	sp|Q6PKG0|LARP	11,72
Protein SDA1 homolog	SDAD1	sp|Q9NVU7|SDA1_	11,71
Nicotinamide/nicotinic acid mononucleotide a	NMNAT1	sp|Q9HAN9|NMNA	11,67
MKI67 FHA domain-interacting nucleolar pho	NIFK	sp|Q9BYG3|MK67I	11,63
DNA topoisomerase 2-alpha	TOP2A	sp|P11388|TOP2A	11,57
Non-POU domain-containing octamer-binding	NONO	sp|Q15233-2|NONO	11,52
Probable global transcription activator SNF2L	SMARCA2	sp|P51531-2|SMCA	11,44
Cleavage and polyadenylation specificity fac	CPSF2	sp|Q9P2I0|CPSF2_	11,35
Insulin-like growth factor 2 mRNA-binding pro	IGF2BP2	tr|F8W930|F8W930	11,34
Heterogeneous nuclear ribonucleoprotein Q	SYNCRIP	sp|O60506-2|HNRP	11,31
2,4-dienoyl-CoA reductase, mitochondrial	DECR1	tr|B7Z6B8|B7Z6B8	11,28
NF-kappa-B-repressing factor	NKRF	sp|O15226|NKRF_	11,28
Insulin-like growth factor 2 mRNA-binding pro	IGF2BP1	sp|Q9NZI8|IF2B1_	11,27
Transcription factor BTF3	BTF3	tr|H0Y9Y1|H0Y9Y1	11,23
Putative ATP-dependent RNA helicase DHX	DHX57	sp|Q6P158|DHX57	11,15
Protein transport protein Sec61 subunit beta	SEC61B	sp|P60468|SC61B	11,06
Ribosome-binding protein 1	RRBP1	sp|Q9P2E9|RRBP1	10,99
Heterogeneous nuclear ribonucleoprotein R	HNRNPR	sp|O43390-3|HNRP	10,98
Exosome complex component RRP41	EXOSC4	sp|Q9NPD3|EXOS	10,97
Ataxin-2	ATXN2	tr|H0YH87|H0YH87	10,93
SWI/SNF-related matrix-associated actin-dep	SMARCB1	sp|Q12824|SNF5_	10,83
Eukaryotic translation initiation factor 4 gamr	EIF4G1	tr|E7EX73|E7EX73	10,77

Cyclin-dependent kinase 9	CDK9	sp|P50750|CDK9_	10,72
tRNA (cytosine(34)-C(5))-methyltransferase	NSUN2	sp|Q08J23-2|NSUN	10,57
DNA topoisomerase 1	TOP1	sp|P11387|TOP1_	10,54
U2 small nuclear ribonucleoprotein B	SNRPB2	sp|P08579|RU2B_	10,46
Target of EGR1 protein 1	TOE1	sp|Q96GM8|TOE1_	10,43
AT-rich interactive domain-containing protein	ARID1A	sp|O14497|ARI1A_	10,37
Signal recognition particle 14 kDa protein	SRP14	sp|P37108|SRP14	10,35
ATP-binding cassette sub-family F member 1	ABCF1	sp|Q8NE71-2|ABC	10,35
Lysine--tRNA ligase	KARS	sp|Q15046|SYK_H	10,34
Cytoskeleton-associated protein 4	CKAP4	sp|Q07065|CKAP4	10,31
Heterochromatin protein 1-binding protein 3	HP1BP3	sp|Q5SSJ5|HP1B3	10,31
Microprocessor complex subunit DGCR8	DGCR8	sp|Q8WYQ5-3|DG(	10,29
Nascent polypeptide-associated complex sul	NACA	tr|F8VZJ2|F8VZJ2_	10,27
Zinc finger protein 622	ZNF622	sp|Q969S3|ZN622	10,26
Sphingosine-1-phosphate lyase 1	SGPL1	sp|O95470|SGPL1	10,21
ATP-dependent RNA helicase DHX29	DHX29	sp|Q7Z478|DHX29	10,20
GPN-loop GTPase 1	GPN1	sp|Q9HCN4-4|GPN	10,11
RNA polymerase II subunit A C-terminal dom	CTDP1	sp|Q9Y5B0|CTDP1	10,01
Serine/arginine-rich splicing factor 3	SRSF3	tr|B4E241|B4E241	9,98
Pleiotropic regulator 1	PLRG1	tr|A8MW61|A8MW6	9,91
Small nuclear ribonucleoprotein Sm D3	SNRPD3	tr|B4DJP7|B4DJP7	9,84
Heterogeneous nuclear ribonucleoprotein A3	HNRNPA3	sp|P51991|ROA3_	9,81
Histone H1.0;Histone H1.0, N-terminally proc	H1F0	sp|P07305|H10_H	9,81
Heterogeneous nuclear ribonucleoproteins A	HNRNPA2B1	sp|P22626|ROA2_	9,80
Probable ATP-dependent RNA helicase DD)	DDX27	sp|Q96GQ7|DDX2	9,71
InaD-like protein	INADL	sp|Q8NI35|INADL_	9,71
THO complex subunit 4	ALYREF	tr|E9PB61|E9PB61	9,65
Leucine-rich repeat-containing protein 59	LRRC59	sp|Q96AG4|LRC59	9,41
Serine/arginine-rich splicing factor 10	SRSF10	tr|Q5JRI1|Q5JRI1_	9,39
Fragile X mental retardation protein 1	FMR1	sp|Q06787-6|FMR1	9,37
Transcription elongation factor B polypeptide	TCEB1	tr|R4GMY8|R4GMY	9,35
DnaJ homolog subfamily C member 21	DNAJC21	sp|Q5F1R6|DJC21	9,32
N-acetyltransferase 10	NAT10	tr|E7ESU4|E7ESU4	9,31
SWI/SNF complex subunit SMARCC2	SMARCC2	sp|Q8TAQ2|SMRC	9,29
Heterogeneous nuclear ribonucleoprotein D(	HNRNPD	tr|D6RAF8|D6RAF8	9,16
PERQ amino acid-rich with GYF domain-cont	GIGYF2	sp|Q6Y7W6-4|PER	9,13
Peptidyl-prolyl cis-trans isomerase B	PPIB	sp|P23284|PPIB_H	9,10
Apoptosis-stimulating of p53 protein 2	TP53BP2	sp|Q13625-2|ASPP	9,08
Serine/arginine-rich splicing factor 2	SRSF2	tr|J3QL05|J3QL05	9,05
Aspartyl/asparaginyl beta-hydroxylase	ASPH	sp|Q12797-10|ASP	9,03
Importin subunit alpha-1	KPNA2	sp|P52292|IMA1_H	8,96
pre-mRNA 3 end processing protein WDR33	WDR33	sp|Q9C0J8|WDR33	8,94
Negative elongation factor E	NELFE	sp|P18615|NELFE	8,90
Constitutive coactivator of PPAR-gamma-like	FAM120A	sp|Q9NZB2-4|F12(	8,81
60S ribosomal protein L26-like 1	RPL26L1	sp|Q9UNX3|RL26L	8,78
Superkiller viralicidic activity 2-like 2	SKIV2L2	sp|P42285|SK2L2	8,76
BRCA1-A complex subunit BRE	BRE	tr|F8W733|F8W733	8,75
Replication factor C subunit 1	RFC1	sp|P35251-2|RFC1	8,74
Elongation factor 1-alpha 2	EEF1A2	sp|Q05639|EF1A2	8,73
Calcium homeostasis endoplasmic reticulum	CHERP	tr|J3QK89|J3QK89	8,72
28S ribosomal protein S29, mitochondrial	DAP3	tr|V9GZ03|V9GZ03	8,72
Testis-specific Y-encoded-like protein 5	TSPYL5	sp|Q86VY4|TSYL5	8,68
Probable 28S rRNA (cytosine(4447)-C(5))-me	NOP2	sp|P46087-2|NOP2	8,68
Programmed cell death protein 6	PDCD6	sp|O75340-2|PDCI	8,60
Aspartate--tRNA ligase, cytoplasmic	DARS	sp|P14868|SYDC_	8,60
Delta(24)-sterol reductase	DHCR24	tr|H7C4B7|H7C4B7	8,59

Protein LSM14 homolog B	LSM14B	tr|Q5TBP9|Q5TBP!	8,58
Histone H2B type 2-E;Histone H2B type 1-B	HIST2H2BE;HIST1H	sp|Q16778|H2B2E	8,51
X-ray repair cross-complementing protein 5	XRCC5	sp|P13010|XRCC5	8,48
Tubby-related protein 4	TULP4	sp|Q9NRJ4|TULP4	8,47
U3 small nucleolar RNA-associated protein 1	UTP18	sp|Q9Y5J1|UTP18	8,45
SWI/SNF-related matrix-associated actin-dep	SMARCD2	tr|J3KMX2|J3KMX2	8,43
Protein regulator of cytokinesis 1	PRC1	tr|F8W9B5|F8W9B	8,39
Cleavage and polyadenylation specificity fac	CPSF1	sp|Q10570|CPSF1	8,39
U4/U6.U5 tri-snRNP-associated protein 1	SART1	sp|O43290|SNUT1	8,38
Heterogeneous nuclear ribonucleoprotein L	HNRNPL	sp|P14866|HNRPL	8,36
Histone H2A;Histone H2A.V;Histone H2A.Z	H2AFV;H2AFZ	tr|C9J0D1|C9J0D1_	8,35
Arginine--tRNA ligase, cytoplasmic	RARS	sp|P54136|SYRC_	8,33
YTH domain-containing family protein 3	YTHDF3	tr|R4GN55|R4GN5	8,17
Probable ATP-dependent RNA helicase YTH	YTHDC2	sp|Q9H6S0|YTDC2	8,09
Protein lin-7 homolog C	LIN7C	sp|Q9NUP9|LIN7C	7,89
SRSF protein kinase 1	SRPK1	tr|Q5R363|Q5R363	7,85
Regulator of nonsense transcripts 1	UPF1	sp|Q92900-2|RENT	7,78
Protein LYRIC	MTDH	sp|Q86UE4|LYRIC	7,76
Splicing factor 3B subunit 5	SF3B5	sp|Q9BWJ5|SF3B5	7,76
Importin subunit alpha-4	KPNA3	sp|O00505|IMA4_I	7,72
Heterogeneous nuclear ribonucleoprotein H;	HNRNPH1;HNRNPH	tr|G8JLB6|G8JLB6	7,66
Cyclin-T1	CCNT1	sp|O60563|CCNT1	7,63
Transcription initiation factor IIB	GTF2B	sp|Q00403|TF2B_I	7,60
WD repeat-containing protein 89	WDR89	tr|G3V4B8|G3V4B	7,60
Heterogeneous nuclear ribonucleoprotein H3	HNRNPH3	tr|B4DHY1|B4DHY	7,60
SRSF protein kinase 2;SRSF protein kinase	SRPK2	sp|P78362|SRPK2	7,54
3-5 exoribonuclease 1	ERI1	sp|Q8IV48|ERI1_H	7,48
Histone H2A type 1-J;Histone H2A type 1-H;	HIST1H2AJ;HIST1H	sp|Q99878|H2A1J_	7,45
ATP-binding cassette sub-family F member 2	ABCF2	sp|Q9UG63|ABCF2	7,45
Glutamine--tRNA ligase	QARS	sp|P47897|SYQ_H	7,40
Dynein light chain 2, cytoplasmic	DYNLL2	sp|Q96FJ2|DYL2_I	7,40
KH domain-containing, RNA-binding, signal t	KHDRBS1	sp|Q07666-3|KHDI	7,35
Serine/threonine-protein phosphatase;Serin	PPP1CA	tr|E9PMD7|E9PMD	7,35
Putative tRNA (cytidine(32)/guanosine(34)-2	FTSJ1	sp|Q9UET6-2|TRM	7,34
Histone H2B;Histone H2B type 1-L;Histone H	HIST1H2BN;HIST1H	tr|U3KQK0|U3KQK	7,34
U1 small nuclear ribonucleoprotein 70 kDa	SNRNP70	sp|P08621-2|RU17	7,33
Tight junction protein ZO-1	TJP1	tr|G5E9E7|G5E9E	7,27
ATP-dependent RNA helicase DDX18	DDX18	sp|Q9NVP1|DDX18	7,20
Replication factor C subunit 4	RFC4	sp|P35249|RFC4_I	7,18
Serine beta-lactamase-like protein LACTB, m	LACTB	sp|P83111-2|LACT	7,15
pre-rRNA processing protein FTSJ3	FTSJ3	sp|Q8IY81|SPB1_I	7,14
60S ribosomal protein L36a-like	RPL36AL	sp|Q969Q0|RL36L	7,10
Histone-binding protein RBBP4;Histone-bind	RBBP4;RBBP7	sp|Q09028-3|RBBI	7,08
Pescadillo homolog	PES1	tr|B5MCF9|B5MCF	7,07
ATPase family AAA domain-containing prote	ATAD3A;ATAD3B	sp|Q9NVI7-2|ATD3	7,02
Ubiquitin carboxyl-terminal hydrolase 15	USP15	sp|Q9Y4E8|UBP15	7,01
Ubiquitin-associated protein 2	UBAP2	tr|E7EWG4|E7EW(	6,98
rRNA 2-O-methyltransferase fibrillarin	FBL	sp|P22087|FBRL_I	6,98
Dehydrogenase/reductase SDR family memb	DHRS2	sp|Q13268|DHRS2	6,94
Melanoma-associated antigen 4;Melanoma-a	MAGEA4;MAGEA8	sp|P43358|MAGA4	6,89
MAGUK p55 subfamily member 5	MPP5	sp|Q8N3R9|MPP5_	6,89
Leucine--tRNA ligase, cytoplasmic	LARS	tr|F5H698|F5H698_	6,84
Transformer-2 protein homolog alpha	TRA2A	tr|B4DQI6|B4DQI6_	6,84
Vigilin	HDLBP	sp|Q00341|VIGLN_	6,83
Deoxynucleotidyltransferase terminal-interact	DNTTIP1	sp|Q9H147|TDIF1_	6,73
Nucleolar protein 16	NOP16	sp|Q9Y3C1|NOP16	6,73

Isoleucine--tRNA ligase, cytoplasmic	IARS	sp|P41252|SYIC_H	6,67
Nucleolar transcription factor 1	UBTF	tr|E9PKP7|E9PKP7	6,66
Heat shock 70 kDa protein 1A	HSPA1A	sp|P08107-2|HSP7	6,63
U2 snRNP-associated SURP motif-containing	U2SURP	sp|O15042-2|SR14	6,58
Negative elongation factor A	WHSC2;NELFA	tr|B3KSP0|B3KSP0	6,57
Putative helicase MOV-10	MOV10	tr|Q5JR04|Q5JR04	6,51
Uncharacterized protein CXorf67	CXorf67	sp|Q86X51|CX067	6,51
Protein HEXIM1	HEXIM1	sp|O94992|HEXI1_	6,45
Kinesin-like protein KIFC1	KIFC1	sp|Q9BW19|KIFC1	6,44
Pre-mRNA-processing-splicing factor 8	PRPF8	sp|Q6P2Q9|PRP8_	6,43
Aminoacyl tRNA synthase complex-interactin	AIMP2	sp|Q13155|AIMP2_	6,43
Multiple myeloma tumor-associated protein 2	MMTAG2	sp|Q9BU76-4|MMT	6,36
Nuclear export mediator factor NEMF	NEMF	sp|O60524-4|NEMI	6,27
FACT complex subunit SSRP1	SSRP1	sp|Q08945|SSRP1	6,25
Nucleolar protein 58	NOP58	sp|Q9Y2X3|NOP58	6,23
Probable ATP-dependent RNA helicase DD)	DDX6	sp|P26196|DDX6_	6,14
Zinc finger CCCH-type antiviral protein 1	ZC3HAV1	sp|Q7Z2W4-2|ZCC	6,13
AT-rich interactive domain-containing protein	ARID1B	tr|G3XAA0|G3XAA	6,10
Ribosome biogenesis protein BMS1 homolog	BMS1	sp|Q14692|BMS1_	6,08
Splicing factor 3B subunit 2	SF3B2	sp|Q13435|SF3B2	6,06
U1 small nuclear ribonucleoprotein C	SNRPC	sp|P09234|RU1C_	6,06
Caveolin-1;Caveolin	CAV1	sp|Q03135|CAV1_	6,05
RRP12-like protein	RRP12	sp|Q5JTH9-2|RRP1	5,97
Histone deacetylase 1	HDAC1	sp|Q13547|HDAC1	5,91
Bifunctional glutamate/proline--tRNA ligase;(	EPRS	sp|P07814|SYEP_	5,88
Histone lysine demethylase PHF8	PHF8	tr|H0Y3N9|H0Y3N9	5,80
Replication factor C subunit 2	RFC2	tr|H7C5S7|H7C5S7	5,80
Exosome component 10	EXOSC10	sp|Q01780|EXOSX	5,74
Cell growth-regulating nucleolar protein	LYAR	sp|Q9NX58|LYAR_	5,73
Aminoacyl tRNA synthase complex-interactin	AIMP1	sp|Q12904|AIMP1_	5,71
Segment polarity protein dishevelled homolo	DVL2	sp|O14641|DVL2_	5,58
Transcription intermediary factor 1-beta	TRIM28	sp|Q13263|TIF1B_	5,55
DnaJ homolog subfamily C member 13	DNAJC13	sp|O75165|DJC13_	5,53
Ubiquitin-40S ribosomal protein S27a;Ubiqui	RPS27A;UBB;UBC;I	sp|P62979|RS27A	5,52
Ribosome production factor 2 homolog	RPF2	sp|Q9H7B2|RPF2_	5,51
RNA-binding protein 28	RBM28	sp|Q9NW13|RBM2	5,29
WD repeat-containing protein 6	WDR6	tr|E9PDU5|E9PDU5	5,27
Ataxin-2-like protein	ATXN2L	tr|H3BUF6|H3BUF6	5,25
Chromatin target of PRMT1 protein	CHTOP	sp|Q9Y3Y2-4|CHT(	5,23
Matrin-3	MATR3	tr|D6REM6|D6REM	5,23
Activating signal cointegrator 1 complex sub	ASCC3	sp|Q8N3C0|ASCC3	5,21
Nucleolar GTP-binding protein 2	GNL2	sp|Q13823|NOG2_	5,20
G patch domain-containing protein 4	GPATCH4	tr|E9PAV9|E9PAV9	5,16
Ribonuclease 3	DROSHA	sp|Q9NRR4|RNC_I	5,16
Heterogeneous nuclear ribonucleoprotein K	HNRNPK	tr|Q5T6W5|Q5T6W	5,15
Squamous cell carcinoma antigen recognize	SART3	tr|F8VV04|F8VV04	5,14
CAD protein;Glutamine-dependent carbamo	CAD	tr|F8VPD4|F8VPD4	5,14
Putative eukaryotic translation initiation facto	EIF2S3L;EIF2S3	tr|F8W810|F8W810	5,13
Histone H4	HIST1H4A	sp|P62805|H4_HU	5,11
Histone deacetylase 2	HDAC2	tr|J3KPW7|J3KPW	5,11
Serine/threonine-protein phosphatase PP1-I	PPP1CB	sp|P62140|PP1B_	5,11
FACT complex subunit SPT16	SUPT16H	sp|Q9Y5B9|SP16H	5,06
Probable global transcription activator SNF2I	SMARCA1;SMARCA	sp|P28370-2|SMC/	5,05
V-type proton ATPase 116 kDa subunit a isc	TCIRG1	sp|Q13488-2|VPP3	5,02
DNA-dependent protein kinase catalytic sub	PRKDC	tr|E7EUY0|E7EUY(	5,01
Melanoma-associated antigen B2	MAGEB2	sp|O15479|MAGB2	4,98

Programmed cell death protein 4	PDCD4	sp|Q53EL6-2|PDCI	4,92
Myb-binding protein 1A	MYBBP1A	sp|Q9BQG0|MBB1	4,88
ATP-dependent RNA helicase DDX3X;ATP-(	DDX3X;DDX3Y	sp|O00571-2|DDX3	4,87
Splicing factor 3B subunit 1	SF3B1	sp|O75533|SF3B1	4,86
General transcription factor 3C polypeptide 1	GTF3C1	sp|Q12789-3|TF3C	4,86
RNA-binding protein 39	RNPC2;RBM39	tr|E1P5S2|E1P5S2	4,85
Histone H3;Histone H3.2;Histone H3.1t;Histo	H3F3B;H3F3A;HIST	tr|K7EMV3|K7EMV	4,84
BAG family molecular chaperone regulator 2	BAG2	tr|B4DXE2|B4DXE	4,84
U5 small nuclear ribonucleoprotein 200 kDa	SNRNP200	sp|O75643|U520_	4,83
E3 ubiquitin-protein ligase RING2;E3 ubiquit	RNF2;RING1	sp|Q99496|RING2	4,82
AT-hook DNA-binding motif-containing protei	AHDC1	sp|Q5TGY3|AHDC	4,79
Protein S100-A2	S100A2	tr|R4GN49|R4GN4	4,70
Spermatogenesis-associated serine-rich prot	SPATS2	sp|Q86XZ4|SPAS2	4,65
DnaJ homolog subfamily A member 1	DNAJA1	sp|P31689|DNJA1	4,64
Polypyrimidine tract-binding protein 1	PTBP1	sp|P26599|PTBP1	4,63
Periodic tryptophan protein 2 homolog	PWP2	sp|Q15269|PWP2_	4,53
Thymidine kinase;Thymidine kinase, cytosoli	TK1	tr|K7ES52|K7ES52	4,46
H/ACA ribonucleoprotein complex subunit 1	GAR1	sp|Q9NY12-2|GAR	4,38
Cytoplasmic FMR1-interacting protein 1;Cyto	CYFIP1;CYFIP2	sp|Q7L576|CYFP1	4,37
Trifunctional enzyme subunit beta, mitochon	HADHB	tr|B4E2W0|B4E2W	4,36
DNA dC->dU-editing enzyme APOBEC-3B;D	APOBEC3B;APOBE	tr|B0QYD3|B0QYD	4,34
Pre-mRNA-processing factor 6	PRPF6	sp|O94906-2|PRP6	4,33
Transcription factor A, mitochondrial	TFAM	tr|H7BYN3|H7BYN	4,32
60S ribosomal export protein NMD3	NMD3	tr|C9JA08|C9JA08	4,31
Eukaryotic translation initiation factor 2 subu	EIF2S1	sp|P05198|IF2A_H	4,30
116 kDa U5 small nuclear ribonucleoprotein	EFTUD2	sp|Q15029-2|U5S1	4,29
Eukaryotic initiation factor 4A-III;Eukaryotic ir	EIF4A3	sp|P38919|IF4A3_	4,27
ELM2 and SANT domain-containing protein	ELMSAN1	tr|F6RU81|F6RU81	4,27
Cytochrome c oxidase subunit NDUFA4	NDUFA4	sp|O00483|NDUA4	4,23
TATA-binding protein-associated factor 2N	TAF15	sp|Q92804-2|RBP	4,20
Nucleosome assembly protein 1-like 1	NAP1L1	tr|H0YHC3|H0YHC	4,19
Importin-5	IPO5	tr|H0Y8C6|H0Y8C6	4,16
Eukaryotic translation initiation factor 3 subu	EIF3M	sp|Q7L2H7|EIF3M	4,15
Eukaryotic translation elongation factor 1 ep	EEF1E1;EEF1E1-BI	tr|H0YAL7|H0YAL7	4,14
NHP2-like protein 1;NHP2-like protein 1, N-te	NHP2L1	tr|B1AHD1|B1AHD	4,12
Complement component 1 Q subcomponent	C1QBP	sp|Q07021|C1QBP	4,12
DNA topoisomerase 2-beta;DNA topoisomera	TOP2B	sp|Q02880-2|TOP2	4,02
Ankyrin repeat domain-containing protein 17	ANKRD17;ANKHD1	tr|H0YM23|H0YM2	4,01
Ribosomal RNA processing protein 1 homolo	RRP1B	sp|Q14684-2|RRP1	4,00
Pre-rRNA-processing protein TSR1 homolog	TSR1	sp|Q2NL82|TSR1_	3,99
TRMT1-like protein	TRMT1L	sp|Q7Z2T5|TRM1L	3,96
Luc7-like protein 3	LUC7L3	tr|B4DJ96|B4DJ96	3,90
Mitochondrial import inner membrane translo	TIMM50	tr|M0R0C3|M0R0C	3,89
Protein arginine N-methyltransferase 1	PRMT1	tr|E9PKG1|E9PKG	3,88
Heat shock cognate 71 kDa protein	HSPA8	sp|P11142|HSP7C	3,86
Abl interactor 1	ABI1	tr|F5H1G9|F5H1G	3,85
Brain-specific angiogenesis inhibitor 1-assoc	BAIAP2	sp|Q9UQB8-2|BAII	3,81
Splicing factor U2AF 65 kDa subunit	U2AF2	tr|K7ENG2|K7ENG	3,81
Guanine nucleotide-binding protein G(s) sub	GNAS;GNAT1;GNAI	sp|P63092-3|GNAS	3,80
Signal recognition particle subunit SRP68	SRP68	sp|Q9UHB9-4|SRP	3,79
Uncharacterized protein KIAA1522	KIAA1522	sp|Q9P206-2|K152	3,76
Eukaryotic translation initiation factor 4 gamr	EIF4G2	tr|D3DQV9|D3DQV	3,75
Trifunctional enzyme subunit alpha, mitochor	HADHA	sp|P40939|ECHA_	3,75
Heterogeneous nuclear ribonucleoprotein F;	HNRNPF	sp|P52597|HNRPF	3,73
RNA-binding protein EWS	EWSR1	tr|B0QYK0|B0QYK	3,72
Tetratricopeptide repeat protein 37	TTC37	sp|Q6PGP7|TTC37	3,67

UDP-N-acetylglucosamine--peptide N-acetylg	OGT	sp|O15294|OGT1_	3,64
Probable helicase senataxin	SETX	sp|Q7Z333|SETX_	3,58
SWI/SNF-related matrix-associated actin-dep	SMARCB1	tr|G5E975|G5E975	3,55
Poly [ADP-ribose] polymerase 1	PARP1	sp|P09874|PARP1	3,54
Nuclear mitotic apparatus protein 1	NUMA1	tr|H0YFY6|H0YFY6	3,53
D-3-phosphoglycerate dehydrogenase	PHGDH	sp|O43175|SERA_	3,51
Casein kinase II subunit alpha 3;Casein kina	CSNK2A1;CSNK2A	tr|E7EU96|E7EU96	3,50
Nck-associated protein 1	NCKAP1	sp|Q9Y2A7|NCKP1	3,49
Myosin light polypeptide 6	MYL6	tr|F8W1R7|F8W1R7	3,48
T-complex protein 1 subunit gamma	CCT3	tr|B4DUR8|B4DUR8	3,46
Unconventional myosin-Ic	MYO1C	tr|F5H6E2|F5H6E2	3,40
3-hydroxyacyl-CoA dehydrogenase type-2	HSD17B10	sp|Q99714|HCD2_	3,39
Microtubule-associated protein;Microtubule-a	MAP4	tr|E7EVA0|E7EVA0	3,36
Cystathionine beta-synthase	CBS	tr|F5H2U1|F5H2U1	3,34
NEDD4-like E3 ubiquitin-protein ligase WWP2	WWP2	sp|O00308-2|WWP	3,32
Signal transducer and activator of transcripti	STAT3	tr|K7ENL3|K7ENL3	3,29
LINE-1 retrotransposable element ORF1 pro	L1RE1	sp|Q9UN81|LORF1	3,29
RNA-binding protein Raly	RALY	tr|Q5QPM0|Q5QPM	3,27
RNA-binding protein 10	RBM10	sp|P98175-4|RBM1	3,27
Ras GTPase-activating-like protein IQGAP1	IQGAP1	sp|P46940|IQGA1_	3,25
Nuclear fragile X mental retardation-interacti	NUFIP2	sp|Q7Z417|NUFP2	3,24
BolA-like protein 2	BOLA2;BOLA2B	sp|Q9H3K6|BOLA2	3,21
Heat shock protein HSP 90-alpha	HSP90AA1	sp|P07900|HS90A	3,21
Zinc finger CCCH-type antiviral protein 1-like	ZC3HAV1L	sp|Q96H79|ZCCHL	3,20
Dolichyl-diphosphooligosaccharide--protein g	RPN2	tr|Q5JYR4|Q5JYR4	3,19
MAP7 domain-containing protein 1	MAP7D1	sp|Q3KQU3-2|MA7	3,17
Phosphate carrier protein, mitochondrial	SLC25A3	sp|Q00325-2|MPCP	3,16
Calmodulin	CALM1;CALM2;CAL	tr|E7ETZ0|E7ETZ0	3,13
Suppressor of SWI4 1 homolog	PPAN	tr|H7C446|H7C446	3,11
Exosome complex component RRP40	EXOSC3	sp|Q9NQT5-2|EXO	3,10
Serine/arginine repetitive matrix protein 2	SRRM2	sp|Q9UQ35|SRRM	3,10
Wiskott-Aldrich syndrome protein family mem	WASF2	sp|Q9Y6W5|WASF	3,09
Host cell factor 1;HCF N-terminal chain 1;HC	HCFC1	tr|A6NEM2|A6NEM	3,09
Double-stranded RNA-binding protein Staufe	STAU2	tr|E5RJN7|E5RJN7	3,05
E3 ubiquitin/ISG15 ligase TRIM25	TRIM25	sp|Q14258|TRI25_	3,02
Elongation factor 2	EEF2	sp|P13639|EF2_H	3,00
Structural maintenance of chromosomes pro	SMC1A	sp|Q14683|SMC1A	2,99
Protein transport protein Sec24C	SEC24C	tr|E7EP00|E7EP00	2,97
78 kDa glucose-regulated protein	HSPA5	sp|P11021|GRP78	2,96
Multifunctional methyltransferase subunit TR	TRMT112	tr|F5GX77|F5GX77	2,94
Poly(rC)-binding protein 1	PCBP1	sp|Q15365|PCBP1	2,93
Heat shock protein 105 kDa	HSPH1	sp|Q92598-2|HS10	2,92
Putative RNA-binding protein Luc7-like 1	LUC7L	tr|B8ZZ10|B8ZZ10_	2,91
Arrestin domain-containing protein 1	ARRDC1	sp|Q8N5I2|ARRD1	2,86
Growth factor receptor-bound protein 2	GRB2	sp|P62993|GRB2_	2,84
C-1-tetrahydrofolate synthase, cytoplasmic;M	MTHFD1	tr|F5H2F4|F5H2F4_	2,82
Cell division cycle 5-like protein	CDC5L	sp|Q99459|CDC5L	2,81
Kinectin	KTN1	sp|Q86UP2-2|KTN	2,79
Cysteine--tRNA ligase, cytoplasmic	CARS	tr|A8MVQ3|A8MVQ	2,79
Tricarboxylate transport protein, mitochondria	SLC25A1	sp|P53007|TXTP_	2,78
Dihydropyrimidinase-related protein 3	DPYSL3	sp|Q14195-2|DPYL	2,77
Methionine--tRNA ligase, cytoplasmic	MARS	sp|P56192|SYMC_	2,70
Putative RNA-binding protein Luc7-like 2	LUC7L2	sp|Q96HJ9-2|CG05	2,70
Proteasome activator complex subunit 2	PSME2	tr|H0YM70|H0YM70	2,69
ATP-dependent DNA helicase Q4	RECQL4	tr|U3KQ17|U3KQ17	2,69
Proteasome activator complex subunit 1	PSME1	tr|H0YNE3|H0YNE3	2,68

Merlin	NF2	sp|P35240-4|MERL	2,68
Branched-chain-amino-acid aminotransfera	BCAT1	sp|P54687-2|BCAT	2,68
Heat shock protein HSP 90-beta	HSP90AB1	sp|P08238|HS90B	2,66
Serine/threonine-protein phosphatase 2A 65	PPP2R1A;PPP2R1	sp|P30153|2AAA_	2,64
Tubulin alpha-1A chain;Tubulin alpha-1C ch	TUBA1A;TUBA1C;T	sp|Q71U36-2|TBA	2,63
Prolyl 3-hydroxylase 1	LEPRE1	sp|Q32P28|P3H1_	2,61
SHC-transforming protein 1	SHC1	sp|P29353-5|SHC	2,60
Dihydropyrimidinase-related protein 2	DPYSL2	sp|Q16555-2|DPYL	2,59
Thyroid hormone receptor-associated protei	THRAP3	sp|Q9Y2W1|TR150	2,57
Tubulin beta chain	TUBB	tr|Q5JP53|Q5JP53	2,56
Ubiquitin carboxyl-terminal hydrolase 5	USP5	sp|P45974-2|UBP	2,56
14-3-3 protein zeta/delta	YWHAZ	tr|E7EX29|E7EX2	2,55
Actin, cytoplasmic 2;Actin, cytoplasmic 2, N-t	ACTG1;ACTB;ACTA	sp|P63261|ACTG_	2,55
Poly(rC)-binding protein 2;Poly(rC)-binding p	PCBP2;PCBP3	tr|H3BRU6|H3BRU	2,55
ADP/ATP translocase 2;ADP/ATP transloca	SLC25A5;SLC25A4	sp|P05141|ADT2_	2,53
Major vault protein	MVP	sp|Q14764|MVP_H	2,53
Tumor susceptibility gene 101 protein	TSG101	tr|F5H442|F5H442	2,51
La-related protein 7	LARP7	sp|Q4G0J3|LARP7	2,51
Fragile X mental retardation syndrome-relate	FXR2	sp|P51116|FXR2_	2,48
Peflin	PEF1	sp|Q9UBV8|PEF1_	2,47
Annexin A2;Annexin;Putative annexin A2-lik	ANXA2;ANXA2P2	sp|P07355|ANXA2	2,46
Replication factor C subunit 3	RFC3	sp|P40938-2|RFC3	2,45
Nuclear pore complex protein Nup155	NUP155	tr|E9PF10|E9PF10	2,44
Tumor protein p73	TP73	sp|O15350-12|P73	2,43
Signal transducer and activator of transcripti	STAT1	tr|E9PH66|E9PH66	2,41
Splicing factor U2AF 35 kDa subunit	U2AF1	sp|Q01081|U2AF1	2,35
Melanoma-associated antigen D2	MAGED2	tr|Q5H907|Q5H907	2,35
Uncharacterized protein C3orf17	C3orf17	tr|E9PFF8|E9PFF8	2,35
Isoamyl acetate-hydrolyzing esterase 1 hom	IAH1	tr|H7C5G1|H7C5G	2,33
Monofunctional C1-tetrahydrofolate synthas	MTHFD1L	sp|Q6UB35|C1TM_	2,33
F-actin-capping protein subunit alpha-2	CAPZA2	sp|P47755|CAZA2	2,30
Ubiquitin-like modifier-activating enzyme 6	UBA6	sp|A0AVT1-2|UBA	2,25
Filamin-A	FLNA	tr|Q5HY54|Q5HY5	2,25
Heat shock protein beta-1	HSPB1	sp|P04792|HSPB1	2,23
Scaffold attachment factor B1	SAFB	sp|Q15424-2|SAFB	2,23
Dolichol-phosphate mannosyltransferase su	DPM1	tr|E9PHH5|E9PHH	2,22
Treacle protein	TCOF1	tr|J3KQ96|J3KQ96	2,22
Palladin	PALLD	sp|Q8WX93-3|PAL	2,21
	UBBP4	tr|J3QLP7|J3QLP7	2,21
Thioredoxin-dependent peroxide reductase,	PRDX3	tr|E9PH29|E9PH29	2,20
Clathrin heavy chain 1	CLTC	sp|Q00610-2|CLH1	2,20
Fatty acid synthase;[Acyl-carrier-protein] S-a	FASN	sp|P49327|FAS_H	2,19
Far upstream element-binding protein 2	KHSRP	sp|Q92945|FUBP2	2,19
Cofilin-1	CFL1	sp|P23528|COF1_	2,18
Galectin-1	LGALS1	sp|P09382|LEG1_	2,18
Charged multivesicular body protein 1a	CHMP1A	tr|F8VVT7|F8VVT7	2,17
Transgelin-2	TAGLN2	sp|P37802|TAGL2	2,16
T-complex protein 1 subunit zeta-2	CCT6B	sp|Q92526-2|TCPV	2,14
Protein deglycase DJ-1	PARK7	tr|K7ELW0|K7ELW	2,13
LanC-like protein 2	LANCL2	sp|Q9NS86|LANC2	2,12
Hematological and neurological expressed 1	HN1L	tr|H3BMT0|H3BMT	2,11
Acyl-protein thioesterase 2	LYPLA2	tr|Q5QPQ0|Q5QPQ	2,09
Mitotic checkpoint protein BUB3	BUB3	tr|B4DDM6|B4DDM	2,08
Calpain small subunit 1;Calpain small subuni	CAPNS1;CAPNS2	tr|U3KQE2|U3KQE	2,06
Stromal membrane-associated protein 1	SMAP1	tr|E7ETU6|E7ETU6	2,06
Protein PRRC2B	PRRC2B	sp|Q5JSZ5-5|PRC2	2,05

Protein RCC2	RCC2	sp|Q9P258|RCC2_	2,04
14-3-3 protein theta	YWHAQ	sp|P27348|1433T_	2,04
Rho guanine nucleotide exchange factor 2	ARHGEF2	tr|Q5VY93|Q5VY9	2,04
F-actin-capping protein subunit beta	CAPZB	tr|B1AK87|B1AK87	2,04
Tubulin beta-3 chain	TUBB3	sp|Q13509|TBB3_	2,03
Src substrate cortactin	CTTN	sp|Q14247|SRC8_	2,03
Proline synthase co-transcribed bacterial hoı	PROSC	sp|O94903|PROS(	2,03
Carbonyl reductase [NADPH] 1	CBR1	sp|P16152|CBR1_	2,01
Fructose-2,6-bisphosphatase TIGAR	TIGAR	sp|Q9NQ88|TIGAR	2,01
Glutamine--fructose-6-phosphate aminotrans	GFPT1	sp|Q06210-2|GFP	2,00
Kinesin-like protein;Kinesin-like protein KIF2(	KIF2C	tr|B7Z6Q6|B7Z6Q6	2,00
